# Robust Chemometric Machine Learning Framework for Screening-Stage Triage in Antidoping Urine Analysis

**DOI:** 10.1021/acs.analchem.6c02186

**Published:** 2026-09-24

**Authors:** Hana Park, Junghyun Son

**Affiliations:** Doping Control Center, 58975 Korea Institute of Science and Technology, Seoul 02792, Republic of Korea

## Abstract

Initial testing procedure (ITP) screening in antidoping requires rapid interpretation of high-throughput data under analytically complex and operationally demanding decision conditions. We developed a robustness-validated chemometric machine-learning framework—an integrated multivariate modeling workflow—for ITP triage using MassHunter-derived analyte-level peak-area response values for 148 GC–MS/MS analytes from 684 authentic urine casework samples, rather than raw GC–MS/MS chromatograms. Benchmarking 10,920 machine-learning pipelines, we selected a stacking-based ensemble optimized for rare-positive screening under severe class imbalance. On a fixed external-validation hold-out set (*n* = 137), the selected Rank 1 model achieved near-perfect recall across repeated evaluations. When applied post hoc to all 684 samples, the fixed implementation flagged all 56 reference-positive samples. SHAP analysis showed chemically meaningful analyte-response attribution patterns that were not determined solely by feature coverage. Covariate-adjusted analysis showed no independent association with sex, while exploratory sport analysis revealed no consistent sport-related pattern. This framework reduced estimated screening-review time by >99.8% relative to conventional manual review, providing scalable, interpretable support for prioritizing suspicious samples for expert review and confirmatory follow-up in routine regulatory antidoping laboratories.

## Graphical abstract

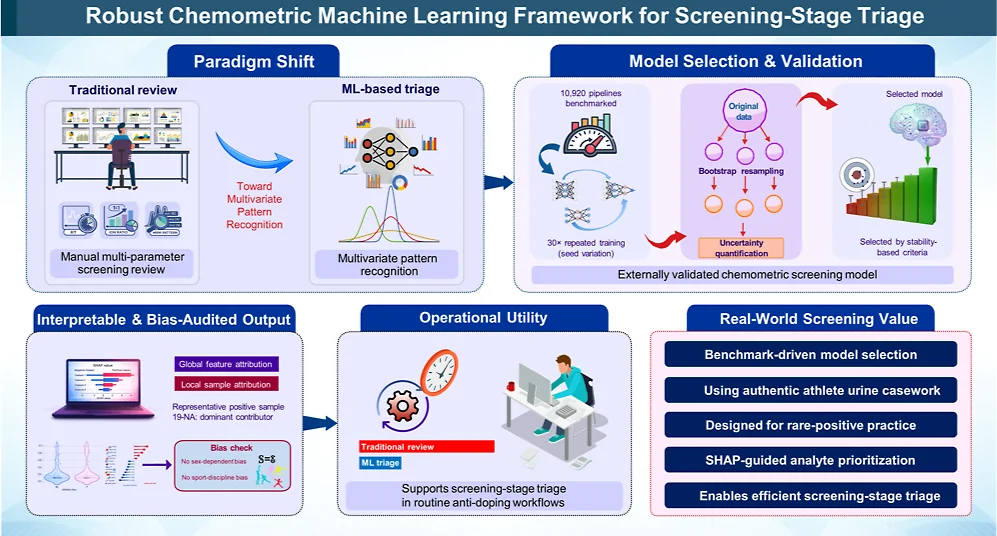

## Introduction

Efforts to enhance athletic performance through prohibited means continue to challenge sports organizations and antidoping laboratories worldwide. As the WADA Prohibited List is updated annually,^1^ antidoping laboratories must maintain broad testing capabilities and continuously adapt their analytical methods.^2,3^

The initial testing procedure (ITP) of doping control is designed for the rapid and simultaneous screening of large numbers of urine samples for a wide range of prohibited substances. Speed and accuracy at this stage are essential for ensuring that samples requiring further evaluation are appropriately prioritized. Traditionally, one or two experienced reviewers independently examine large volumes of chromatographic and mass spectrometric information to detect atypical findings. According to the WADA 2022 Anti-Doping Testing Figures, adverse analytical findings (AAFs) were reported in only 0.77% of samples analyzed.^4^ Reported positive rates and estimated doping prevalence vary across athlete populations and testing contexts.^5,6^ This low rate of AAFs places a disproportionate burden on manual review workflows, which rely heavily on sustained human attention and expert judgment to screen vast data sets. Consequently, a scalable and efficient analytical approach is needed to streamline early-stage data triage.

In routine antidoping laboratories, testing follows a staged workflow in which ITP screening data are first interpreted to identify suspicious analytical profiles, and only flagged samples proceed to compound-specific confirmatory testing and final reporting. Accordingly, the ITP stage serves as a critical gatekeeper: suspicious cases missed during the initial screening may never reach confirmation, whereas excessive flagging can substantially increase the confirmatory workload. Improving the accuracy and consistency of early screening interpretation is therefore essential for reducing the risk of missing such cases while maintaining operational efficiency.

Traditional evaluation methods are limited not only by speed and scale but also by the complexity of the multianalyte interpretation. Analyzing individual analyte signals in isolation cannot adequately reflect the multivariate structures of chromatographic and mass spectrometric data. In antidoping screening, analyte-specific interpretation often requires more than analysis of a single signal, because suspicious findings may depend on the joint consideration of parent analytes, metabolites, marker patterns, chromatographic consistency, ion-based identification criteria, and, for some substance classes, downstream ratio- or profile-based assessment. An analytical framework capable of integrating the full 148-analyte screening profile is therefore needed to capture multivariate relationships that may not be evident from an analyte-by-analyte review. The central analytical challenge in antidoping screening is the reliable recognition of suspicious multianalyte patterns under extreme class imbalance and across multiple substance-specific confirmatory criteria.

Artificial intelligence (AI) has rapidly advanced in analytical chemistry, where it is increasingly used to enhance data interpretation, experimental automation, and predictive modeling.^7–11^ These developments are particularly relevant to antidoping laboratories, in which large-scale MS-based screening generates high-dimensional multianalyte data sets that are difficult to evaluate consistently through manual review alone. Machine-learning (ML) algorithms are particularly well suited to this setting because they can model complex feature interdependencies and nonlinear multivariate signatures associated with atypical samples.

Recent AI-based studies in antidoping have explored several distinct analytical contexts. ML models have been applied to hematological profiles to indirectly detect recombinant human erythropoietin administration^12^ and to identify doping suspicions using an Athlete’s Performance Passport framework.^13^ Metabolomics workflows employing liquid chromatography–mass spectrometry (LC–MS) and nuclear magnetic resonance platforms, combined with ML techniques, have been proposed as promising tools for characterizing doping-related metabolic perturbations and exploring potential long-term biomarkers.^14^ Recent reviews have also surveyed AI and ML applications across doping science, including sample screening, blood-based detection, urine adulteration, and metabolomics-assisted strategies.^14,15^ Although these studies highlight the expanding relevance of AI in the antidoping field, they remain distinct from the analytical environment of ITP workflows, where multianalyte MS-based screening data are used to evaluate large numbers of urine samples. These prior studies also differ substantially from the present study in sample matrix, analytical objective, and validation strategy. For example, one cited screening study separated positive and negative samples using a PCA score-plot approach and did not report standard validation metrics such as accuracy, recall, or AUC, whereas other studies focused on blood biomarkers or urine adulteration rather than multianalyte human urine screening.^15^ Accordingly, machine-learning applications directly addressing human urine MS-based screening data for antidoping triage remain limited, leaving a clear methodological gap that the present work was designed to address.

MS remains the technology of choice for detecting prohibited substances in athlete urine because of its sensitivity, selectivity, and broad analyte coverage.^16^ In the analysis of multianalyte MS data, chemometrics broadly refers to the use of multivariate statistical and mathematical methods to extract, model, and interpret information from chemical measurement data, and related terminology such as chemometric strategies and AI/ML-assisted chemometric analysis is well established in the analytical-chemistry literature.^17,18^ Such AI/ML-assisted multivariate approaches have also been applied to MS-based metabolomics using stacking machine learning.^19^ The term “chemometric machine-learning framework” used in this study denotes an integrated multivariate modeling workflow rather than a proprietary platform. In the present implementation, this workflow comprised MassHunter-derived GC–MS/MS peak-area feature extraction, preprocessing, supervised classification, decision-threshold optimization, robustness evaluation, and interpretability analysis.

In this study, we developed a machine-learning framework for sample-level ITP triage using integrated multianalyte GC–MS/MS peak-area response profiles. The model classifies urine samples as negative or suspicious and prioritizes those classified as suspicious for subsequent expert confirmation under WADA-compliant antidoping procedures. The framework was designed to reduce manual interpretation time, improve consistency during early stage triage, support scalable screening operations, and maintain expert oversight for the final reporting decisions.

## Experimental Section

Figure 1 summarizes the chemometric workflow integrating GC–MS/MS data curation with model development, validation, and interpretation. The workflow comprises three stages: stage 1, data preparation and partitioning; stage 2, machine-learning pipeline optimization; and stage 3, statistical validation and chemical interpretation. Methodological details are provided below.

**Figure 1:**
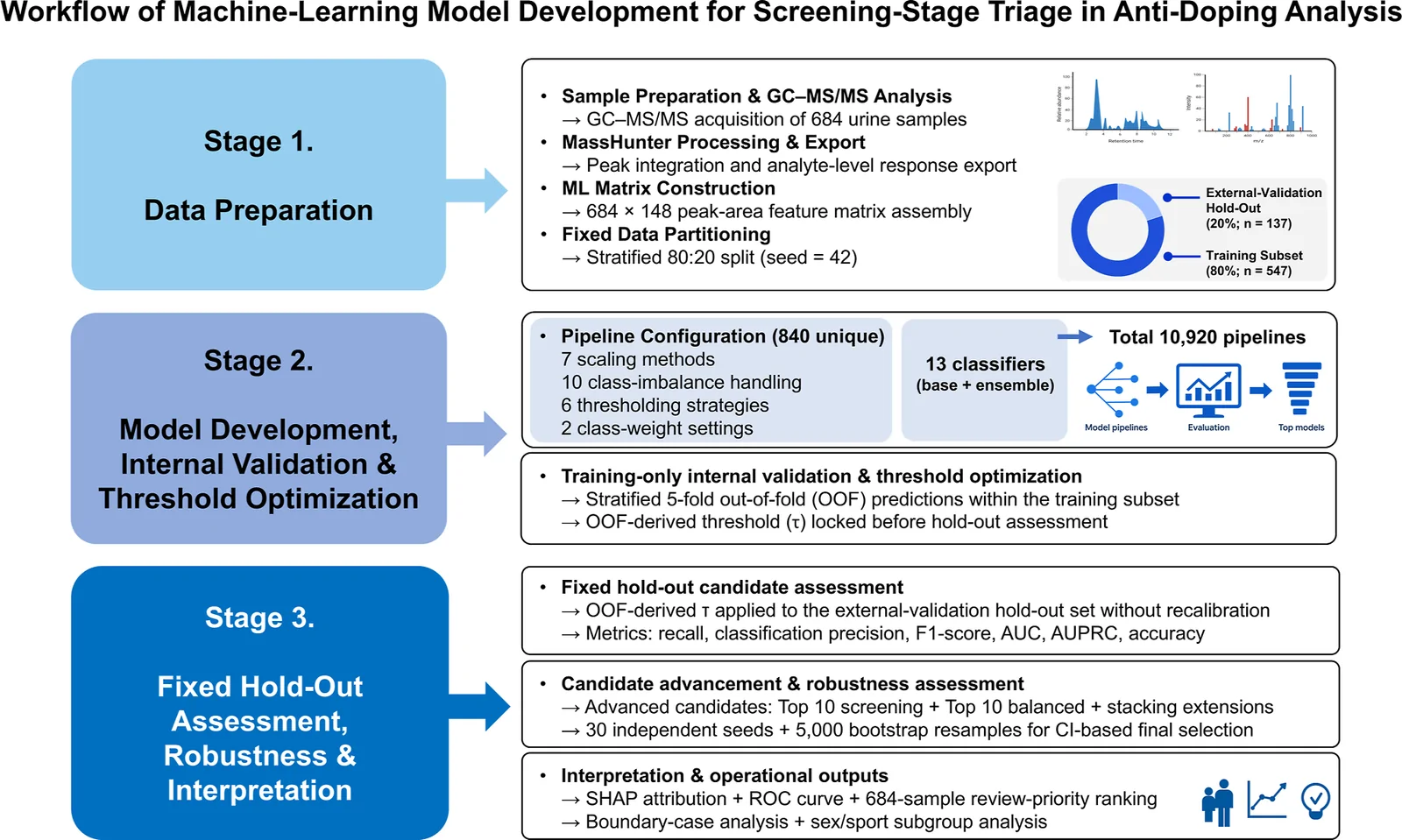
Workflow of machine-learning model development for screening-stage triage in antidoping analysis. The workflow comprises data preparation and fixed partitioning, model development with training-only internal validation and threshold optimization, and fixed external-validation hold-out assessment with robustness evaluation and interpretation. Stage 1 converts GC–MS/MS data into a MassHunter-derived 684 × 148 peak-area feature matrix and applies a fixed stratified 80:20 split. In Stage 2, 840 pipeline configurations and 13 classifier architectures generate 10,920 candidate pipelines. Within the training subset, stratified 5-fold out-of-fold (OOF) predictions are used for internal validation and threshold optimization, and the OOF-derived operating threshold (τ) is locked before any hold-out assessment. In Stage 3, the locked threshold is applied to the external-validation hold-out subset without recalibration, followed by candidate advancement, robustness assessment across 30 independent seeds with 5000 class-stratified bootstrap resamples per seed, and comprehensive interpretation, including SHAP feature attribution, ROC analysis, 684-sample review-priority ranking, and boundary-case/subgroup evaluations.

### Sample Preparation and Extraction

Urine samples (2 mL each) collected from athletes were aliquoted into labeled 15 mL glass tubes. Aliquots of positive control urine (PCU), negative control urine (NCU), and quality control samples were similarly prepared. 20 μL of an internal standard (ISTD) mixture and 1 mL of sodium phosphate buffer (pH 7.2) were added to each sample. After vortexing, 50 μL of β-glucuronidase (140 U/mL) was added to each sample for hydrolysis at 55 °C for 1 h. Subsequently, 20 μL of a 10 μg/mL methyltestosterone solution was added, and the pH was adjusted to 9.6 with 0.7 mL of 5% K_2_CO_3_. 5 mL of methyl *tert*-butyl ether was introduced for extraction. The organic layer was separated, evaporated under nitrogen, and vacuum-dried to remove residual moisture. Solid-phase extraction was then performed through PAD-1 resin packed into a Pasteur pipet, with methanol as the elution solvent. The eluate was evaporated to dryness via rotary evaporation under nitrogen. The samples were derivatized by adding 50 μL of *N*-methyl-*N*-trimethylsilyl-trifluoroacetamide (MSTFA)/ammonium iodide (NH_4_I)/dithioerythritol (DTE) (500:4:2) to the dried sample, followed by incubation at 60 °C for 20 min. The final derivatized samples were transferred into vials and directly analyzed via GC–MS/MS (Figure S1 in the Supporting Information).

### GC–MS/MS Instrumental Analysis

The GC–MS/MS analysis was performed using an Agilent 7890B GC system coupled with an Agilent 7010 triple quadrupole mass spectrometer (Agilent Technologies, Santa Clara, CA, USA). Chromatographic separation was achieved on an Agilent HP-Ultra 1 column (length: 17 m; internal diameter: 0.2 mm; film thickness: 0.11 μm; Agilent Technologies, Santa Clara, CA, USA). 1 μL of the sample was injected in split mode (10:1) using helium as the carrier gas flowing at 0.7 mL/min. The inlet temperature was set to 280 °C. Starting at 180 °C, the oven temperature increased to 200 °C at 20 °C/min, then to 210 °C at 5 °C/min and held for 4 min, then increased to 220 °C at 5 °C/min and held for 2.5 min, and finally ramped to 320 °C at 50 °C/min and held for 2.5 min. The total runtime of each analysis was 16 min. MS was conducted using electron ionization in the multiple reaction monitoring (MRM) mode. Table S1 in the Supporting Information lists the 148 prohibited substances monitored in this study, their corresponding MRM values, and their RTs. The data were acquired after a solvent delay of 2 min. The ion-source, quadrupole, and interface temperatures were maintained at 250 °C, 150 °C, and 300 °C, respectively. The flow rates of the quench gas (helium) and collision gas (nitrogen) were set to 2.25 and 1.5 mL/min, respectively. The analysis conditions are detailed in Table S2 of the Supporting Information.

### Manual Data Review

During manual review, analytical signals from monitored analytes were examined by comparing retention times (RTs), ion ratios, peak areas, and MRM transitions of each sample to those of the positive control urine (PCU). This process reflects the routine screening-level interpretation step in antidoping laboratories, in which suspicious analytical signals or atypical profiles are identified prior to compound-specific confirmation. In WADA-aligned laboratory practice, the presence of a prohibited substance may be represented by the parent analyte and/or its metabolite(s) and/or marker(s), and multiple metabolites or markers may collectively support the presence of a single prohibited substance; in addition, some substances are evaluated under threshold-based decision criteria, whereas others are primarily interpreted through qualitative identification and pattern consistency.^20,21^

Chromatographic consistency was assessed by comparing the RT or relative RT (RRT) of each analyte with the corresponding value of PCU; values within predefined acceptance windows (RT: ±1% or ±0.1 min; RRT: ±1%) were considered consistent with the reference standard, thereby minimizing false identifications.

The WADA-aligned screening review, confirmatory follow-up, and final reporting workflow are summarized in Figure S2, while representative chromatographic review scenarios illustrating the analytical complexity of manual interpretation are presented in Figure S3; both figures are provided in the Supporting Information.

Manual review requires expert interpretation of chromatographic data and is labor-intensive and time-consuming, particularly when multiple analytes, metabolites, or markers must be considered together. Interpretation of endogenous substances may be further influenced by physiological variation and baseline levels. These challenges support the AI-assisted triage of screening results. In routine practice, samples exhibiting suspicious analytical profiles are prioritized for confirmatory analysis, after which final positive or negative reporting outcomes are determined in accordance with applicable antidoping guidelines and expert review procedures.

### Data Preprocessing and Feature Extraction

The data set for machine-learning analysis was based on GC–MS/MS analytical data generated from authentic athlete urine samples. The resulting raw data were processed using Agilent MassHunter Quantitative Analysis (version 10.1, Agilent Technologies) with a predefined targeted screening-stage batch-processing method incorporating fixed MRM transitions, analyte-specific retention-time information, and consistent integration parameters for each analyte. Following data acquisition, target peaks were automatically integrated using the Agile2 integrator, and the resulting analytical tables were exported in batch mode. The exported response values were based on peak area rather than peak height. Key Agile2 settings included data point sampling = 1, smoothing = false, detection filtering = FivePoint, start threshold = 0.2, stop threshold = 0, peak location = Top, baseline reset = 5, baseline valley = 100, and baseline preference = Tangent; dynamic background subtraction was disabled. Analyte-specific retention-time tolerances were applied in minutes, with left retention-time deltas ranging from 0.045 to 0.400 min and right retention-time deltas ranging from 0.100 to 0.300 min. Automatically detected peaks were selected using the CloseRT criterion with a peak-area percentage threshold of 5%. User intervention was limited to routine review of the exported results, such as checking chromatographic alignment and verifying that the automated peak integration was reasonable. No manual peak-by-peak reintegration was performed. Detailed preprocessing and integration settings are summarized in Table S3 of the Supporting Information. After export, the resulting data were arranged into a consistent sample-by-analyte matrix for machine-learning analysis by removing duplicate entries, consolidating reanalysis results, and aligning analyte columns across injections. The final data set contained 684 samples: 628 negative (91.8%) and 56 positive (8.2%) cases. The final predictor matrix (X) used for machine learning consisted of 684 rows and 148 analyte-level features, each representing an integrated peak-area response value for the primary MRM transition of one monitored GC–MS/MS target analyte. Thus, X was a 684 × 148 sample-by-analyte response matrix of targeted peak-area responses, rather than raw GC–MS/MS chromatograms, untargeted peak features, or nonspecific peak-area signals.

Retention time deviation, ion ratio deviation, peak shape descriptors, signal-to-noise ratios, and integration quality information were not exported or supplied to the downstream machine-learning algorithms as independent predictor variables.

Nonzero exported peak-area response values were retained as continuous numerical feature values, including small nonzero responses. No additional postexport cutoff was applied to convert small response values to zero. When no peak-area response value was exported for a given sample–analyte combination, the corresponding entry was coded as 0 during postexport matrix alignment to maintain a complete 684 × 148 sample-by-analyte matrix. Thus, zero values represent the operational absence of an exported analyte-level response under the targeted processing conditions. Such entries may reflect no assigned target peak or a signal not distinguished from baseline/noise by the targeted processing method, but they should not be interpreted as definitive biological absence, manual peak rejection, or an imputed concentration value. The final predictor matrix did not encode separate flags for integration failure or peak rejection. No statistical imputation or missing-value estimation was applied. In the final matrix, exact zero values accounted for 8.78% of all sample-by-analyte entries, whereas 91.22% contained nonzero exported response values.

A separate binary sample-level reference label vector (*y*) was used for model development and evaluation with negative and positive cases encoded as 0 and 1, respectively. All samples labeled as positive in this study correspond to authentic urine specimens previously classified as adverse analytical findings (AAF) through established WADA-accredited confirmatory procedures. The confirmatory determinations used for reference-label assignment followed regulatory criteria, including retention-time matching, ion-ratio verification, and, where applicable, decision-limit evaluation according to applicable antidoping technical documents. Therefore, the positive class labels used for model development and evaluation reflect authoritative confirmatory determinations rather than exploratory or self-assigned classifications. Normalized intensity distribution plots for the 148 analyte-specific input features are provided in Figure S4 in the Supporting Information.

In addition to these numerical features, the data set included metadata such as confirmed detected substance(s), sport discipline, and athlete sex. To prevent information leakage, confirmed substance annotations were strictly excluded from the model feature set and were used only for postanalysis characterization of the model’s interpretation, including quantification of detection frequency (coverage) across analytes. A nonidentifiable summary of data set composition, cohort partitioning, and analyte diversity among confirmed-positive samples is provided in Table S4 in the Supporting Information.

### Design and Validation Strategy of the Chemometric Study

A multitier validation framework was implemented to reduce bias in model development and to provide rigorous assessment of generalization reliability. Using a fixed random seed (seed = 42), the complete data set (*n* = 684) was partitioned once into a training subset (80%) and a fixed held-out external-validation subset (20%) drawn from data generated within the same laboratory workflow. This subset was never used for model training, threshold optimization, preprocessing fitting, or feature selection. Instead, it served as a validation subset for comparative robustness assessment of prespecified candidate models, followed by post hoc interpretability analysis of the selected models. Within the training subset, the proportions of negative and positive samples were preserved across folds through stratified 5-fold cross-validation.^22^ The model-development grid comprised 840 configurations across four design dimensions—data scaling, class-imbalance handling, model-level class weighting, and decision-threshold strategy—evaluated across 13 classifier architectures, yielding 10,920 (840 × 13) model outputs evaluated through stratified cross-validation. Considering the analytical priorities in antidoping analytical workflows, we defined two complementary candidate-selection frameworks: (i) a screening-oriented framework that prioritized candidates achieving high mean classification recall during internal cross-validation, thereby minimizing false negatives during early stage triage, and (ii) a balanced framework based on Fβ or precision-floor optimization that prioritized the classification precision–recall trade-off. The top 10 configurations were selected based on framework-specific mean cross-validation performance (mean recall for screening; mean F1 for balance).

Although stacking architectures were included among the 13 classifiers evaluated through internal cross-validation, none of the stacking pipelines ranked within the top 10 configurations in either framework. To systematically evaluate their potential for robustness enhancement, stacking-based ensemble variants were newly generated from the same top-ranked configurations and included in the external validation, yielding 20 evaluated models per framework (10 original top configurations +10 stacking extensions). An auxiliary forced-recall evaluation (recall-floor ≥0.995) was additionally applied to the same screening source configurations and their corresponding stacking extensions. In total, 60 models across the screening, balanced, and auxiliary forced-recall evaluation sets were assessed under identical external-validation conditions, enabling systematic comparison of robustness across evaluation sets and models. The performance metrics and uncertainty quantification are described in the Statistical Performance Evaluation and Uncertainty Quantification section.

### Model Optimization and Hyperparameter Tuning

The data preprocessing and model tuning components were combined to generate 840 pipeline configurations (7 scaling methods × 10 resampling options × 2 class-weight modes × 6 threshold-selection variants).

#### Scaling Methods

Seven feature scaling strategies were evaluated: None, RobustScaler, StandardScaler, MinMaxScaler, Quantile transformation (uniform and normal output), and Yeo–Johnson power transformation.

#### Class-Imbalance Handling

The rarity of positive samples was addressed using 10 resampling strategies: (i) no resampling; (ii) random undersampling (RUS) at minority-to-majority ratios of 0.1, 0.2, 0.333, 0.5, 0.7, and 1.0; and (iii) synthetic oversampling using the synthetic minority oversampling technique (SMOTE) and its cleaned variants, SMOTE–Tomek and SMOTE–edited nearest neighbors (ENN).

#### Class Weighting

Two class weight modes (none and balanced) were evaluated. For algorithms supporting cost-sensitive learning [logistic regression, linear support vector machine (SVM), and random forest], the balanced mode used class weights that were inversely proportional to class frequencies. For XGBoost, the balanced mode used a positive-class scaling factor calculated from the negative-to-positive-class ratio in the corresponding training data.

#### Threshold-Selection Variants

Six threshold-selection variants—F-beta maximization (β = 0.5, 1.0, and 2.0), recall-floor criteria (recall ≥0.999 and ≥0.995), and a precision-floor criterion (classification precision, i.e., positive predictive value, ≥0.80)—were incorporated into the pipeline grid. The procedure used to determine the operating threshold (τ) from out-of-fold predictions is detailed in the Decision Threshold Optimization section.

#### Classifier Architectures

Thirteen classifier architectures—six base learners and seven ensemble variants—were evaluated within each pipeline. The base classifiers included logistic regression with an L1 penalty (saga solver),^23^ linear SVM^24^ with probabilistic calibration via Platt’s sigmoid function,^25^ Gaussian naïve Bayes,^26^ *k*-nearest neighbor (*k* = 5),^27^ random forest (≈600 trees),^28^ and XGBoost (≈800 trees; depth ≈5, learning rate ≈0.1, subsample ≈0.8).^29^ The ensemble variants consisted of hard voting (OR, AND, and majority voting with different thresholds), soft voting, and stacking with a logistic-regression meta-learner.

The resulting pipeline configurations were systematically benchmarked using cross-validated performance, and candidate advancement was based on the framework-specific selection criteria described below. The complete grid-search space, classifier configurations, and parameter settings are summarized in Table S5 of the Supporting Information. These classifier architectures were not selected as individually novel algorithms. Rather, they were chosen to represent complementary modeling assumptions across linear, probabilistic, distance-based, tree-based, boosting, and ensemble-type decision structures. The present study systematically benchmarked both individual base learners and ensemble-based extensions for the same antidoping screening task, rather than prespecifying a single preferred model.

### Decision Threshold Optimization

The operating threshold (τ), which converts probabilistic outputs into binary classifications, was optimized using out-of-fold (OOF) predictions obtained from stratified 5-fold cross-validation on the training set, ensuring that the external-validation set was not used for threshold selection. Three threshold-selection strategies were evaluated in the Stage 2 pipeline grid.

- F-β maximization (β = 0.5, 1.0, 2.0)
- Recall-floor optimization, evaluated at recall floors of 0.999 and 0.995, which selects the highest-precision τ satisfying the specified recall floor
- Precision-floor optimization, which selects the highest-recall τ satisfying classification precision ≥0.80

For Stage 2 candidate advancement, screening-oriented candidates were ranked primarily by mean cross-validation recall, whereas balanced candidates were ranked primarily by mean F1 performance. During Stage 3 external robustness evaluation, an auxiliary forced-recall condition (recall ≥0.995) was applied to the same screening source configurations, including their corresponding stacking extensions, without introducing new source configurations. For each candidate and each repetition, all applicable decision thresholds were determined solely from OOF predictions on the training subset. Where a single operating threshold τ was applicable, it was then fixed and applied once to the fixed held-out external-validation set without recalibration or threshold tuning on external data.

### Statistical Performance Evaluation and Uncertainty Quantification

Performance was assessed using the threshold-dependent metrics—classification precision (positive predictive value), recall, F1-score, and accuracy—computed at fixed τ, and the threshold-independent diagnostics—area under the receiver operating characteristic curve (ROC–AUC) and area under the precision–recall curve (AUPRC)—computed from the probabilistic outputs.

Reliability was quantified through a two-level uncertainty procedure combining repeated training with bootstrap resampling. Each model underwent 30 independent repetitions (*N* = 30) using a fixed sequence of random seeds (integers 42–71) to capture its stochastic optimization variability. Within each repetition, predictions on the fixed external-validation hold-out set (*n* = 137) were subjected to class-stratified nonparametric bootstrap resampling (*B* = 5000) to estimate finite-sample uncertainty.

For each metric (accuracy, recall, precision, F_1_, ROC–AUC, and AUPRC), a point estimate was recorded for each of the 30 repetitions. The overall performance estimate was calculated as the mean across the 30 repetitions, and the 95% seed CI was defined by the 2.5th and 97.5th percentiles of the corresponding seed-specific estimates. Within each repetition, a seed-specific 95% percentile-based bootstrap CI was obtained from the 5000 bootstrap resamples. The reported bootstrap interval was then summarized using the median lower and upper bounds of the seed-specific bootstrap CIs across the 30 repetitions. Final performance values are reported as mean estimates together with 95% seed CIs and bootstrap interval summaries, reflecting between-seed training variability and finite-sample variability within the fixed external-validation set, respectively.

### Chemical Interpretability and Feature Attribution

The contributions of individual analyte-specific features to model predictions were assessed using Shapley additive exPlanations (SHAP). Because all interpretability analyses were performed exclusively on the fixed external test set (*n* = 137), the resulting feature attribution profiles reflect model generalization behavior rather than patterns derived solely from the training data.

The top-ranked classifiers included heterogeneous ensemble architectures (e.g., stacking and soft voting), which could not be interpreted under a single-tree-specific attribution framework. Therefore, feature attribution was performed by using KernelSHAP, treating each model as a probabilistic classifier. SHAP values were estimated relative to a background reference set of 200 randomly selected training samples to approximate the data distribution encountered during the model development.

Global feature importance was defined as the mean absolute SHAP value aggregated across all external-test samples. Because absolute SHAP magnitudes are not directly comparable across heterogeneous ensemble architectures, within-model importance scores were normalized to a 0–1 scale before cross-model comparison. In parallel, feature coverage, defined as the fraction of external-test samples with nonzero analyte intensity, was calculated to contextualize feature importance in relation to empirical occurrence patterns. SHAP analyses were conducted independently for each of the five statistically robust pipelines. In addition to global feature-importance summaries, sample-level SHAP attribution was examined for representative external-test samples to visualize the local analyte contributions driving individual model predictions.^30,31^

### Confounder and Subgroup Analysis

To evaluate whether the metadata-related factors influenced the model outputs, athlete sex and sport discipline were incorporated into a structured confounder and subgroup analysis based on the fixed external test set (*n* = 137). For each sex group and recorded sport, subgroup-level predictive performance was computed using the model-generated positive-class probabilities together with the operating threshold τ determined during model optimization. This threshold was fixed prior to subgroup analysis and applied uniformly across all subgroups, ensuring fair comparability without introducing subgroup-specific recalibration or information leakage.

To determine whether the metadata variables were independently associated with the ground-truth doping outcomes, complementary statistical procedures were applied to athlete sex and sport discipline according to the available subgroup structure. For athlete sex, a covariate-adjusted logistic regression model was fitted using the logit-transformed prediction score as the primary explanatory variable and sex as a binary covariate (setting “female” as the reference). Statistical significance was assessed using Wald tests with a two-sided threshold of *p* < 0.05. For sport discipline, formal hypothesis testing was precluded by complete- and quasi-separation effects in the preliminary models caused by the severe sparsity and single-class dominance within sport categories. Instead, an exploratory L2-regularized logistic regression was fitted to the 15 most represented sport disciplines using athlete sex and one-hot encoded sport categories as predictors of ground-truth doping status. One sport discipline served as the reference category, and the resulting sport coefficients were interpreted descriptively for magnitude and directional pattern, complemented with descriptive subgroup performance statistics for each sport category.

### Software and Computing Environment

All chemometric analyses and model development were performed in the Python programming language (version 3.13.5). The computational workflow relied on the following core libraries: NumPy (v2.3.3) and pandas (v2.3.3) for data curation; scikit-learn (v1.7.2) and imbalanced-learn (v0.14.0) for pipeline construction; and XGBoost (v3.0.5) for gradient boosting implementation. SHAP (v0.48.0) and Matplotlib (v3.10.6) were employed for feature attribution and visualization, respectively. All experiments were executed on a Windows 11 (AMD64) computing platform. To support reproducibility, an exact environment snapshot detailing all package versions and dependencies has been archived with the project data set.

## Results and Discussion

### Internal Validation and Model Selection

During the internal validation phase, 10,920 chemometric pipelines were systematically benchmarked using stratified 5-fold cross-validation. The evaluation results of all 10,920 pipeline configurations are summarized in Table S6 of the Supporting Information.

To evaluate the classification precision–recall trade-off, the top 10 pipelines were selected from each framework. The performance metrics and specific configurations of these top-ranked models are summarized in Table S7 of the Supporting Information. Under the screening-oriented configuration (prioritizing recall), the top models achieved very high classification recall, yielding a mean recall of 1.000 ± 0.000 and a ROC–AUC of 0.987 ± 0.005. Although this prioritization reduced the precision to a moderate 0.706 ± 0.024, the resulting recall profile was consistent with the screening priority of minimizing false negatives. In contrast, the balanced configuration maintained a robust equilibrium with a mean F1-score of 0.842 ± 0.005, a precision of 0.847 ± 0.132, and a recall of 0.873 ± 0.130.

Among the evaluated architectures, the MajorityVote (*k* = 3) ensemble with random undersampling (ratio = 0.1) and quantile normalization yielded the highest single-model performance in the internal phase (Table S7 in the Supporting Information). Although these internal metrics highlight promising candidate architectures, cross-validation alone can be overly optimistic because it does not fully account for stochastic optimization variability or finite-sample uncertainty. Therefore, to assess analytical reliability beyond internal cross-validation, the leading candidate models were subsequently carried forward to external robustness analysis integrating repeated-seed retraining and bootstrap-based uncertainty quantification. The significance of this large-scale comparison lies not in any single classifier itself, but in establishing a structured chemometric selection framework for identifying which combinations of preprocessing, classifier architecture, ensemble design, and decision objective are most suitable for antidoping screening. In this respect, the present work extends the antidoping ML literature beyond broader review-level discussions and other analytical settings by systematically benchmarking 10,920 candidate pipelines on authentic human urine GC–MS/MS screening data defined by confirmatory outcomes.^20,21^

### External Validation and Robustness Assessment

External validation evaluated the generalization performance and statistical robustness of the candidate models advanced from the internal cross-validation phase. Sixty pipelines were examined across the screening-oriented and balanced frameworks together with an auxiliary forced-recall evaluation. The screening and balanced frameworks each included 10 top-ranked configurations and 10 corresponding stacking-based extensions, yielding 20 candidates per framework. The auxiliary forced-recall evaluation applied the recall-floor ≥0.995 condition to the same 10 screening source configurations and their corresponding stacking extensions, yielding an additional 20 candidates.

To strictly isolate the development and validation stages, the models were trained on the fixed training subset and evaluated exclusively on the fixed external-validation hold-out set (*n* = 137) using operating thresholds (τ) determined from out-of-fold predictions within the training data and fixed before external testing. Each pipeline underwent 30 independent repetitions using distinct random seeds. In each repetition, a complete retraining–testing cycle was performed, and the probabilistic outputs were further evaluated via class-stratified nonparametric bootstrap resampling (*B* = 5000) of the fixed external-validation predictions to quantify the finite-sample uncertainty. This two-tier procedure captured both the between-seed stochastic variation and the within-seed sampling variability. All results were summarized as mean performance estimates with 95% seed and bootstrap CIs; the reported bootstrap interval was summarized using the median lower and upper bounds of the seed-specific percentile bootstrap CIs across the 30 repetitions.

The robustness analysis revealed distinct stability patterns across the configurations and individual models. In the screening configuration, only a limited subset of the MajorityVote pipelines sustained consistently high recall; several stacking-based variants exhibited pronounced variability with wide recall CIs (up to 0.45), indicating limited robustness among some high-recall screening-oriented configurations under severe class imbalance. In contrast, many of the balanced-configuration pipelines demonstrated narrow recall and F_1_ CIs (≤0.27), reflecting reproducible precision–recall behavior across stochastic repetitions. The forced-recall variants, designed to enforce recall values of 0.995 or higher, showed pervasive instability and F_1_ values that were frequently below practically acceptable thresholds, indicating that excessively strict recall enforcement may compromise overall reliability.

Stacking generally enhanced robustness only within the balanced framework, where the selected models showed reduced recall variability and more stable AUPRC behavior across repeated seed and bootstrap analyses. In the screening configuration, stacking often amplified rather than suppressed variability. These findings highlight the value of CI-based robustness assessment beyond cross-validation alone, particularly for revealing instability across multiple rounds of retraining and resampling. The comprehensive seed-level performance metrics and bootstrap-derived CIs for all 60 pipelines are reported in Table S8 of the Supporting Information. These results also provided an empirical basis for the exclusion criteria described below.

Collectively, the screening-oriented and balanced frameworks, together with the auxiliary forced-recall evaluation, showed complementary operating characteristics: the screening configuration probed recall-maximized yet variance-prone designs; the balanced configuration identified more reproducible precision–recall behavior; and the auxiliary forced-recall evaluation delineated the stability limits of stringent recall-driven optimization.

### Robustness Diagnostics for Failure Mode Identification

External evaluation of the 60 pipelines was followed by a detailed inspection of the seed-level variability and bootstrap-derived CIs (Table S8 in the Supporting Information). Three characteristic failure patterns emerged, which informed the exclusion of unstable models.

First, the forced-recall pipelines (recall ≥0.995) consistently demonstrated poor threshold-dependent classification performance. The precision and F_1_ scores were severely degraded (F_1_ < 0.450) across seeds and bootstrap resamples, indicating that enforcing an extreme recall constraint can severely compromise precision and overall classification balance.

Second, several stacking-based screening pipelines failed to maintain even a minimal acceptable recall (≥0.80). In some configurations, mean recall values ranged from 0.288 to 0.373, and the 95% bootstrap recall CIs were exceedingly broad, spanning ranges such as 0.000–0.545 or 0.091–0.636. These patterns demonstrate insufficient recall on the external hold-out set and show that several stacking configurations within the screening framework failed to meet the minimum recall requirement for screening-stage triage.

Third, even the highest-ranked MajorityVote (*k* = 3) pipeline under the screening framework—configured with random undersampling (ratio = 0.1) and quantile normalization—exhibited pronounced instability under robustness analysis. Although this model achieved strong internal cross-validation performance (mean precision = 0.733, recall = 1.000, and F_1_ = 0.846), its external recall fluctuated widely in the bootstrap evaluation. The 95% bootstrap recall CI ranged from 0.182 to 0.727, indicating substantial uncertainty in external recall and a marked loss of stability relative to the internal cross-validation estimate. Such variability rendered this configuration insufficiently reliable for prioritization as a primary screening candidate despite its favorable internal performance. Taken together, these three failure modes provided the empirical basis for the exclusion criteria applied in the subsequent robust-model selection procedure.

### Identification and Characterization of Robust Analytical Pipelines

On the basis of the failure modes identified in the robustness diagnostics, we selected a small set of pipelines combining high classification recall with stable performance across random seeds and bootstrap resamples, thereby reducing reliance on favorable but potentially fragile single-point estimates.

To this end, the models were selected through a three-step filtering procedure of the externally validated pipelines: (i) baseline screening for recall and overall performance; (ii) exclusion of models with unstable confidence intervals; and (iii) ranking by integrated robustness criteria. Step (i) excluded pipelines that violated the minimum screening requirements (recall mean of <0.80 or F1 mean of <0.60 on the external set). Even if statistically stable, such configurations are unsuitable for routine antidoping screening because they either show insufficient recall or return an excessive number of false positives. Step (ii) removed the surviving pipelines showing excessively wide recall or F1 CIs across the seed-level and bootstrap analyses, indicating poor reproducibility under repeated training or resampling of the test instances. The exclusion thresholds were informed by the instability patterns documented in the robustness diagnostics, and only pipelines with consistently narrow CIs across seeds and resamples were retained for final ranking. In step (iii), the surviving pipelines were ranked by prioritizing (a) low recall CI width, (b) high mean F1-score, and (c) low F1 CI width. Marginal differences in average accuracy or AUC were ignored in the ranking, reflecting the analytical emphasis on reproducibility over nominal performance inflation. This filtering procedure ultimately selected the five most robust pipelines among all externally validated models (Table 1). To provide an intuitive illustration of how these robustness characteristics translate into concrete classification outcomes, confusion matrices for the five selected pipelines are shown in Figure S5 of the Supporting Information, based on a representative external evaluation (seed = 42).

**Table 1:** Performance Comparison of the Top Five Optimized Machine-Learning Pipelines for Doping-Control Classification

rank	model and configuration^a^	recall^b^	precision^b^	F1^b^	accuracy^b^	AUC^b^	AUPRC^b^
1	stacking; balanced; SMOTE–TOMEK; balanced; Robust; β = 2; τ = 0.374	0.976 (0.818–1.000; 1.000–1.000)	0.547 (0.440–0.724; 0.415–0.733)	0.696 (0.611–0.787; 0.579–0.846)	0.930 (0.898–0.964; 0.887–0.971)	0.986 (0.979–0.991; 0.967–0.999)	0.868 (0.813–0.907; 0.721–0.986)
2	stacking; balanced; SMOTE–TOMEK; none; Robust; β = 2; τ = 0.357	0.955 (0.818–1.000; 1.000–1.000)	0.542 (0.440–0.714; 0.390–0.710)	0.684 (0.611–0.787; 0.538–0.831)	0.927 (0.898–0.960; 0.876–0.967)	0.985 (0.977–0.989; 0.964–0.999)	0.864 (0.818–0.894; 0.718–0.985)
3	soft-voting; balanced; SMOTE; none; Robust; β = 1; τ = 0.352	0.839 (0.636–1.000; 0.727–1.000)	0.698 (0.517–0.875; 0.500–0.958)	0.750 (0.667–0.837; 0.594–0.917)	0.955 (0.925–0.971; 0.920–0.985)	0.987 (0.982–0.990; 0.967–0.999)	0.886 (0.855–0.903; 0.749–0.992)
4	stacking; balanced; SMOTE–TOMEK; balanced; Robust; β = 1; τ = 0.623	0.833 (0.546–1.000; 0.727–1.000)	0.681 (0.536–0.862; 0.500–0.909)	0.736 (0.641–0.809; 0.570–0.917)	0.952 (0.930–0.966; 0.920–0.985)	0.986 (0.979–0.991; 0.967–0.999)	0.868 (0.813–0.907; 0.721–0.986)
5	majority-vote (*k* = 3); screening; RUS-0.7; balanced; none; β = 1; τ = 0.489	0.852 (0.636–1.000; 0.727–1.000)	0.648 (0.513–0.764; 0.474–0.900)	0.729 (0.621–0.804; 0.561–0.909)	0.949 (0.923–0.964; 0.912–0.985)	0.974 (0.950–0.985; 0.949–0.995)	0.667 (0.553–0.737; 0.489–0.928)

a Model configuration denotes algorithm; optimization framework (screening or balanced); resampling strategy; class-weight setting; scaling method; optimization metric (beta); median decision threshold (τ). All thresholds were optimized via stratified 5-fold out-of-fold (OOF) cross-validation.

b Performance metrics are reported as mean (95% seed CI; bootstrap interval summary). Seed CIs represent the 2.5th–97.5th percentile range across 30 independent repetitions. Bootstrap intervals summarize the median lower and upper bounds of seed-specific 95% CIs obtained from 5000 class-stratified resamples per repetition. Precision denotes classification precision (positive predictive value), not analytical-method precision.

The two top-ranked pipelines (Ranks 1 and 2) are stacking ensembles selected from the balanced analytical framework. Both combined SMOTE–Tomek resampling with Robust scaling, while differing in class-weight setting. Consistent with the robustness patterns summarized in Figure 2, both models achieved near-perfect mean recall on the external data set, with the narrowest seed-level recall CIs among the five selected models and reported bootstrap recall intervals of 1.000–1.000. Both models also maintained mean F1 scores around 0.69 and high AUC/AUPRC values, while classification precision remained moderate, reflecting the trade-off associated with prioritizing high recall. The second robustness tier comprises the soft voting ensemble (Rank 3), a stacking model optimized using Fβ with β = 1, equivalent to F1-score optimization (Rank 4), and a majority-vote (*k* = 3) ensemble (Rank 5). These pipelines also satisfied the predefined recall and F1 criteria while maintaining comparatively narrow reported bootstrap recall intervals (width ≤0.273) but showed slightly broader dispersions than the top two stacking models (Figure 2). The configurations of the second-tier pipelines highlight that even relatively simple ensemble rules, such as majority vote, can achieve acceptable robustness when combined with appropriate resampling and class-weighting strategies.

**Figure 2:**
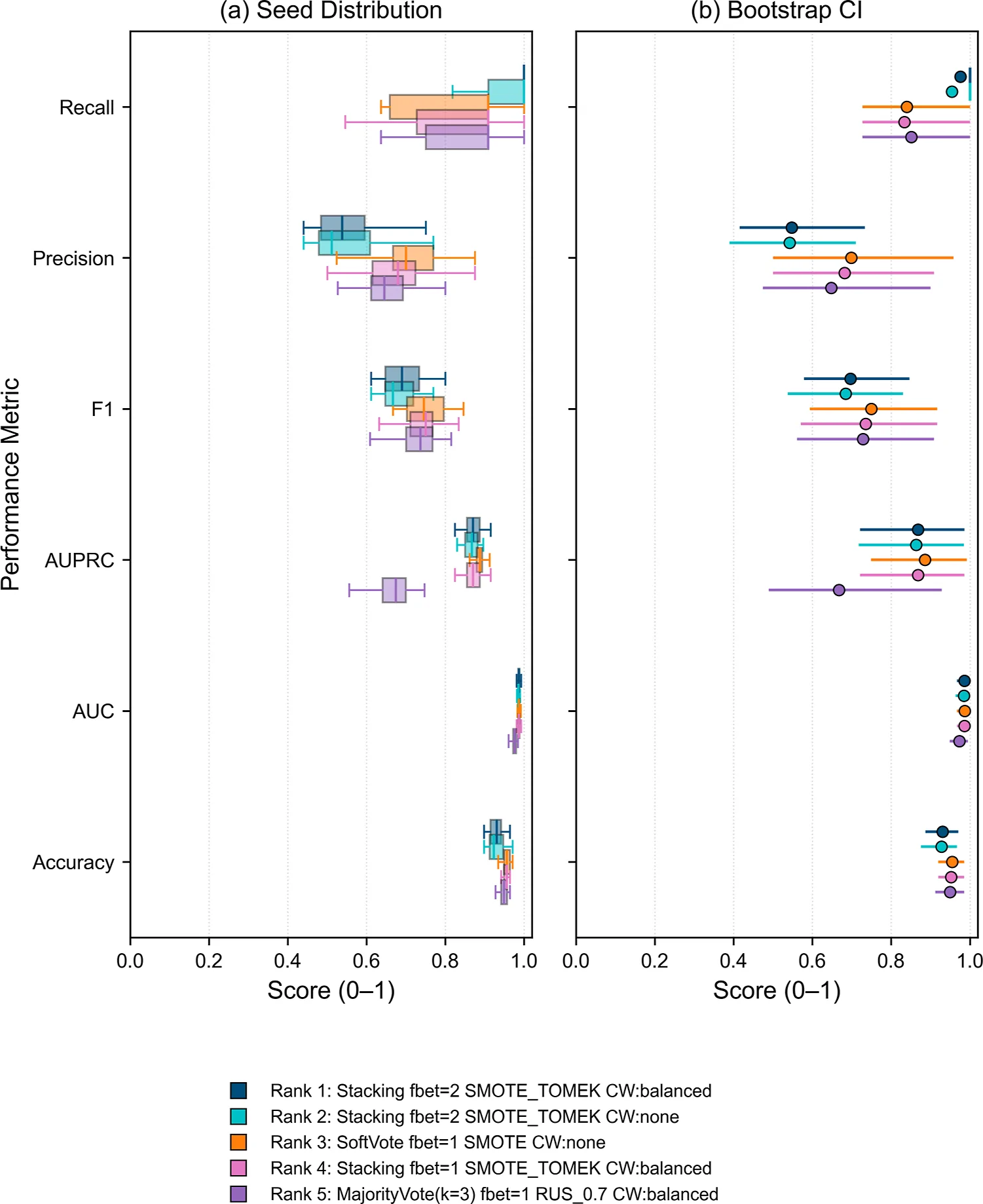
Model performance comparison based on seed distribution and bootstrap confidence intervals. (a) Prediction performance distributions across 30 random seeds for the top five robustness-ranked pipelines. Narrower and more centralized distributions indicate reduced dependence on random initialization and greater algorithmic stability. (b) Reported bootstrap intervals summarizing seed-specific 95% percentile-based bootstrap CIs obtained from 5000 class-stratified resamples per repetition. These intervals characterize finite-sample variability under resampling of the fixed external-validation set. Across the two analyses, the Rank 1 pipeline (Stacking, Fβ = 2, SMOTE–TOMEK, class-weight balanced) showed consistently high stability, with particularly narrow bootstrap intervals relative to the alternative ensemble configurations. The broader seed distributions and bootstrap intervals observed for some of the other models suggest greater sensitivity to stochastic retraining or test-sample resampling.

The stronger robustness of the top-ranked stacking architectures is consistent with their ability to combine complementary decision structures and reduce the variance across stochastic retraining. To illustrate why stability-based criteria were prioritized over mean-only performance estimates, Figure S6 in the Supporting Information compares the mean performance estimates of the same top five models. Whereas the bar plot displays broadly similar average scores across the ensembles, Figure 2 clarifies marked differences among the uncertainty structures, with some pipelines exhibiting much wider seed distributions and bootstrap intervals than others despite their comparable means. These results collectively support the use of seed- and bootstrap-based stability metrics for the identification of robust chemometric pipelines. They also support the selection of the optimal Rank 1 stacking model as a statistically reliable and analytically defensible candidate for screening-stage triage in routine antidoping operations.

As an additional characterization of the selected Rank 1 model, the ROC curve on the fixed external-validation hold-out set is provided in Figure S7 of the Supporting Information (ROC–AUC = 0.988), and a post hoc review-priority ranking of all 684 samples is provided in Figure S8 of the Supporting Information. The corresponding machine-readable all-sample ranking values are provided in Table S9 of the Supporting Information. The all-sample ranking was used only to visualize sample-prioritization behavior and was not used for model training, threshold optimization, model selection, or independent performance estimation.

A post hoc review of the model-flagged nonpositive samples provided additional insight into the prioritization behavior of the model. All 24 model-flagged nonpositive samples from the post hoc all-sample analysis are summarized in Table S10 of the Supporting Information. These samples were negative for the current GC–MS/MS end point used as the model reference label but exceeded the fixed operating threshold when the model was applied to all 684 samples. Because the model was trained only on the 148 GC–MS/MS screening-stage response features, information from other analytical workflows was not available to the model. Nevertheless, historical laboratory metadata showed that these model-flagged nonpositive samples had broader sample-level analytical context, primarily adverse analytical finding (AAF)-related outcomes in a separate LC–MS/MS workflow and, in fewer cases, AAF- or atypical finding (ATF)-related outcomes in separate hCG/LH/hGH isoform testing workflows. This observation suggests that the multivariate GC–MS/MS response pattern may help prioritize end point-negative samples with broader analytical relevance, supporting the utility of the proposed framework as a screening-triage and review-prioritization aid.

### In-Depth Interpretation of the Optimal (Rank 1) Model

Having established the statistical robustness of the top-ranked pipelines, we next examined the decision structure of the optimal rank 1 stacking model through SHAP-based interpretability analysis.

Before interpreting SHAP-based feature attribution, the normalized feature distributions in Figure S4 in the Supporting Information should be understood as exploratory visualizations of MassHunter-derived screening-stage response features. Because the class labels were assigned at the sample level, the positive class in an individual analyte panel may include samples confirmed to be positive for analytes other than the specific feature displayed in that panel. Therefore, partial overlap between negative- and positive-labeled samples in single-feature distributions does not indicate final compound-specific positive findings in negative samples or the analytical unreliability of the corresponding analyte feature. Rather, these patterns reflect the heterogeneous endogenous background, matrix-dependent responses, and nonreportable chromatographic signals that can be observed in authentic athlete urine samples, supporting the need for a multivariate framework that integrates information across all 148 response features. For steroid-related markers such as 19-NA, low-level endogenous or background responses require interpretation within established reporting and confirmatory criteria, whereas a numerical screening stage response assigned to the clenbuterol feature is not, by itself, equivalent to a final reportable clenbuterol finding.

Figure 3 presents SHAP-based interpretation of the optimal rank 1 model, with Figure 3a showing the SHAP beeswarm summary plot of global analyte importance and the magnitude and direction of per-sample contributions to the model output.

**Figure 3:**
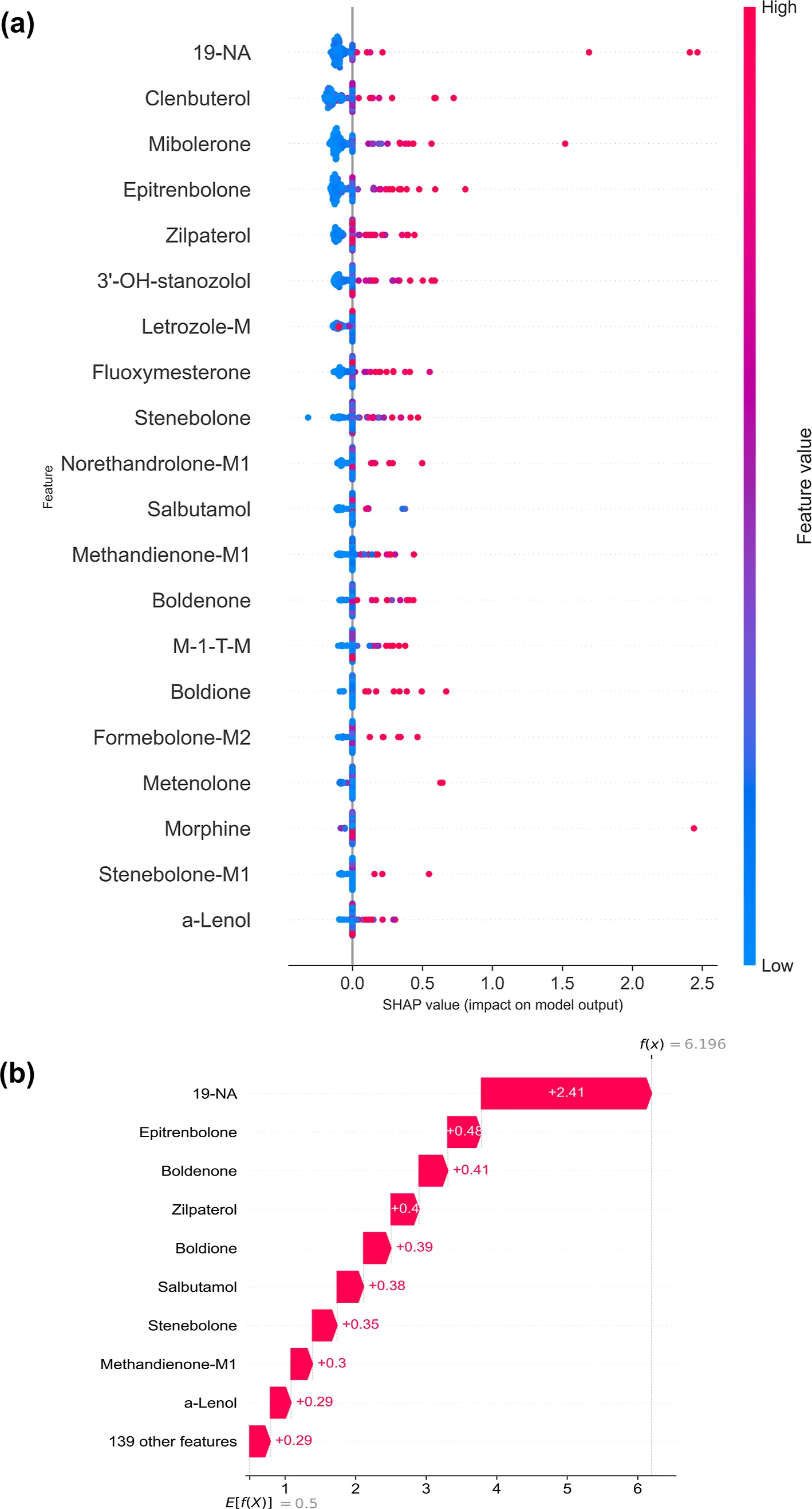
SHAP-based global and local interpretation of the optimal Rank 1 model. (a) SHAP beeswarm summary plot of the optimal Rank 1 model across the external test set. Each dot represents one sample, and the color gradient indicates the relative feature value of the corresponding analyte (primary MRM peak area), while the SHAP value represents its contribution to the model output within the full multivariate decision framework. Features are ordered by mean absolute SHAP value. High feature values of 19-norandrosterone (19-NA), clenbuterol, mibolerone, and epitrenbolone are predominantly associated with positive SHAP values, indicating that stronger analyte-specific signals increased the model output toward the positive class within the multivariate model context. (b) SHAP waterfall plot for a representative external-test sample confirmed positive for 19-NA. The plot shows how the contributions of the 148 analyte-level response features shifted the model output for this individual sample relative to the baseline expectation (*E*[*f*(*X*)] = 0.5) to the final model output (*f*(*x*) = 6.196). In this representative case, 19-NA was the dominant positive contributor, with additional positive contributions from several other analytes. Together, the two panels illustrate both the global feature-attribution structure of the model and the local analyte-level factors underlying an individual sample prediction.

In the global interpretation (Figure 3a), the underlying feature values (low to high analyte feature values, represented by primary MRM peak areas on the color scale) show how analyte-specific signal intensities were translated into differential contributions within the full multivariate decision framework. 19-Norandrosterone (19-NA) and clenbuterol emerged as dominant predictors. High feature values (points shown in red) of both analytes were predominantly associated with positive SHAP values, indicating that stronger analyte-specific signals consistently pushed the model output toward the positive class within the full-model context. Other highly ranked anabolic agents, including mibolerone and epitrenbolone, also showed strong positive contributions at high signal intensities. Together, these patterns indicate that the model learned a chemically and analytically plausible multivariate decision structure in which informative analyte-specific signals were prioritized over random analytical noise or noninformative variance.

To examine whether feature importance simply reflected feature coverage, we related the mean absolute SHAP values to the fraction of samples with nonzero responses for each analyte feature (Figure S9, Supporting Information). No monotonic relationship was observed: while relatively higher-coverage features such as 19-NA and clenbuterol exhibited high SHAP values, lower-coverage features such as zilpaterol and 3′-OH-stanozolol also showed substantial attribution, indicating that model importance was not determined solely by coverage.

The local interpretation further illustrated the practical value of analyte-level attribution (Figure 3b). In the representative external-test sample shown in Figure 3b, 19-NA was the dominant positive contributor to the model output. Consistent with this attribution pattern, the sample had been confirmed as a positive case on the basis of 19-NA. This representative example demonstrates that the model can provide chemically meaningful analyte prioritization cues in addition to a binary suspicious/nonsuspicious flag, with secondary contributions from other analytes reflecting the broader multivariate steroid profile captured by the model. In practical antidoping workflows, such information may help direct attention toward the most relevant analytical signals during follow-up review and thereby support more focused progression to confirmatory analysis. To further support case-level interpretability, representative annotated operational boundary cases are provided in Table S11 of the Supporting Information. These cases were selected to illustrate operationally relevant boundary scenarios, including a low-margin true-positive sample, a high-scoring true-negative sample retained below the operating threshold, and the top-ranked model-flagged nonpositive sample. The table provides both the case-level summary and the corresponding complete 148-analyte response profiles in separate worksheets, allowing transparent inspection of the screening stage model input context for each representative case.

### Comparative Interpretation of the Top 5 Models

To determine whether the selected robust pipelines reflected reproducible and chemically interpretable attribution patterns rather than architecture-specific artifacts, we compared the SHAP-based feature-importance profiles of the five statistically robust models. Figure 4 summarizes the relative importance of the top 20 analytes across all five pipelines. Because absolute SHAP magnitudes are not directly comparable across heterogeneous ensemble architectures, the within-model importance scores were normalized to a 0–1 scale to enable direct comparison of attribution patterns.

**Figure 4:**
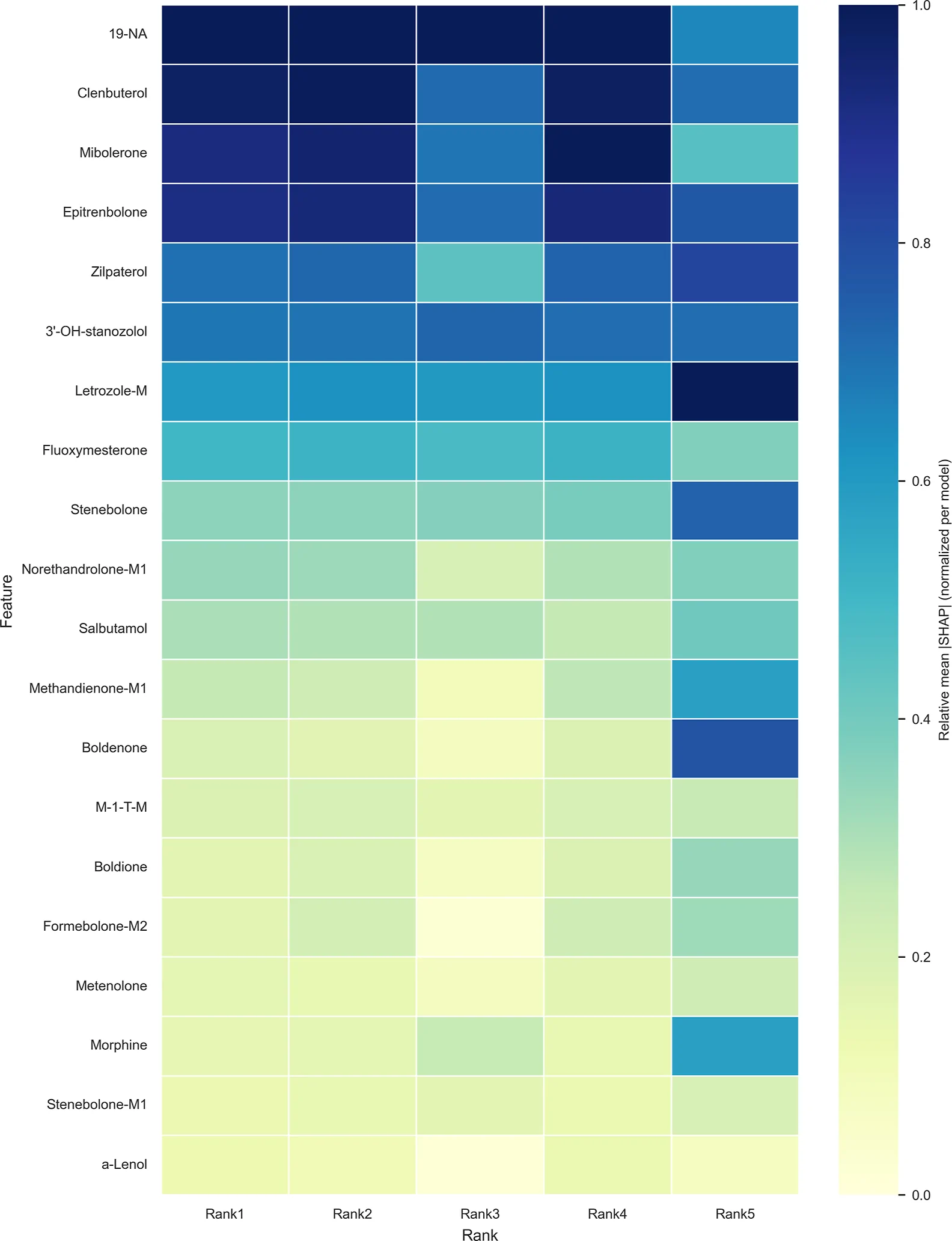
Comparative SHAP-based feature attribution across the five most robust pipelines. Heatmap showing normalized mean absolute SHAP importance of the top 20 analytes across the five statistically robust models (Ranks 1–5). Within each model, mean absolute SHAP values were normalized to a 0–1 scale to enable comparison of attribution patterns across different ensemble architectures. The four highest-ranked models (Ranks 1–4) display similar attribution profiles for key analytes, whereas the Rank 5 majority-vote model exhibits a redistributed attribution pattern.

A strong and coherent consensus was observed among the top-tier models (Ranks 1–4). Despite differences in classifier architecture (e.g., stacking versus soft voting), these models showed highly similar feature-importance patterns. Core high-contributing analytes—19-NA, clenbuterol, mibolerone, and epitrenbolone—consistently ranked among the most influential contributors across these models (Figure 4). This cross-model agreement indicates that the Rank 1 pipeline did not rely on idiosyncratic feature weighting specific to a single model configuration but instead captured a reproducible and chemically interpretable decision structure shared across multiple robust models.

By contrast, Rank 5 showed a redistributed feature-importance pattern, with reduced prominence of several dominant contributors and relatively greater emphasis on secondary features. When interpreted together with its comparatively lower robustness under repeated-seed and bootstrap evaluation (Table 1 and Figure 2), this shift indicates that the Rank 5 attribution pattern differed from the cross-model consensus observed among Ranks 1–4.

Taken together, the convergence of feature importance among Ranks 1–4 provides additional support for prioritizing the Rank 1 pipeline. Its selection was therefore justified not only by its statistical robustness, but also by the consistency of its feature-attribution logic with other high-performing models. This cross-model agreement strengthens the analytical defensibility of the selected model for screening-stage triage in antidoping workflows.

### Confounding Factors and Metadata-Related Variability

To evaluate whether metadata-related factors introduced systematic bias into the rank 1 model, we examined its prediction behavior across athlete sex and sport discipline on the fixed external test set (*n* = 137). Sex-stratified comparisons revealed largely overlapping probability distributions between male and female athletes (Figure 5). The violin plots showed closely overlapping density structures, similar quartile patterns, and comparable ranges of predicted positive class probabilities across the two groups. Accordingly, the model showed no evident systematic tendency to over- or underestimate the likelihood of a positive classification in either group. The different prevalences of observed positive cases (11.7% in females vs 5.2% in males) reflect the observed ground truth class distribution rather than a model-generated effect. Supporting this interpretation, the logit-transformed prediction score in a covariate-adjusted logistic regression was the only significant predictor of doping status, whereas the sex covariate was not significant (Wald test, *p* > 0.05). These findings indicate that, after accounting for the model-derived prediction score, sex was not independently associated with the ground-truth doping status.

**Figure 5:**
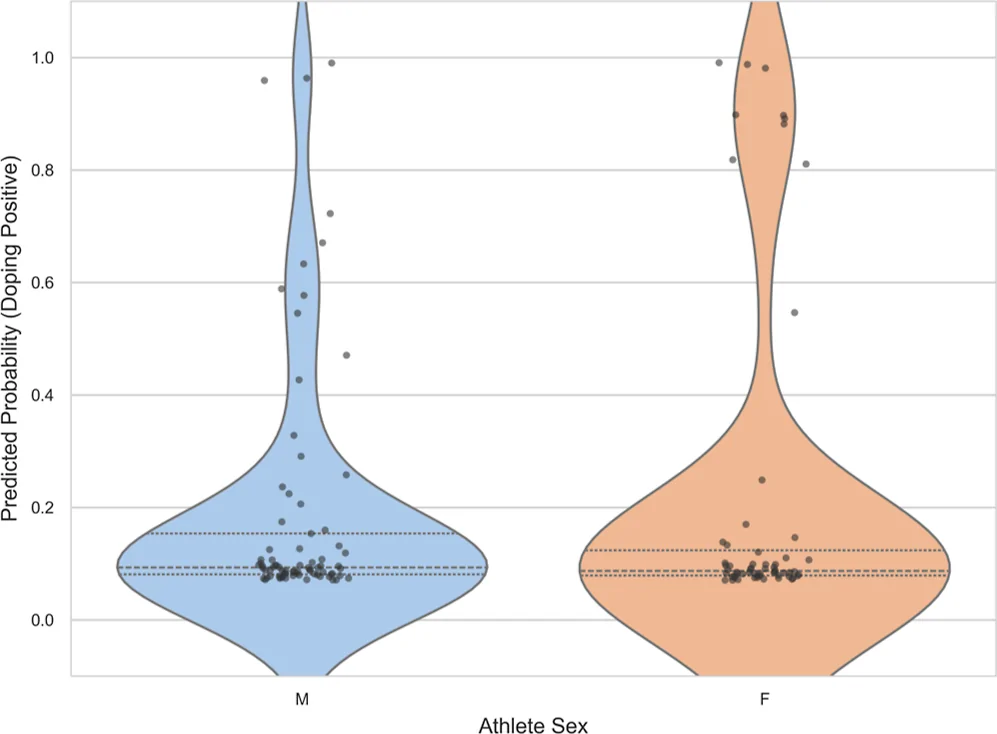
Distribution of model-predicted positive-class probabilities stratified by athlete sex. The violin plots show the score distributions for male (M) and female (F) athletes in the external test set (*n* = 137), with individual samples overlaid as points. Internal dashed lines indicate the quartiles of each distribution. The largely overlapping central distributions, with high-probability values occurring in both groups, indicate no evident systematic sex-dependent shift in model output.

Sport discipline exhibited substantially greater sparsity and imbalance than sex, with many categories represented by only 1–3 samples. Under such conditions, formal multivariable inference is unreliable because of the risk of complete or quasi-complete separation. Accordingly, an exploratory L2-regularized logistic regression was fitted to the 15 most represented sport disciplines, with athlete sex and one-hot encoded sport categories as predictors of ground-truth doping status. One sport discipline served as the reference category, leaving 14 sport coefficients displayed in Figure 6. The coefficients varied in direction around zero with no consistent directional pattern. Given the sparse subgroup sizes, these coefficients were interpreted descriptively rather than as formal inferential estimates. Together, these analyses did not reveal an evident systematic sex- or sport-related pattern in the external test set, while sport-specific inference remains limited by the sparse category sizes.

**Figure 6:**
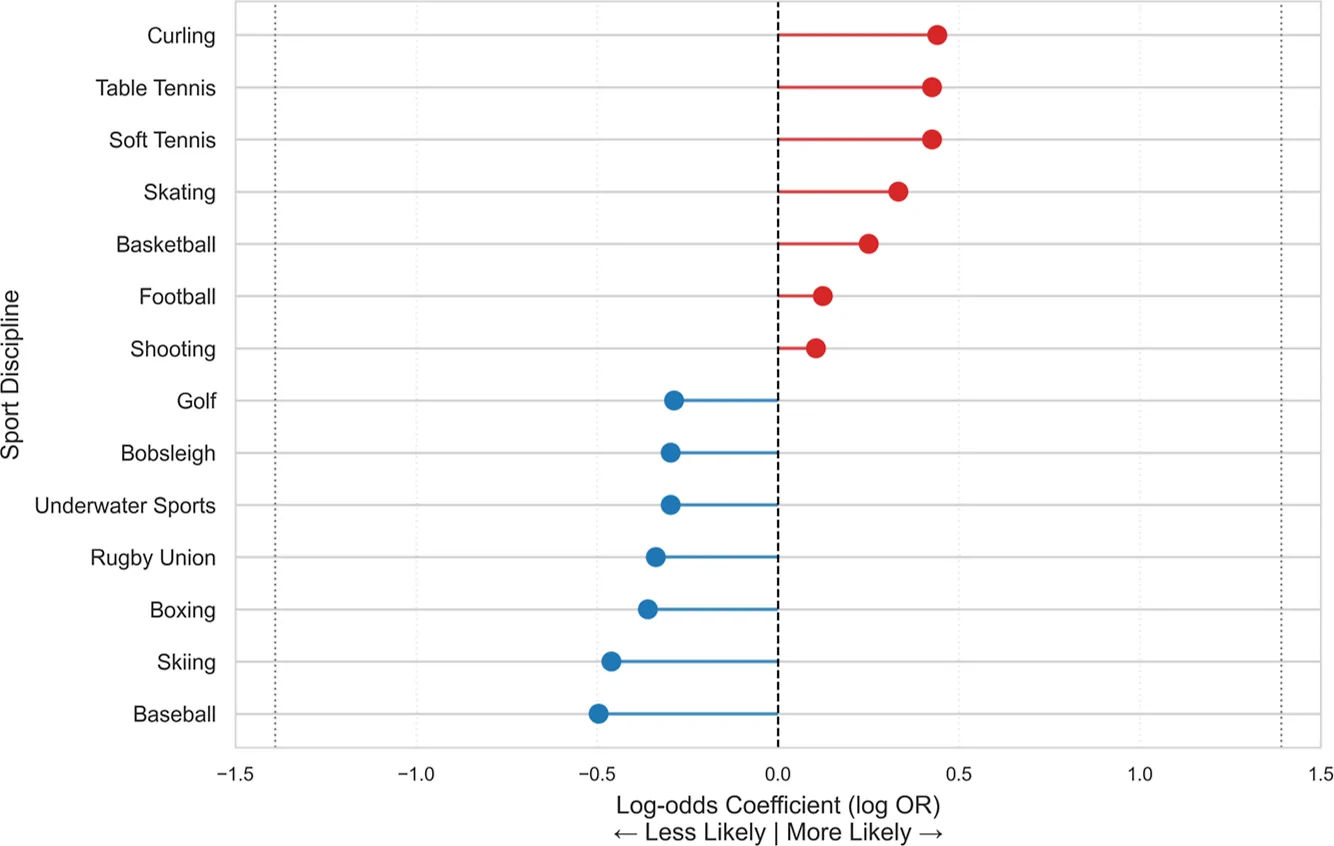
Exploratory L2-regularized logistic regression coefficients for the 15 most represented sport disciplines in the external test set. The analysis modeled ground-truth doping status using athlete sex and one-hot encoded sport categories. One sport discipline served as the reference category; therefore, coefficients for the 14 nonreference sport categories are displayed. The coefficients represent directional associations relative to the reference category and were interpreted descriptively because of the sparse subgroup sizes. They varied in direction around zero and showed no consistent sport-related pattern.

### SHAP-based Feature Attribution across Analytes

The lack of a monotonic relationship between feature coverage and SHAP attribution observed in Figure S9 indicates that infrequently observed analyte-level response features can contribute to sample-level classification when their peak-area response patterns provide discriminative information. These SHAP-based findings should be interpreted as model attribution results, not as evidence of concentration-dependent analytical sensitivity, limit-of-detection performance, or quantitative confirmation capability. Rather, they support the screening-triage relevance of informative analyte-level response features even when such features are infrequently observed in the data set.

### External Hold-Out Check for Confirmed Substances Absent from the Training-Positive Subset

As an additional external hold-out check, we examined positive samples that contained confirmed substances not represented in the training-positive subset (Table S12, Supporting Information). Four confirmed substances were present only in the external-positive subset: 19-NE, mibolerone, zeranol, and zeranol-M. Five positive samples in the external hold-out subset contained at least one of these substances, and all five were flagged by the fixed Rank 1 model at the predefined operating threshold, with predicted positive-class probabilities ranging from 0.810 to 0.990. Two cases were particularly informative: one contained zeranol-M and zeranol, both absent from the training-positive subset, and another contained mibolerone as the only confirmed substance. These findings indicate that the model was not limited to prioritizing samples containing substances represented among the training-positive cases. Rather, they support the value of the sample-level triage strategy for identifying suspicious integrated GC–MS/MS response profiles, including profiles involving confirmed substances not represented among the positive cases used for model training. This external hold-out check was evaluated at the sample level and should not be interpreted as establishing analyte-specific analytical sensitivity or quantitative confirmation performance for these substances.

### Potential Role of Nonlinear Feature Interactions

The stronger robustness of the Rank 1 stacking ensemble is compatible with the combination of complementary decision structures capable of representing both linear and nonlinear relationships among analyte-level peak-area response features. Linear and tree-based components may capture complementary aspects of the multianalyte response structure, including nonlinear relationships and feature interactions. Although the present study was not designed to isolate the causal contribution of each architectural component independently, this interpretation is consistent with the robustness observed for the top-ranked model across repeated-seed and bootstrap analyses.^32–34^

### Operational Integration for Routine Doping Control

The proposed framework offers substantial operational advantages for high-throughput doping control laboratories. In conventional workflows, rigorous dual-analyst review of GC–MS/MS chromatographic data requires approximately 16 labor hours per 100 samples. While manageable under routine conditions, this manual review process places significant pressure on laboratory turnaround times during large-scale testing campaigns, such as major international competitions, where rapid result reporting is critical. In contrast, the optimized AI model processes MS data in a fraction of a second per sample. Including I/O overhead, the framework can screen 10,000 samples in less than 3 h. This level of operational scalability enables an automated triage paradigm in which samples classified as negative are deprioritized, allowing highly trained human experts to focus on complex, atypical, or flagged cases and thereby improving both screening efficiency and analytical consistency. In addition, because the framework is retrainable, it may facilitate future extension as analytical target panels evolve and new labeled data become available.

The post hoc analysis of model-flagged current-end point negative samples further supports this operational role. As summarized in Table S10 of the Supporting Information, these samples were retained as false positives relative to the current GC–MS/MS end point-specific reference labels for all performance calculations and were not reclassified based on model scores. From an implementation perspective, this analysis illustrates how model-based prioritization can direct focused expert review toward end point-negative cases that may warrant broader contextual assessment.

### Methodological Considerations and Outlook

Several methodological considerations define the scope and next stage validation context for the present framework. These include the limited number of confirmed-positive samples, incomplete positive-case coverage across the 148 monitored analytes, dependence on MassHunter-derived targeted peak area response features, the absence of explicit statistical batch effect modeling, and data availability restrictions under regulatory and confidentiality requirements.

First, the proposed model depends on upstream targeted peak detection and peak integration. The final model was trained on MassHunter-exported analyte-level peak-area response values rather than raw GC–MS/MS chromatographic traces. Therefore, chromatographic and analytical-quality factors such as retention-time drift, potential target-peak misassignment, peak-shape distortion, coelution, background interference, integration-quality variation, and ion-ratio inconsistency were not directly modeled as independent predictor variables. These factors may influence the exported peak-area response values through the upstream targeted processing workflow, but the downstream model operates only on the resulting sample-by-analyte response matrix. Because all samples were processed using the same predefined MassHunter-based targeted workflow, the reported performance estimates should be interpreted within this standardized preprocessing environment. Robustness to alternative software platforms, software versions, integration settings, retention-time windows, response-export rules, or routine review criteria was not directly evaluated. Accordingly, implementation under different preprocessing environments would require harmonized target definitions and preprocessing criteria, followed by verification or revalidation of model performance. This consideration does not change the intended role of the framework as a screening-triage and review-prioritization tool based on routinely generated targeted GC–MS/MS response data.

Second, the data set was generated across multiple analytical batches rather than in a single analytical batch or an experimentally balanced batch design. Because samples were analyzed according to casework receipt and routine laboratory workflow order, the distribution of positive and negative samples across analytical batches reflected authentic antidoping casework conditions rather than an experimentally assigned batch structure. Negative samples predominated across batches, whereas confirmed positive samples were infrequent and unevenly distributed; accordingly, many analytical batches contained no confirmed positive samples. Potential batch-related analytical variability, including sample preparation, injection sequence, analytical date, reagent lot, column conditions, calibration status, instrument maintenance, and operator-dependent factors, was controlled and monitored at the analytical workflow level through routine system-suitability and QC procedures before data export. Batch-related operational metadata were not used as machine-learning input features, and no formal statistical batch effect assessment, batch effect correction, or batch covariate model was applied. Therefore, the reported performance should be interpreted within the standardized analytical workflow used in this study, and future batch-stratified validation using larger casework data sets will be important to further assess batch-related robustness.

Third, the number and chemical diversity of confirmed-positive samples were necessarily limited, even after aggregation of authentic casework over approximately four years. This constraint reflects the operational reality of antidoping laboratories rather than a readily remediable sampling limitation and should therefore be recognized as an inherent boundary of retrospective casework-based model development. The model was designed to prioritize suspicious urine samples from integrated GC–MS/MS response profiles under authentic screening conditions, rather than to serve as a separate analyte-specific detector for each monitored analyte. This sample-level design is aligned with the rare-positive antidoping screening setting, where positive cases for individual targets are necessarily sparse. The reported performance should be interpreted as sample-level screening-triage performance within the available authentic casework distribution, rather than as evidence of analyte-specific sensitivity, specificity, or quantitative confirmation capability for each of the 148 monitored GC–MS/MS target analytes. Incomplete positive-case coverage across the 148 monitored analytes therefore defines the boundary of analyte-specific inference, while the primary end point evaluated here remains sample-level review prioritization. Although the external hold-out analysis demonstrated successful sample-level flagging of positive cases involving substances absent from the training-positive subset, residual uncertainty remains for positive profiles not represented in the available data. False-negative risk therefore cannot be completely excluded in such previously unrepresented cases, and manual review should remain available as a safety backup during routine implementation. Spiked-urine or reference-material experiments can support analyte-specific response characterization, but they do not recapitulate authentic postexposure urinary profiles, including metabolism, excretion, parent–metabolite relationships, and casework-level interpretive context. As a complementary extension for settings in which confirmed-positive samples remain extremely sparse, future work may also explore anomaly detection approaches, including strategies that model authentic negative casework as a reference distribution, as supplementary tools for screening-prioritization tasks.^15,35^

Fourth, the current framework was developed using data generated on a single GC–MS/MS platform in one laboratory. Broader validation across WADA-accredited and related analytical laboratories, instrument configurations, and routine operating environments will therefore be required to establish generalizability and practical portability. Future studies should also examine how the framework can be extended across complementary analytical platforms, including LC–MS/MS-based screening settings, particularly as analytical panels evolve and newly prohibited substances or broader analyte classes are incorporated into routine screening menus.

Fifth, preanalytical timing variables were not explicitly incorporated as input features in the present study. While samples were handled within standardized WADA-aligned chain-of-custody and routine analytical workflows, nonroutine logistical exceptions may still introduce preanalytical variability. Evaluating the robustness of the present framework under such atypical transport or storage conditions remains an important objective for future validation studies. WADA standards explicitly require standardized sample transport, chain of custody, and documentation of irregularities, which supports treating this as a future robustness-validation issue rather than a core variable in the present routine screening data set.

Data-availability constraints are described in the Data Availability section; briefly, nonidentifiable aggregate feature-level summaries and reproducibility materials are provided to the extent permitted, whereas raw chromatograms and case-linked processed data remain restricted by regulatory and confidentiality requirements.

Taken together, these considerations define the appropriate interpretation scope of the present framework and a realistic validation pathway toward broader implementation of AI-assisted, high-throughput screening support in regulatory antidoping laboratories.

## Conclusion

This study demonstrates that a chemometric machine-learning framework can strengthen antidoping screening by supporting rapid and reliable screening-stage triage under analytically complex decision conditions. In routine antidoping testing, final interpretation remains an expert-driven process governed by multiple WADA-aligned technical and regulatory considerations, including analyte-specific criteria, relevant metabolites or markers, threshold-related rules for selected substances, and, where applicable, assessment of endogenous versus exogenous origin. This complexity creates a need for early stage triage strategies that can prioritize suspicious samples consistently while reducing unnecessary review burden on expert analysts.

The principal contribution of this study is the establishment of a robustness-validated and chemically interpretable machine learning framework for screening-stage triage using authentic human urine casework. Developed under the severe class imbalance and rare-positive conditions characteristic of routine antidoping practice, the framework was derived from systematic benchmarking of a large chemometric design space and evaluated through fixed external-validation hold-out testing and repeated seed- and bootstrap-based robustness analyses. The selected stacking-based ensemble achieved a stable high-recall screening performance and captured complementary multianalyte response patterns while maintaining chemical interpretability. SHAP-based analysis further identified the analyte-level response features contributing most strongly to the model output and showed chemically meaningful attribution patterns.

These findings support the practical use of machine learning as an additional triage layer within authentic antidoping workflows. Rather than replacing expert-driven regulatory interpretation, the framework can prioritize samples warranting closer review, thereby supporting more efficient and consistent screening stage decision making. It therefore provides a practical foundation for broader validation and implementation of high-throughput, AI-assisted triage support in regulatory antidoping laboratories.

## Supplementary Material













## Data Availability

The full processed 684 × 148 feature matrix, together with associated binary class labels and train/external-validation split assignments, is not publicly available because the data set was derived from authentic antidoping casework involving athlete urine samples. Even after removal of direct sample identifiers, unrestricted release of the complete analyte-level GC–MS/MS response matrix linked to sample-level labels and modeling partitions may disclose sensitive casework-derived response patterns and operational screening information. Therefore, public release of the full sample-level matrix is not permitted under the applicable data-governance and confidentiality constraints. To support reproducibility, the manuscript and Supporting Information provide detailed descriptions of feature construction, preprocessing settings, zero/nonzero handling rules, model-development procedures, threshold optimization, and validation strategy. To improve transparency without releasing sample-level casework-derived data, feature-level aggregate summary statistics for the processed 684 × 148 GC–MS/MS predictor matrix are provided in Table S13 of the Supporting Information. A focused source-code package comprising three runnable Python scripts and a README/user manual was prepared to support reproducibility of the main computational workflow. The package covers input integrity checking and locked stratified split definition, Stage 2 model screening and top 10 candidate selection, and Stage 3 reliability evaluation of 60 candidate pipelines across the screening, balanced, and forced-recall evaluation sets using repeated random seeds and bootstrap resampling. The package has been uploaded as reviewer-only Supporting Information for editorial and reviewer inspection. Unrestricted public release is not provided because the study involves antidoping casework-derived analytical information and regulatory laboratory contexts. Broader release would require institutional approval and applicable antidoping governance review.

## Notes

Robust chemometric machine learning framework for screening-stage triage in anti-doping urine analysis, GC–MS/MS screening of prohibited doping substances, RT and MRM transitions, analytical parameters and conditions for doping analysis via GC–MS/MS, MassHunter screening-stage batch-processing and peak-integration settings used to generate the machine-learning predictor matrix, summary of data set composition, class balance, and analyte representation, grid-search space and hyperparameter configurations for all evaluated chemometric machine-learning pipelines, performance metrics and configurations of the top 10 chemometric machine-learning pipelines selected to assess the classification precision–recall trade-off, external-validation performance and configurations of candidate pipelines across the screening and balanced frameworks and the auxiliary forced-recall evaluation, external hold-out check for confirmed substances absent from the training-positive subset, schematic workflow for urine sample preparation and GC–MS/MS analysis of prohibited substances, general antidoping analytical review workflow, representative raw chromatograms illustrating diverse interpretive scenarios encountered during routine manual review in antidoping analysis, normalized intensity distributions for all analyte-specific input features, confusion matrices of the top five ranked models evaluated on the external validation set, mean-only performance comparison of the five selected pipelines, receiver operating characteristic curve of the fixed Rank 1 model on the fixed external-validation hold-out set, post hoc review-priority ranking of all 684 samples using the fixed Rank 1 model, and relationship between feature importance and detection coverage of key doping-related analytes (PDF)
Full pipeline-level model-screening results for 10,920 model-output rows across 840 preprocessing/threshold-selection configurations (XLSX)
Post hoc all-684 review-priority ranking using the fixed Rank 1 model (XLSX)
All 24 model-flagged nonpositive samples from the post hoc all-684 review-priority analysis (XLSX)
Representative annotated operational boundary cases and corresponding 148-analyte response profiles (XLSX)
Feature-level summary statistics of the processed 684 × 148 GC–MS/MS predictor matrix (XLSX)

## Abbreviations

AI: artificial intelligence
CI: confidence intervals
ENN: edited nearest neighbors
ICAIA: International Conference on Artificial Intelligence and Applications
MRL: maximum residue limit
MRM: multiple reaction monitoring
MS: mass spectrometry
NCU: negative control urine
OOF: out-of-fold
PCU: positive control urine
RT: retention times
RUS: random under-sampling
SMOTE: synthetic minority oversampling technique
SVM: support vector machine
WADA: World Anti-Doping Agency
