# Supplementary material for: Robust Chemometric Machine Learning Framework for Screening-Stage Triage in Antidoping Urine Analysis
Source: Anal Chem. 2026 Sep 24;98(39):28728–44. doi: 10.1021/acs.analchem.6c02186 (PMC13647974; doi:10.1021/acs.analchem.6c02186)
Supplement: Supplementary file 1 [file ac6c02186_si_001.pdf]

Supporting Information

A Robust Chemometric Machine Learning Framework for Screening-Stage Triage in Anti-Doping Urine Analysis

Hana Park[a], Junghyun Son[a,*]

a. Doping Control Center, Korea Institute of Science and Technology, Seoul 02792, Republic of Korea

*Corresponding author: son.junghyun@kist.re.kr

Table of Contents

Contents	Page
Table S1. GC–MS/MS screening of prohibited doping substances: RT and MRM transitions	3
Table S2. Analytical parameters and conditions for doping analysis via GC–MS/MS	9
Table S3. MassHunter screening-stage batch-processing and peak-integration settings used to generate the machine-learning predictor matrix	10
Table S4. Summary of dataset composition, class balance, and analyte representation	13
Table S5. Grid-search space and hyperparameter configurations for all evaluated chemometric machine-learning pipelines	14
Table S6. Full pipeline-level model-screening results for 10,920 model-output rows across 840 preprocessing/threshold-selection configurations	Separate XLSX file
Table S7. Performance metrics and configurations of the top 10 chemometric machine-learning pipelines selected to assess the classification precision–recall tradeoff	18
Table S8. External-validation performance and configurations of candidate pipelines across the screening and balanced frameworks and the auxiliary forced-recall evaluation	20
Table S9. Post hoc all-684 review-priority ranking using the fixed Rank 1 model	Separate XLSX file
Table S10. All 24 model-flagged non-positive samples from the	Separate XLSX file

post hoc all-684 review-priority analysis	
Table S11. Representative annotated operational boundary cases and corresponding 148-analyte response profiles	Separate XLSX file
Table S12. External Hold-Out Check for Confirmed Substances Absent from the Training-Positive Subset	28
Table S13. Feature-level summary statistics of the processed 684 × 148 GC–MS/MS predictor matrix	Separate XLSX file
Figure S1. Schematic workflow for urine sample preparation and GC–MS/MS analysis of prohibited substances	29
Figure S2. General anti-doping analytical review workflow	30
Figure S3. Representative raw chromatograms illustrating diverse interpretive scenarios encountered during routine manual review in anti-doping analysis	31
Figure S4. Normalized intensity distributions for all analyte-specific input features	32
Figure S5. Confusion matrices of the top five ranked models evaluated on the external validation set	41
Figure S6. Mean-only performance comparison of the five selected pipelines	42
Figure S7. Receiver operating characteristic curve of the fixed Rank 1 model on the fixed external-validation hold-out set	43
Figure S8. Post hoc review-priority ranking of all 684 samples using the fixed Rank 1 model	44
Figure S9. Relationship between feature importance and detection coverage of key doping-related analytes	45

Table S1. GC–MS/MS screening of prohibited doping substances: RT and MRM transitions.

Compounds	RT (min)	MRM pair 1	CE 1	MRM pair 2	CE 2
**Exo-Steroids**					
1-Androstenediol	9.07	434.0 -> 405.0<br>434.0 -> 195.0	10<br>20	434.0 -> 377.0	10
1-Androstenedione	8.61	415.0 -> 221.0	20	194.0 -> 105.0	20
3β-hydroxy-5α-androst-1-en-17-one (1-Androsterone; 1-Epiandrosterone)	8.65	275.0 -> 155.0<br>432.0 -> 275.0	10<br>30	432.0 -> 290.0	10
1-Testosterone	8.92	432.0 -> 206.0	10	432.0 -> 194.0	10
5α-Androst-1-ene-3α-ol-17-one (1-Testosterone-M)	7.76	432.0 -> 290.0<br>432.0 -> 403.0	10<br>10	432.0 -> 275.0	20
4-OH-testosterone	12.64	520.0 -> 431.0	20	520.0 -> 225.0	25
Bolandiol	8.16	420.0 -> 240.0	5	420.0 -> 391.0	10
Bolasterone	11.96	315.0 -> 169.0	20	355.0 -> 169.0	30
7α,17α-Dimethyl-5β-androstane-3α,17β-diol (Bolasterone-M)	10.60	374.0 -> 269.0	10	374.0 -> 227.0	10
Calusterone	12.06	445.0 -> 355.0	10	445.0 -> 225.0	15
7β,17α-Dimethyl-5β-androstanediol (Calusterone-M)	10.11	269.0 -> 159.0	20	269.0 -> 213.0	10
4-Chloro-3α-hydroxy-androst-4-en-17-one (Clostebol-M1)	10.62	466.0 -> 431.0	10	466.0 -> 169.0	30
4-Chloro-5α-androst-3α-ol-17-one (Clostebol-M2)	11.30	468.0 -> 453.0	10	453.0 -> 363.0	15
4-Chloro-5β-androst-3α-ol-17-one (Clostebol-M3)	11.68	468.0 -> 453.0	10	453.0 -> 237.0	20
Ethisterone (Danazol-M1)	12.06	456.0 -> 441.0	10	456.0 -> 316.0	15
2α-OH-methylethisterone (2a-OH-methylethisterone; Danazol-M2)	13.11	558.0 -> 193.0	40	558.0 -> 403.0	20
Dehydrochloromethyltestosterone (DHCMT; Turinabol)	12.96	478.0 -> 240.0	10	478.0 -> 225.0	30
6β-Hydroxy-dehydrochloromethyltestosterone (6b-OH-DHCMT; 6β-OH-turinabol)	13.21	315.0 -> 227.0<br>317.0 -> 163.0	15<br>20	227.0 -> 163.0	10
4-chloro-18-nor-17β-hydroxymethyl,17α-methyl-5β-androst-13-en-3α-ol (DHCMT-M3)	11.70	379.0 -> 343.0	5	379.0 -> 253.0	15
4-chloro-18-nor-17α-hydroxymethyl,17β-methyl-5β-androst-13-en-3α-ol (DHCMT-epiM3)	10.35	379.0 -> 343.0	5	379.0 -> 253.0	15
4-chloro-18-nor-17β-hydroxymethyl,17α-methyl androsta-4,13-dien-3α-ol (DHCMT-M4)	11.47	377.0 -> 149.0	15	287.0 -> 125.0	30
4-chloro-18-nor-17α-hydroxymethyl,17β-methyl androsta-4,13-dien-3α-ol (DHCMT-epiM4)	10.15	377.0 -> 149.0	15	287.0 -> 125.0	30
Desoxymethyltestosterone (DMT)	5.36	345.0 -> 255.0	10	345.0 -> 201.0	20

17α-methyl-5α-androstan-2β,3α,17β-triol (DMT-II)	12.10	523.0 -> 253.0	20	523.0 -> 343.0	15
17α-methyl-5α-androstan-2α,3α,17β-triol (DMT-M1)	9.18	523.0 -> 253.0	20	523.0 -> 343.0	15
18-nor-17,17-dimethyl-5α-androst-13-en-2ξ,3α-diol (DMT-M2)	5.77	433.0 -> 253.0	15	433.0 -> 147.0	25
2α-Methyl-5α-androstan-3α-ol-17-one (Drostanolone-M)	8.15	448.0 -> 343.0	15	448.0 -> 169.0	30
Ethylestrenol	6.18	270.0 -> 241.0	10	270.0 -> 185.0	20
Fluoxymesterone	12.98	552.0 -> 462.0	10	552.0 -> 407.0	15
9α-F-17α-methyl-4-androsten-3α,6β,11β,17β-tetrol (Fluoxymesterone-M1)	12.62	642.0 -> 447.0<br>642.0 -> 427.0	10<br>20	642.0 -> 357.0	20
9α-Fluoro-17,17-dimethyl-18-norandrostan-4,13-diene-11β-ol-3-one (Fluoxymesterone-M2)	8.92	462.0 -> 208.0	20	462.0 -> 357.0	15
6β-OH-fluoxymesterone (Fluoxymesterone-M3)	13.36	640.0 -> 605.0<br>640.0 -> 515.0	25<br>20	640.0 -> 425.0	25
4-androstene-17α-methyl-11α,17β-diol-3-one (Formebolone-M2)	12.55	534.0 -> 389.0	20	534.0 -> 301.0	20
16β-Hydroxyfurazabol (16b-OH-furazabol)	13.63	490.0 -> 231.0	15	490.0 -> 143.0	25
Mestanolone	11.21	216.0 -> 159.0	10	216.0 -> 187.0	10
1α-Methyl-5α-androstane-3α-ol-17-one (Mesterolone-M1)	9.03	433.0 -> 343.0	5	433.0 -> 253.0	10
1α-Methyl-5α-androstane-3α,17β-diol (Mesterolone-M2)	9.38	450.0 -> 255.0	20	345.0 -> 255.0	10
Epimetendiol (Methandienone-M1)	6.39	358.0 -> 301.0	15	358.0 -> 169.0	30
6β-Hydroxymethandienone (6b-OH-methandienone; Methandienone-M2)	12.78	517.0 -> 337.0	20	517.0 -> 429.0	20
17,17-Dimethyl-18-norandrosta-1,4,13(14)-trien-3-one (Methandienone-M3)	5.13	354.0 -> 148.0	10	354.0 -> 133.0	20
17β-hydroxymethyl-17α-methyl-18-norandrost-1,4,13-triene-3-one (Methandienone-M9)	9.67	442.0 -> 236.0	10	442.0 -> 339.0	10
Metenolone	10.63	446.0 -> 195.0	20	446.0-> 208.0	15
Methylene-5α-androstan-3α-ol-17-one (Metenolone-M)	8.63	446.0 -> 431.0	10	446.0 -> 251.0	20
Methasterone	12.00	462.0 -> 419.0	10	332.0 -> 156.0	10
2α,17α-dimethyl-5α-androstane-3α,17β-diol (Methasterone-M)	9.70	449.0 -> 269.0	10	449.0 -> 359.0	10
Methylclostebol	13.15	480.0 -> 390.0	10	480.0 -> 335.0	20
4-chloro-17α-methyl-androst-4-ene-3α,17β-diol (Methylclostebol-M)	12.16	357.0 -> 169.0	20	357.0 -> 227.0	20
Methyldienolone	11.74	430.0 -> 285.0<br>430.0 -> 340.0	20<br>15	430.0 -> 246.0	20
Methyl-1-testosterone	10.63	206.0 -> 165.0	20	446.0 -> 206.0	10
17α-methyl-5α-androst-1-ene-3α,17β-diol (Methyl-1-testosterone-M; M-1-T-M)	9.37	448.0 ->419.3<br>448.0 ->201.2	15<br>35	448.0 -> 216.0	10

Methylnortesoterone	10.99	287.0 -> 272.0	20	287.0 -> 197.0	10
Methylstenebolone	11.64	370.0 -> 220.0<br>370.0 -> 231.0	10<br>30	370.0 -> 245.0	20
2,17α-dimethyl-16z,17β-dihydroxy-5α-androst-1-en-3-one (Methylstenebolone-M; MSB-M)	13.16	550.0 -> 117.0<br>208.0 -> 193.0	15<br>10	548.0 -> 208.0	15
Methyltestosterone (MT)	11.80	446.0->301.0	20		
17α-Methyl-5α-androstane-3α,17β-diol (Methyltestosterone-M1)	9.08	435.0 -> 255.0	15	435.0 -> 173.0	30
17α-Methyl-5β-androstane-3α,17β-diol (Methyltestosterone-M2)	9.17	270.0 -> 213.0	10	270.0 -> 199.0	20
Mibolerone	11.23	431.0 -> 341.0	10	301.0 -> 73.0	40
Norboletone	12.98	460.0 -> 301.0	10	301.0 -> 143.0	30
13β,17α-Diethyl-5α-gonane-3α,17β-diol (Norboletone-M alpha)	11.82	435.0 -> 255.0	20	435.0 -> 345.0	10
13β,17α-Diethyl-5β-gonane-3α,17β-diol (Norboletone-M beta)	12.16	435.0 -> 255.0<br>144.0 -> 75.0	20<br>20	435.0 -> 345.0	10
Norclostebol	12.60	452.0 -> 321.0	20	452.0 -> 417.0	5
4-chloro-3α-hydroxyestr-4-en-17-one (Norclostebol-M1)	9.83	452.0 -> 437.0	10	437.0 -> 401.0	10
4-chloro-3α-hydroxy-5β-estran-17-one (Norclostebol-M2)	10.52	439.0 -> 349.0	10	439.0 -> 223.0	15
4-chloro-3α-hydroxy-5α-estran-17-one (Norclostebol-M3)	10.06	439.0 -> 349.0	10	439.0 -> 313.0	10
Norethandrolone	12.53	446.0 -> 287.0	20	446.0 -> 300.0	20
17α-Ethyl-5β-estrane-3α-17β-diol (Norethandrolone-M1)	10.64	421.0 -> 241.0	15	331.0 -> 241.0	10
17α-Hydroxyethyl-5β-estrane-3α,17β-diol (Norethandrolone-M2)	12.63	421.0 -> 331.0	5	421.0 -> 241.0	15
Oxabolone	12.27	506.0 -> 147.0	35	506.0 -> 195.0	30
4-Hydroxyestr-4-en-3,17-dione (Oxabolone-M)	11.05	504.0 -> 219.0	35	504.0 -> 234.0	20
3α,4α-dihydroxy-5α-estran-17-one (Oxabolone-M3)	8.71	493.0 -> 223.0	20	493.0 -> 313.0	15
Oxandrolone	12.07	363.0 -> 161.0<br>308.0 -> 161.0	20<br>20	321.0 -> 95.0	35
Epioxandrolone (Oxandrolone-M)	10.16	308.0 -> 176.0	5	321.0 -> 189.0	20
17α-hydroxymethyl-17β-methyl-18-nor-2-oxa-5α-androsta-13-en-3-one (Oxandrolone LTM)	9.84	273.0 -> 213.0	10	273.0 -> 161.0	20
17α-hydroxymethyl-17β-methyl-18-nor-2-oxa-5α-androsta-13-en-3-one (Oxandrolone-LTM1)	10.15	273.0 -> 213.0	10	273.0 -> 161.0	20
Oxymesterone	13.02	534.0 -> 301.0<br>389.0 -> 169.0	40<br>30	389.0 ->257.0	20
2z-hydroxymethyl-17α-methyl-5α-androstan-3α,6β,17β-triol (Oxymetholone-M)	13.27	495.0 -> 315.0	10	495.0 -> 225.0	15

4,17α-dihydroxy-17β-methylandrost-4-en-3-one (17-epioxymesterone)	12.57	534.0->301.0<br>389.0->257.0	40<br>20	389.0->169.0	30
3'-OH-prostanozolol (3'-OH-prostanozol)	13.45	544.0 -> 254.0	30	529.0 -> 347.0	45
4α/β-OH-prostanozol (4a/b-OH-prostanozol)	13.63	544.0 -> 112.0	40	544.0 -> 211.0	30
3'-OH-stanozolol	13.75	560.0 -> 520.0<br>560.0 -> 254.0	10<br>15	545.0 -> 387.0	50
4β-OH-stanozolol (4b-OH-stanozolol)	13.76	545.0 -> 381.0	40	380.0 -> 213.0	15
Stenebolone	9.79	446.0 -> 208.0	15	446.0 -> 220.0	15
2β-methyl-3α-hydroxy-5α-androstan-17-one (Stenebolone-M1)	8.44	448.0 -> 433.0	10	448.0 -> 343.0	15
2-methyl-3α-hydroxy-5α-androst-1-en-17-one (Stenebolone-M2)	8.68	446.0 -> 275.0	15	446.0 -> 290.0	10
Epitrenbolone	9.38	307.0 -> 291.0	15	307.0 -> 275.0	20
3α-OH-tibolone (3a-OH-tibolone)	9.85	443.0 -> 193.0	30	443.0 -> 167.0	25
3β-OH-tibolone (3b-OH-tibolone)	8.92	443.0 -> 193.0	30	443.0 -> 167.0	25
Δ4-tibolone (delta4-tibolone)	11.49	441.0 -> 247.0	15	441.0 -> 351.0	10
**Endo-Steroids**					
19-Norandrostenedione	8.85	416.0 -> 401.0	10	416.0 -> 311.0	20
Androstenediol	9.08	434.0 -> 329.0	10	434.0 -> 239.0	20
Androstenedione	9.65	430.0 -> 415.0	20	430.0 -> 209.0	20
Boldenone	9.73	430.0 -> 206.0<br>206.0 -> 165.0	15<br>15	325.0 -> 229.0	20
5β-Androst-1-en-17β-ol-3-one (Boldenone-M)	6.38	194.0 -> 163.0<br>432.0 -> 179.0	20<br>40	194.0 -> 149.0<br>432.0 -> 194.0	20<br>10
Boldione	9.38	428.0 -> 206.0	15	413.0 -> 229.0	20
Androsta-1,4-dien-6β-ol-3,17-dione (Boldione-M)	7.97	518.0->503.0	15	518.0->428.0	20
Dihydrotestosterone (DHT)	9.44	434.0 ->405.0	10	434.0 -> 377.0	10
5α-Androstan-3,17-dione (5a-dione)	9.04	432.0->275.0	20	432.0->290.0	15
5β-Androstan-3,17-dione (5b-dione)	6.21	432.0->275.0	20	432.0->290.0	15
Androst-5-en-3β-ol-17-one (Dehydroepiandrosterone; DHEA)	8.77	432.0 -> 417.0	5	432.0 -> 327.0	15
Testosterone	9.98	432.0 -> 209.0	20		
Epiandrosterone	8.87	434.0 -> 419.0	10	419.0 -> 239.0	10
5α-Androst-2-ene-17-one	3.78	344.0 -> 329.0	15	329.0 -> 135.0	15

5α-Androstan-3α,17α-diol (5a-Androstan-3a 17a-diol)	6.33	256.0 -> 199.0	20	256.0 -> 185.0	10
5α-Androstan-3α,17β-diol (5a-diol)	7.88	436.0 ->241.0	15	346.0 -> 241.0	10
5α-Androstan-3β,17β-diol (abb-diol)	9.21	436.0 -> 241.0	15	436.0 -> 346.0	5
5β-Androstan-3α,17β-diol (5b-diol)	7.98	346.0 -> 256.0	5	346.0 -> 241.0	10
7α-OH-DHEA (7a-OH-DHEA)	9.35	415.0 -> 325.0	10	325.0 -> 235.0	20
7β-OH-DHEA (7b-OH-DHEA)	11.71	415.0 -> 325.0	10	325.0 -> 235.0	20
4-Androstenediol	8.88	434.0 -> 195.0	20	434.0 -> 127.0	40
5-Androstendione	9.65	430.0 -> 415.0	20	430.0 -> 209.0	20
7-keto-DHEA	12.21	518.0 -> 323.0	30	518.0 -> 429.0	20
19-Norandrosterone (19-NA)	6.16	405.0 -> 315.0	5	405.0 -> 225.0	15
19-Noretiocholanolone (19-NE)	7.00	405.0 -> 315.0	5	405.0 -> 225.0	10
4-Androsten-3α,17α-diol (4-Androsten-3a 17a-diol)	6.01	254.0 -> 239.0	10	239.0 -> 183.0	20
4-Androsten-3β,17α-diol (4-Androsten-3b 17a-diol)	7.92	254.0 -> 183.0	20	254.0 -> 197.0	20
Androst-5-ene-3α,17β-diol	7.56	305.0 -> 215.0	10	305.0 -> 119.0	30
Androst-5-ene-3β,17α-diol	8.36	239.0 -> 183.0	20	254.0 -> 239.0	10
Androsterone	7.53	434.0 -> 239.0	15	434.0 -> 329.0	15
Epi-dihydrotestosterone (Epi-DHT)	8.59	434.0 -> 127.0	40	434.0 -> 195.0	30
Epitestosterone	9.17	432.0 -> 209.0	20		
Etiocholanolone	7.67	434.0 -> 239.0	15	434.0 -> 329.0	15
11β-OH-androsterone (11b-OH-an)	10.27	522.0 -> 283.0	15	522.0 -> 417.0	15
11β-OH-etiocholanolone (11b-OH-etio)	10.51	522.0 -> 283.0	15	522.0 -> 417.0	15
11-keto-androsterone (11-keto-An)	8.71	520.0 -> 415.0	15	520.0 -> 505.0	5
11-keto-etiocholanolone (11-keto-Etio)	8.80	520.0 -> 415.0	15	520.0 -> 505.0	5
Pregnanediol	12.08	117.0 -> 117.0	20	117.0 -> 115.0	20
**Other Substances**					
Clenbuterol	2.84	335.0 -> 300.0	10	337.0 -> 302.0	10
Zilpaterol	4.52	308.0 -> 218.0<br>405.0 -> 308.0	15<br>5	308.0 -> 203.0	30
α-Zearalanol (Zeranol)	12.52	433.0 -> 295.0	25	433.0 -> 389.0	10
β-Zearalanol (Zeranol-M)	12.60	433.0 -> 295.0	25	433.0 -> 389.0	10
α-Zearalenol (a-Lenol)	12.79	446.0->317.0	25	536.0->317.0	40

β-Zearalenol (b-Lenol)	12.88	446.0->317.0	25	536.0->317.0	40
4-OH-cyclofenil	12.78	422.0 -> 367.0	20	422.0 -> 343.0	25
6β-Hydroxybromantane (6b-OH-bromantan)	8.73	395.0 -> 133.0	15	393.0 -> 171.0	20
4-Androsten-3,6,17-trione (6-OXO)	12.19	516.0 -> 411.0	15	516.0 -> 205.0	20
4-Androsten-6α-ol-3,17-dione (6-OXO-M)	12.35	518.0 -> 319.0	30	518.0 -> 413.0	20
Arimistane (Arimistane-P)	7.48	428.0 -> 233.0<br>428.0 -> 323.0	20<br>15	428.0 -> 169.0	20
7-hydroxyandrost-3,5-diene-17-one (Arimistane-M)	7.16	325.0 -> 235.0<br>430.0 -> 325.0	10<br>15	235.0 -> 178.0	40
Carphedone	2.47	272.0 -> 104.0	20	272.0 -> 257.0	10
Formestane	12.54	518.0 ->221.0	30	518.0 -> 429.0	15
bis-4-cyanophenyl-methanol (Letrozole-M)	4.07	291.0 -> 160.0	10	291.0 -> 217.0	20
Morphine	7.14	429.0 -> 287.0	20	429.0 -> 414.0	15
Salbutamol	2.43	369.0 -> 191.0	20	369.0 -> 295.0	15
3-OH-4-methoxy-tamoxifen (Tamoxifen-M)	13.22	489.0 -> 72.0	10	489.0 -> 58.0	25
Tetrahydronorethisterone (Norethisterone-M)	8.54	431.0 -> 193.0	20	431.0 -> 167.0	20
THC	4.80	386.0 -> 371.0	10	386.0 -> 315.0	10
THC-COOH	11.89	473.0 -> 355.0	30	371.0 -> 315.0	15
mono-TMS-An	6.32	347.0 -> 271.0	10		

Table S2. Analytical parameters and conditions for doping analysis via GC–MS/MS.

**Gas chromatography: Agilent 7890B GC**<br>**Mass spectrometer: Agilent 7010 QQQ**	
**Column**	Agilent HP-Ultra 1<br>(17 m, length × 0.2 mm, I.D. × 0.11 μm film thickness)
**Injection parameters**	Injection volume: 1 μL<br>Injection mode: Split 10:1<br>Flow rate: 0.7 mL/min (He)<br>Inlet temperature: 280 ℃
**Oven program**	Initial temperature: 180 °C<br>Ramp 1: 20 °C/min to 200 °C<br>Ramp 2: 5 °C/min to 210 °C, hold 4 min<br>Ramp 3: 5 °C/min to 220 °C, hold 2.5 min<br>Ramp 4: 50 °C/min to 320 °C, hold 2.5 min
**Runtime**	16 min
**MS parameters**	Ionization mode: EI<br>Acquisition mode: MRM<br>Solvent delay: 2 min<br>Source temperature: 250 ℃<br>Quad temperature: 150 ℃<br>Interface temperature: 300 ℃<br>Quench gas: 2.25 mL/min (He)<br>Collision gas: 1.5 mL/min (N_2)

Table S3. MassHunter screening-stage batch-processing and peak-integration settings used to generate the machine-learning predictor matrix.

Category	Parameter	Setting used in this study
Software	Data-processing software	Agilent MassHunter Quantitative Analysis, version 10.1
Software	Build version	10.1.733.0
Processing workflow	Processing purpose	Screening-stage targeted batch processing for construction of the machine-learning predictor matrix
Processing workflow	Processing type	Targeted analyte-level response extraction; not untargeted feature detection
Target-peak processing	Target information	Fixed MRM transitions and analyte-specific retention-time information
Target-peak processing	Peak assignment	Software-defined target-peak assignment based on the predefined targeted batch-processing method
Target-peak processing	Peak integration	Automated integration
Integration	Integrator	Agile2
Integration	Quantitation basis	Peak area
Integration	Peak-height quantitation	Not used
Agile2 integration settings	Data point sampling	1
Agile2 integration settings	Smoothing	False
Agile2 integration settings	Detection filtering	FivePoint
Agile2 integration settings	Start threshold	0.2
Agile2 integration settings	Stop threshold	0
Agile2 integration settings	Peak location	Top

Category	Parameter	Setting used in this study
Agile2 integration settings	Baseline reset	5
Agile2 integration settings	Baseline valley	100
Agile2 integration settings	Baseline preference	Tangent
Baseline/background settings	Additional smoothing	Not applied
Baseline/background settings	Dynamic background subtraction	Disabled
Retention-time settings	Retention-time tolerance unit	Minutes
Retention-time settings	Retention-time window	10%
Retention-time settings	Left retention-time delta range	0.045–0.400 min
Retention-time settings	Right retention-time delta range	0.100–0.300 min
Retention-time settings	Most common retention-time tolerance	±0.2 min
Automated peak selection	Peak selection criterion	CloseRT
Automated peak selection	Peak filter threshold type	PeakAreaPctThreshold
Automated peak selection	Peak-area percentage threshold	5%
Noise settings	Noise algorithm	RMS
Noise settings	Standard-deviation multiplier	5

Category	Parameter	Setting used in this study
User intervention	Routine review	Limited to review of exported results, including chromatographic alignment checks and verification that automated peak integration was reasonable
User intervention	Manual peak-by-peak reintegration	Not performed
Final exported matrix	Matrix structure	Sample-by-analyte response matrix
Final exported matrix	Final predictor matrix size	684 samples × 148 analyte-level response features
Final exported matrix	Model input features	Integrated peak-area response values for primary MRM transitions
Final exported matrix	Variables not exported as model predictors	Raw chromatograms, retention-time deviation, ion-ratio deviation, peak-shape descriptors, signal-to-noise ratios, and integration-quality information

Table S4. Summary of dataset composition, class balance, and analyte representation. Only aggregated cohort-level summaries are provided to protect case confidentiality; no sample identifiers, case identifiers, or sample-level raw analytical values are disclosed.

(a) Cohort composition and class balance

Cohort	Total samples	Positive samples	Negative samples	Positive rate
Training	547	45	502	8.2%
External validation	137	11	126	8.0%
Total	684	56	628	8.2%

(b) Aggregate analyte representation among confirmed-positive samples

Metric	Count
Total monitored prohibited substances	148
Confirmed-positive samples (class-label count)	56
Distinct prohibited substances represented among confirmed-positive samples	45
Distinct prohibited substances represented in training positive samples	41
Distinct prohibited substances represented in external validation positive samples	13
Detected prohibited substances per annotated positive sample	1-8

Counts for the training and external validation cohorts are not additive because some prohibited substances were represented in both cohorts.

Table S5. Grid-Search Space and Hyperparameter Configurations for All Evaluated Chemometric Machine-learning pipelines.

This table summarizes the complete grid-search space explored for all chemometric machine-learning pipelines, including classifier configurations and hyperparameter ranges for base and ensemble models. Common grid-search constraints applied uniformly across all models are also reported for completeness.

(a) Base Model Hyperparameters

Model	Hyperparameter(s)	Value / Description
**Logistic Regression (LR_L1)**	penalty	L1 (saga solver, max_iter = 5000)
	C	1.0 (inverse regularization strength)
	class_weight	none / balanced (grid parameter)
	random_state	42–71 (applied to stochastic solver)
**Linear SVM (LinearSVM_calibrated)**	C	1.0 (margin penalty)
	penalty	L2 (default)
	calibration	Platt sigmoid (CalibratedClassifierCV, cv=3)
	class_weight	none / balanced
	random_state	42–71 (applied to calibration split)
**Random Forest (RF)**	n_estimators	600 (criterion = gini, max_features = sqrt)
	class_weight	none / balanced
	random_state	42–71 (applied to bootstrapping)
**XGBoost (XGB)**	n_estimators	800
	max_depth	5
	learning_rate	0.1
	subsample	0.8 (row sampling per tree)
	colsample_bytree	1.0
	reg_lambda	1.0 (L2 regularization)
	scale_pos_weight	1 or (n_neg / n_pos) when

		balanced
	objective	binary:logistic (eval_metric = logloss)
	random_state	42–71 (applied to boosting process)
**Gaussian Naïve Bayes**	priors	None (inferred from data)
	var_smoothing	1e−9 (numerical stability)
	random_state	– (deterministic model)
**KNN**	n_neighbors	5
	metric	Euclidean
	weights	uniform
	random_state	– (deterministic model)

(b) Ensemble Model Configurations

**Ensemble model**	**Constituent base models**	**Decision rule / Meta-learning**	**Hyperparameter (s)**	**Threshold (τ) optimization**	**Class weight application**
**OR Ensemble**	6 base models: LR_L1, LinearSVM_calibrated, RF, XGB, GaussianNB, KNN	Positive if any base model predicts 1 (union of positives).	–	– (uses each base model's thresholded predictions)	Class weighting was applied only at the base-model training stage (grid: none or balanced); no additional weighting was introduced at the ensemble layer.
**AND Ensemble**	6 base models: LR_L1, LinearSVM_calibrated, RF, XGB, GaussianNB, KNN	Positive only if all base models predict 1 (intersection).	–	– (uses each base model's thresholded predictions)	Class weighting was applied only at the base-model training stage (grid: none or balanced); no additional weighting was introduced at the ensemble layer.
**MajorityVote (k=2)**	6 base models: LR_L1, LinearSVM_	Positive if ≥2 base models predict 1.	k = 2	– (uses each base model's thresholded predictions)	Class weighting was applied only at the base-model training stage

	calibrated, RF, XGB, GaussianNB, KNN				(grid: none or balanced); no additional weighting was introduced at the ensemble layer.
**MajorityVote (k=3)**	6 base models: LR_L1, LinearSVM_calibrated, RF, XGB, GaussianNB, KNN	Positive if ≥3 base models predict 1.	k = 3	– (uses each base model's thresholded predictions)	Class weighting was applied only at the base-model training stage (grid: none or balanced); no additional weighting was introduced at the ensemble layer.
**MajorityVote (k=4)**	6 base models: LR_L1, LinearSVM_calibrated, RF, XGB, GaussianNB, KNN	Positive if ≥4 base models predict 1.	k = 4	– (uses each base model's thresholded predictions)	Class weighting was applied only at the base-model training stage (grid: none or balanced); no additional weighting was introduced at the ensemble layer.
**SoftVote (probability averaging)**	6 base models: LR_L1, LinearSVM_calibrated, RF, XGB, GaussianNB, KNN	Mean of predicted probabilities from all base models; then thresholded.	–	F-β maximization (β = 0.5, 1.0, 2.0) OR Recall-floor (≥0.999 or ≥0.995) OR Classification-precision floor (≥0.80); τ selected on 5-fold OOF and applied to external test.	Class weighting was applied only at the base-model training stage (grid: none or balanced); no additional weighting was introduced at the ensemble layer.
**Stacking (meta-learner)**	6 base models: LR_L1, LinearSVM_calibrated, RF, XGB, GaussianNB, KNN	Logistic Regression meta-model trained on 5-fold OOF probabilities; applied to external test.	LR meta: max_iter=2000; class weight = none or balanced according to the pipeline class-weight setting; random state =	F-β maximization (β = 0.5, 1.0, 2.0) OR Recall-floor (≥0.999 or ≥0.995) OR Classification-precision floor	Base/meta: grid none/balanced where supported; XGBoost balanced mode: scale_pos_weight; KNN/GaussianN

	42–71	(≥0.80); τ selected on 5-fold OOF and applied to external test.	B: not applicable.

(c) Common grid-search constraints applied across all models

Category	Values / Description
**Feature Scaling**	None / RobustScaler / StandardScaler / MinMaxScaler / QuantileTransformer (uniform, normal) / PowerTransformer (Yeo–Johnson)
**Imputation**	Not applied. The final predictor matrix was required to contain complete numerical feature values. Blank or non-numeric feature entries were treated as input-integrity errors, while no exported analyte-level response values had already been coded as 0 during post-export matrix alignment.
**Resampling**	None / RandomUnderSampler (ratio = 0.1, 0.2, 0.333, 0.5, 0.7, 1.0) / SMOTE / SMOTE–Tomek / SMOTE–ENN
**Class weight**	None / Balanced
**Validation**	Stratified 5-fold CV for OOF + fixed external-validation hold-out set
**Threshold optimization**	Fβ-maximization (β = 0.5, 1, 2) / Recall-floor (≥0.995, ≥0.999) / Classification-precision floor (≥0.80)
**Seeds**	30 repetitions (42–71)
**Bootstrap**	5000 iterations for confidence intervals (CIs)
**Evaluation metrics**	Classification precision, recall, F1, accuracy, AUC, and AUPRC, evaluated across multiple random seeds with 95% bootstrap confidence intervals.

Table S7. Performance Metrics and Configurations of the Top 10 Chemometric Machine-Learning Pipelines Selected to Assess the Classification Precision–Recall Tradeoff.

This table summarizes the performance metrics and corresponding pipeline configurations of the ten highest-ranked models selected from each of the screening and balanced analytical frameworks to evaluate the classification precision–recall tradeoff. Models were ranked based on internal validation performance. Reported metrics include precision, recall, F1-score, accuracy, area under the receiver operating characteristic curve (AUC), and area under the precision–recall curve (AUPRC). Precision denotes classification precision, i.e., positive predictive value, not analytical-method precision.

(a) Top ten pipelines selected under the screening framework

Rank	Model	Sampler	ClassWeight	Strategy	Scaler	beta	Recall	Precision	F1	Accuracy	ROC_AUC	AUPRC
1	MajorityVote(k=3)	RUS_0.1	none	fbeta	QuantileNormal	0.5	1.000	0.733	0.846	0.971	0.985	0.736
2	MajorityVote(k=4)	RUS_0.333	balanced	fbeta	NONE	2	1.000	0.733	0.846	0.971	0.984	0.727
3	MajorityVote(k=2)	RUS_1.0	none	fbeta	NONE	0.5	1.000	0.733	0.846	0.971	0.982	0.718
4	MajorityVote(k=2)	RUS_0.5	balanced	fbeta	NONE	0.5	1.000	0.733	0.846	0.971	0.979	0.674
5	MajorityVote(k=2)	SMOTE	none	precision_floor	PowerYJ	0.5	1.000	0.688	0.815	0.964	0.993	0.886
6	MajorityVote(k=2)	SMOTE	balanced	precision_floor	PowerYJ	0.5	1.000	0.688	0.815	0.964	0.993	0.886
7	MajorityVote(k=2)	SMOTE_TOMEK	none	precision_floor	PowerYJ	0.5	1.000	0.688	0.815	0.964	0.993	0.886
8	MajorityVote(k=2)	SMOTE_TOMEK	balanced	precision_floor	PowerYJ	0.5	1.000	0.688	0.815	0.964	0.993	0.886
9	MajorityVote(k=2)	NONE	none	fbeta	QuantileNormal	0.5	1.000	0.688	0.815	0.964	0.986	0.793
10	MajorityVote(k=3)	RUS_0.7	balanced	fbeta	NONE	1	1.000	0.688	0.815	0.964	0.982	0.718

(b) Top ten pipelines selected under the balanced framework

Rank	Model	Sampler	ClassWeight	Strategy	Scaler	beta	Recall	Precision	F1	Accuracy	ROC_AUC	AUPRC
1	MajorityVote(k=3)	RUS_0.1	none	fbeta	QuantileNormal	0.5	1.000	0.733	0.846	0.971	0.985	0.736
2	MajorityVote(k=4)	RUS_0.333	balanced	fbeta	NONE	2	1.000	0.733	0.846	0.971	0.984	0.727
3	MajorityVote(k=2)	RUS_1.0	none	fbeta	NONE	0.5	1.000	0.733	0.846	0.971	0.982	0.718
4	MajorityVote(k=2)	RUS_0.5	balanced	fbeta	NONE	0.5	1.000	0.733	0.846	0.971	0.979	0.673
5	LinearSVM_calibrated	SMOTE_TOMEK	none	fbeta	Robust	1	0.727	1.000	0.842	0.978	0.820	0.787
6	LinearSVM_calibrated	SMOTE_TOMEK	none	fbeta	Robust	2	0.727	1.000	0.842	0.978	0.820	0.787
7	LinearSVM_calibrated	SMOTE_TOMEK	balanced	fbeta	Robust	1	0.727	1.000	0.842	0.978	0.820	0.787
8	LinearSVM_calibrated	SMOTE_TOMEK	balanced	fbeta	Robust	2	0.727	1.000	0.842	0.978	0.820	0.787
9	MajorityVote(k=2)	SMOTE	none	fbeta	Robust	0.5	0.909	0.769	0.833	0.971	0.987	0.828
10	SoftVote	SMOTE	none	fbeta	Robust	1	0.909	0.769	0.833	0.971	0.987	0.891

Table S8. External-Validation Performance and Configurations of Candidate Pipelines across the Screening and Balanced Frameworks and the Auxiliary Forced-Recall Evaluation.

This table summarizes the external-validation performance and corresponding pipeline configurations of the internally selected top 10 candidate configurations and their stacking-based extensions. For the screening and balanced frameworks, the top 10 configurations were selected based on internal validation performance, and 10 corresponding stacking-based extensions were additionally evaluated. The auxiliary forced-recall evaluation applied a recall-floor criterion of ≥0.995 to the same screening source configurations and their corresponding stacking extensions. Thus, 60 pipelines were evaluated in total: 20 screening, 20 balanced, and 20 auxiliary forced-recall pipelines. Reported metrics include recall, precision, F1-score, accuracy, area under the receiver operating characteristic curve (AUC), and area under the precision–recall curve (AUPRC). Precision denotes classification precision, i.e., positive predictive value, not analytical-method precision.

(a) External test performance of the 20 candidate pipelines under the screening framework

Rank	Model & Configuration[a]	Recall[b]	Precision[b]	F1[b]	Accuracy[b]	AUC[b]	AUPRC[b]
1	MajorityVote(k=2); NONE; none; QuantileNormal; β=0.5	0.797 (0.455–1.000; 0.636–1.000)	0.632 (0.500–0.758; 0.471–0.889)	0.693 (0.512–0.823; 0.572–0.890)	0.945 (0.920–0.966; 0.912–0.982)	0.969 (0.930–0.983; 0.952–0.995)	0.720 (0.654–0.767; 0.543–0.925)
2	MajorityVote(k=2); RUS_0.5; balanced; NONE; β=0.5	0.830 (0.636–1.000; 0.545–1.000)	0.669 (0.491–0.784; 0.500–0.909)	0.732 (0.616–0.820; 0.545–0.904)	0.951 (0.918–0.966; 0.920–0.985)	0.951 (0.873–0.986; 0.941–0.995)	0.668 (0.525–0.776; 0.467–0.930)
3	MajorityVote(k=2); RUS_1.0; none; NONE; β=0.5	0.815 (0.545–1.000; 0.545–1.000)	0.629 (0.528–0.778; 0.422–0.889)	0.701 (0.592–0.837; 0.536–0.857)	0.945 (0.927–0.971; 0.905–0.978)	0.951 (0.885–0.985; 0.937–0.993)	0.642 (0.521–0.774; 0.454–0.906)
4	MajorityVote(k=2); SMOTE; balanced; PowerYJ; β=0.5	0.836 (0.727–0.934; 0.545–1.000)	0.743 (0.590–0.833; 0.550–1.000)	0.783 (0.685–0.870; 0.632–0.957)	0.963 (0.940–0.978; 0.931–0.993)	0.982 (0.971–0.992; 0.961–0.998)	0.789 (0.671–0.879; 0.615–0.959)
5	MajorityVote(k=2); SMOTE; none; PowerYJ; β=0.5	0.836 (0.727–0.934; 0.545–1.000)	0.743 (0.590–0.833; 0.550–1.000)	0.783 (0.685–0.870; 0.632–0.957)	0.963 (0.940–0.978; 0.931–0.993)	0.982 (0.971–0.992; 0.961–0.998)	0.787 (0.671–0.879; 0.620–0.959)

6	MajorityVote(k=2); SMOTE_TOMEK; balanced; PowerYJ; β=0.5	0.836 (0.727–0.934; 0.545–1.000)	0.743 (0.590–0.833; 0.556–1.000)	0.783 (0.685–0.870; 0.632–0.957)	0.963 (0.940–0.978; 0.927–0.993)	0.982 (0.971–0.992; 0.962–0.998)	0.789 (0.671–0.879; 0.622–0.958)
7	MajorityVote(k=2); SMOTE_TOMEK; none; PowerYJ; β=0.5	0.836 (0.727–0.934; 0.545–1.000)	0.743 (0.590–0.833; 0.553–1.000)	0.783 (0.685–0.870; 0.628–0.957)	0.963 (0.940–0.978; 0.934–0.993)	0.982 (0.971–0.992; 0.961–0.998)	0.788 (0.671–0.879; 0.615–0.960)
8	MajorityVote(k=3); RUS_0.1; none; QuantileNormal; β=0.5	0.503 (0.273–0.909; 0.182–0.727)	0.709 (0.540–0.833; 0.400–1.000)	0.567 (0.393–0.809; 0.258–0.800)	0.943 (0.925–0.966; 0.912–0.971)	0.961 (0.915–0.984; 0.946–0.994)	0.694 (0.591–0.789; 0.493–0.903)
9	MajorityVote(k=3); RUS_0.7; balanced; NONE; β=1	0.852 (0.636–1.000; 0.727–1.000)	0.648 (0.512–0.764; 0.474–0.900)	0.729 (0.621–0.804; 0.561–0.909)	0.949 (0.923–0.964; 0.912–0.985)	0.974 (0.950–0.985; 0.949–0.995)	0.667 (0.553–0.737; 0.489–0.928)
10	MajorityVote(k=4); RUS_0.333; balanced; NONE; β=2	0.858 (0.702–1.000; 0.545–1.000)	0.643 (0.529–0.778; 0.462–0.861)	0.732 (0.631–0.837; 0.553–0.890)	0.950 (0.927–0.971; 0.912–0.982)	0.978 (0.967–0.987; 0.951–0.996)	0.699 (0.627–0.774; 0.518–0.936)
11	Stacking; NONE; none; QuantileNormal; β=0.5; τ=0.925	0.373 (0.157–0.661; 0.091–0.636)	0.823 (0.576–1.000; 0.382–1.000)	0.479 (0.253–0.645; 0.154–0.778)	0.940 (0.927–0.951; 0.916–0.971)	0.979 (0.975–0.981; 0.953–0.996)	0.780 (0.723–0.821; 0.593–0.956)
12	Stacking; RUS_0.1; none; QuantileNormal; β=0.5; τ=0.923	0.361 (0.157–0.934; 0.000–0.545)	0.853 (0.644–1.000; 0.000–1.000)	0.460 (0.269–0.774; 0.000–0.706)	0.940 (0.932–0.956; 0.920–0.964)	0.979 (0.974–0.983; 0.953–0.996)	0.785 (0.724–0.828; 0.601–0.956)
13	Stacking; RUS_0.333; balanced; NONE; β=2; τ=0.420	0.921 (0.727–1.000; 0.727–1.000)	0.549 (0.415–0.667; 0.407–0.773)	0.682 (0.586–0.769; 0.544–0.846)	0.930 (0.886–0.956; 0.890–0.971)	0.980 (0.968–0.987; 0.960–0.998)	0.799 (0.659–0.867; 0.618–0.981)
14	Stacking; RUS_0.5; balanced; NONE; β=0.5; τ=0.752	0.758 (0.314–1.000; 0.545–1.000)	0.625 (0.532–0.816; 0.423–0.852)	0.667 (0.441–0.794; 0.483–0.869)	0.942 (0.927–0.958; 0.898–0.978)	0.977 (0.964–0.987; 0.950–0.996)	0.751 (0.614–0.877; 0.554–0.967)

15	Stacking; RUS_0.7; balanced; NONE; β=1; τ=0.666	0.809 (0.545–1.000; 0.545–1.000)	0.565 (0.458–0.667; 0.405–0.809)	0.660 (0.534–0.749; 0.500–0.840)	0.934 (0.908–0.951; 0.890–0.971)	0.976 (0.964–0.987; 0.946–0.996)	0.726 (0.622–0.826; 0.526–0.960)
16	Stacking; RUS_1.0; none; NONE; β=0.5; τ=0.734	0.770 (0.248–1.000; 0.545–1.000)	0.593 (0.491–0.862; 0.416–0.818)	0.649 (0.335–0.741; 0.491–0.852)	0.936 (0.918–0.958; 0.898–0.975)	0.971 (0.933–0.986; 0.946–0.995)	0.708 (0.510–0.854; 0.540–0.960)
17	Stacking; SMOTE_TOMEK; balanced; PowerYJ; β=0.5; τ=0.973	0.321 (0.000–0.389; 0.091–0.636)	0.928 (0.000–1.000; 1.000–1.000)	0.473 (0.000–0.548; 0.167–0.778)	0.945 (0.920–0.949; 0.927–0.971)	0.979 (0.973–0.986; 0.953–0.996)	0.799 (0.760–0.863; 0.612–0.955)
18	Stacking; SMOTE_TOMEK; none; PowerYJ; β=0.5; τ=0.980	0.288 (0.000–0.389; 0.091–0.636)	0.850 (0.000–1.000; 1.000–1.000)	0.425 (0.000–0.539; 0.167–0.778)	0.942 (0.920–0.949; 0.927–0.971)	0.979 (0.974–0.986; 0.953–0.996)	0.799 (0.763–0.859; 0.610–0.955)
19	Stacking; SMOTE; balanced; PowerYJ; β=0.5; τ=0.973	0.321 (0.000–0.455; 0.091–0.636)	0.922 (0.000–1.000; 1.000–1.000)	0.471 (0.000–0.588; 0.167–0.778)	0.945 (0.920–0.949; 0.927–0.971)	0.979 (0.973–0.986; 0.952–0.996)	0.799 (0.760–0.863; 0.609–0.955)
20	Stacking; SMOTE; none; PowerYJ; β=0.5; τ=0.980	0.288 (0.000–0.389; 0.091–0.636)	0.850 (0.000–1.000; 1.000–1.000)	0.425 (0.000–0.539; 0.167–0.778)	0.942 (0.920–0.949; 0.927–0.971)	0.979 (0.974–0.986; 0.952–0.996)	0.799 (0.763–0.859; 0.605–0.955)

(b) External test performance of the 20 candidate pipelines under the balanced framework

Rank	Model & Configuration[a]	Recall[b]	Precision[b]	F1[b]	Accuracy[b]	AUC[b]	AUPRC[b]
1	LinearSVM_calibrated; SMOTE_TOMEK; balanced; Robust; β=1; τ=0.473	0.600 (0.455–0.777; 0.273–0.818)	0.814 (0.177–1.000; 0.600–1.000)	0.658 (0.281–0.795; 0.375–0.900)	0.926 (0.615–0.973; 0.927–0.985)	0.839 (0.753–0.929; 0.615–0.989)	0.703 (0.594–0.871; 0.409–0.933)
2	LinearSVM_calibrated; SMOTE_TOMEK; balanced; Robust; β=2; τ=0.459	0.645 (0.520–0.909; 0.364–	0.738 (0.084–1.000; 0.600–	0.627 (0.154–0.795; 0.396–	0.873 (0.194–0.973; 0.927–	0.839 (0.753–0.929; 0.616–	0.703 (0.594–0.871; 0.408–

		0.909)	1.000)	0.900)	0.985)	0.991)	0.933)
3	LinearSVM_calibrated; SMOTE_TOMEK; none; Robust; β=1; τ=0.473	0.600 (0.455–0.777; 0.273–0.818)	0.814 (0.181–1.000; 0.600–1.000)	0.658 (0.286–0.795; 0.375–0.900)	0.926 (0.620–0.973; 0.927–0.985)	0.839 (0.753–0.929; 0.614–0.990)	0.703 (0.595–0.871; 0.412–0.931)
4	LinearSVM_calibrated; SMOTE_TOMEK; none; Robust; β=2; τ=0.459	0.645 (0.520–0.909; 0.364–0.909)	0.738 (0.084–1.000; 0.600–1.000)	0.628 (0.154–0.795; 0.408–0.900)	0.873 (0.194–0.973; 0.927–0.985)	0.839 (0.753–0.929; 0.613–0.990)	0.703 (0.595–0.871; 0.415–0.936)
5	MajorityVote(k=2); RUS_0.5; balanced; NONE; β=0.5	0.830 (0.636–1.000; 0.545–1.000)	0.669 (0.491–0.784; 0.500–0.913)	0.732 (0.616–0.820; 0.549–0.909)	0.951 (0.918–0.966; 0.920–0.985)	0.951 (0.873–0.986; 0.940–0.995)	0.668 (0.525–0.776; 0.468–0.929)
6	MajorityVote(k=2); RUS_1.0; none; NONE; β=0.5	0.815 (0.545–1.000; 0.545–1.000)	0.629 (0.528–0.778; 0.422–0.894)	0.701 (0.592–0.837; 0.536–0.852)	0.945 (0.927–0.971; 0.905–0.978)	0.951 (0.885–0.985; 0.937–0.993)	0.642 (0.521–0.774; 0.457–0.909)
7	MajorityVote(k=2); SMOTE; none; Robust; β=0.5	0.812 (0.636–1.000; 0.545–1.000)	0.695 (0.581–0.784; 0.500–0.917)	0.742 (0.667–0.833; 0.566–0.909)	0.955 (0.940–0.971; 0.920–0.985)	0.966 (0.885–0.989; 0.955–0.998)	0.778 (0.673–0.865; 0.596–0.960)
8	MajorityVote(k=3); RUS_0.1; none; QuantileNormal; β=0.5	0.503 (0.273–0.909; 0.182–0.727)	0.709 (0.540–0.833; 0.400–1.000)	0.567 (0.393–0.809; 0.258–0.800)	0.943 (0.925–0.966; 0.912–0.971)	0.961 (0.915–0.984; 0.947–0.993)	0.694 (0.591–0.789; 0.495–0.904)
9	MajorityVote(k=4); RUS_0.333; balanced; NONE; β=2	0.858 (0.702–1.000; 0.545–1.000)	0.643 (0.529–0.778; 0.467–0.861)	0.732 (0.631–0.837; 0.549–0.900)	0.950 (0.927–0.971; 0.912–0.985)	0.978 (0.967–0.987; 0.951–0.996)	0.699 (0.627–0.774; 0.517–0.938)
10	SoftVote; SMOTE; none; Robust; β=1; τ=0.352	0.839 (0.636–1.000; 0.727–1.000)	0.698 (0.517–0.875; 0.500–0.958)	0.750 (0.667–0.837; 0.594–0.917)	0.955 (0.925–0.971; 0.920–0.985)	0.987 (0.982–0.990; 0.967–0.999)	0.886 (0.855–0.903; 0.749–0.992)
11	Stacking; RUS_0.1; none; QuantileNormal;	0.361 (0.157–0.934; 0.000–	0.853 (0.644–1.000; 0.000–	0.460 (0.269–0.774; 0.000–	0.940 (0.932–0.956; 0.920–	0.979 (0.974–0.983; 0.953–	0.785 (0.724–0.828; 0.597–

	β=0.5; τ=0.923	0.545)	1.000)	0.706)	0.964)	0.996)	0.959)
12	Stacking; RUS_0.333; balanced; NONE; β=2; τ=0.420	0.921 (0.727–1.000; 0.727–1.000)	0.549 (0.415–0.667; 0.408–0.769)	0.682 (0.586–0.769; 0.545–0.846)	0.930 (0.886–0.956; 0.890–0.971)	0.980 (0.968–0.987; 0.959–0.998)	0.799 (0.659–0.867; 0.617–0.979)
13	Stacking; RUS_0.5; balanced; NONE; β=0.5; τ=0.752	0.758 (0.314–1.000; 0.545–1.000)	0.625 (0.532–0.816; 0.420–0.861)	0.667 (0.441–0.794; 0.483–0.863)	0.942 (0.927–0.958; 0.898–0.978)	0.977 (0.964–0.987; 0.951–0.997)	0.751 (0.614–0.877; 0.553–0.969)
14	Stacking; RUS_1.0; none; NONE; β=0.5; τ=0.734	0.770 (0.248–1.000; 0.545–1.000)	0.593 (0.491–0.862; 0.420–0.800)	0.649 (0.335–0.741; 0.491–0.858)	0.936 (0.918–0.958; 0.898–0.975)	0.971 (0.933–0.986; 0.946–0.995)	0.708 (0.510–0.854; 0.543–0.963)
15	Stacking; SMOTE_TOMEK; balanced; Robust; β=1; τ=0.623	0.833 (0.545–1.000; 0.727–1.000)	0.681 (0.536–0.862; 0.500–0.909)	0.736 (0.641–0.809; 0.570–0.917)	0.952 (0.930–0.966; 0.920–0.985)	0.986 (0.979–0.991; 0.967–0.999)	0.868 (0.813–0.906; 0.721–0.986)
16	Stacking; SMOTE_TOMEK; balanced; Robust; β=2; τ=0.374	0.976 (0.818–1.000; 1.000–1.000)	0.547 (0.440–0.724; 0.415–0.733)	0.696 (0.611–0.787; 0.579–0.846)	0.930 (0.898–0.964; 0.887–0.971)	0.986 (0.979–0.991; 0.967–0.999)	0.868 (0.813–0.906; 0.721–0.986)
17	Stacking; SMOTE_TOMEK; none; Robust; β=1; τ=0.590	0.833 (0.636–1.000; 0.545–1.000)	0.672 (0.513–0.784; 0.500–0.909)	0.733 (0.661–0.833; 0.544–0.909)	0.951 (0.923–0.971; 0.920–0.985)	0.985 (0.977–0.989; 0.964–0.999)	0.864 (0.818–0.894; 0.717–0.985)
18	Stacking; SMOTE_TOMEK; none; Robust; β=2; τ=0.357	0.955 (0.818–1.000; 1.000–1.000)	0.542 (0.440–0.714; 0.390–0.710)	0.684 (0.611–0.787; 0.537–0.831)	0.927 (0.898–0.960; 0.876–0.967)	0.985 (0.977–0.989; 0.964–0.999)	0.864 (0.818–0.894; 0.718–0.985)
19	Stacking; SMOTE; none; Robust; β=0.5; τ=0.961	0.488 (0.182–0.843; 0.182–0.727)	0.917 (0.685–1.000; 0.577–1.000)	0.600 (0.308–0.772; 0.308–0.842)	0.952 (0.934–0.966; 0.927–0.978)	0.985 (0.981–0.988; 0.964–0.999)	0.867 (0.834–0.891; 0.718–0.985)
20	Stacking; SMOTE; none; Robust; β=1; τ=0.606	0.842 (0.636–1.000; 0.636–	0.661 (0.550–0.778; 0.483–	0.730 (0.667–0.800; 0.561–	0.950 (0.934–0.964; 0.916–	0.985 (0.981–0.988; 0.964–	0.867 (0.834–0.891; 0.722–

		1.000)	0.917)	0.890)	0.982)	0.999)	0.985)

(c) External test performance of the 20 candidate pipelines under the forced-recall variant

Rank	Model & Configuration[a]	Recall[b]	Precision[b]	F1[b]	Accuracy[b]	AUC[b]	AUPRC[b]
1	MajorityVote(k=2); NONE; none; QuantileNormal; β=0.5	1.000 (1.000–1.000; 1.000–1.000)	0.080 (0.080–0.080; 0.080–0.080)	0.149 (0.149–0.149; 0.149–0.149)	0.080 (0.080–0.080; 0.080–0.080)	0.899 (0.713–0.947; 0.883–0.948)	0.336 (0.133–0.451; 0.272–0.458)
2	MajorityVote(k=2); RUS_0.5; balanced; NONE; β=0.5	1.000 (1.000–1.000; 1.000–1.000)	0.080 (0.080–0.080; 0.080–0.080)	0.149 (0.149–0.149; 0.149–0.149)	0.080 (0.080–0.080; 0.080–0.080)	0.735 (0.532–0.885; 0.675–0.819)	0.164 (0.086–0.291; 0.124–0.195)
3	MajorityVote(k=2); RUS_1.0; none; NONE; β=0.5	1.000 (1.000–1.000; 1.000–1.000)	0.080 (0.080–0.080; 0.080–0.080)	0.149 (0.149–0.149; 0.149–0.149)	0.080 (0.080–0.080; 0.080–0.080)	0.745 (0.551–0.906; 0.655–0.823)	0.164 (0.089–0.317; 0.119–0.198)
4	MajorityVote(k=2); SMOTE; balanced; PowerYJ; β=0.5	1.000 (1.000–1.000; 1.000–1.000)	0.080 (0.080–0.080; 0.080–0.080)	0.149 (0.149–0.149; 0.149–0.149)	0.080 (0.080–0.080; 0.080–0.080)	0.882 (0.666–0.936; 0.888–0.952)	0.312 (0.116–0.407; 0.281–0.478)
5	MajorityVote(k=2); SMOTE; none; PowerYJ; β=0.5	1.000 (1.000–1.000; 1.000–1.000)	0.080 (0.080–0.080; 0.080–0.080)	0.149 (0.149–0.149; 0.149–0.149)	0.080 (0.080–0.080; 0.080–0.080)	0.881 (0.640–0.936; 0.881–0.946)	0.312 (0.108–0.407; 0.269–0.449)
6	MajorityVote(k=2); SMOTE_TOMEK; balanced; PowerYJ; β=0.5	1.000 (1.000–1.000; 1.000–1.000)	0.080 (0.080–0.080; 0.080–0.080)	0.149 (0.149–0.149; 0.149–0.149)	0.080 (0.080–0.080; 0.080–0.080)	0.882 (0.666–0.936; 0.886–0.950)	0.312 (0.116–0.407; 0.277–0.468)
7	MajorityVote(k=2); SMOTE_TOMEK; none; PowerYJ; β=0.5	1.000 (1.000–1.000; 1.000–1.000)	0.080 (0.080–0.080; 0.080–0.080)	0.149 (0.149–0.149; 0.149–0.149)	0.080 (0.080–0.080; 0.080–0.080)	0.881 (0.640–0.936; 0.879–0.946)	0.312 (0.108–0.407; 0.265–0.449)
8	MajorityVote(k=3); RUS_0.1; none; QuantileNormal;	1.000 (1.000–1.000;	0.087 (0.080–0.097;	0.160 (0.149–0.177;	0.154 (0.080–0.256;	0.903 (0.741–0.946;	0.338 (0.146–0.445;

	β=0.5	1.000–1.000)	0.083–0.090)	0.153–0.166)	0.113–0.193)	0.889–0.950)	0.282–0.468)
9	MajorityVote(k=3); RUS_0.7; balanced; NONE; β=1	1.000 (1.000–1.000; 1.000–1.000)	0.080 (0.080–0.081; 0.080–0.080)	0.149 (0.149–0.149; 0.149–0.149)	0.081 (0.080–0.082; 0.080–0.080)	0.738 (0.524–0.886; 0.660–0.807)	0.162 (0.084–0.304; 0.116–0.186)
10	MajorityVote(k=4); RUS_0.333; balanced; NONE; β=2	1.000 (1.000–1.000; 1.000–1.000)	0.083 (0.080–0.108; 0.080–0.080)	0.153 (0.149–0.196; 0.149–0.149)	0.106 (0.080–0.337; 0.080–0.080)	0.786 (0.529–0.934; 0.747–0.885)	0.207 (0.085–0.419; 0.154–0.277)
11	Stacking; NONE; none; QuantileNormal; β=0.5; τ=0.155	1.000 (1.000–1.000; 1.000–1.000)	0.296 (0.137–0.407; 0.275–0.458)	0.447 (0.242–0.579; 0.431–0.629)	0.766 (0.496–0.883; 0.788–0.905)	0.979 (0.975–0.981; 0.954–0.996)	0.780 (0.723–0.821; 0.598–0.956)
12	Stacking; RUS_0.1; none; QuantileNormal; β=0.5; τ=0.164	1.000 (1.000–1.000; 1.000–1.000)	0.291 (0.134–0.412; 0.275–0.468)	0.442 (0.236–0.583; 0.431–0.638)	0.760 (0.474–0.885; 0.788–0.909)	0.979 (0.974–0.983; 0.954–0.996)	0.785 (0.724–0.828; 0.599–0.957)
13	Stacking; RUS_0.333; balanced; NONE; β=2; τ=0.135	1.000 (1.000–1.000; 1.000–1.000)	0.150 (0.083–0.238; 0.126–0.168)	0.258 (0.153–0.383; 0.223–0.288)	0.495 (0.114–0.736; 0.442–0.602)	0.980 (0.968–0.987; 0.959–0.998)	0.799 (0.659–0.867; 0.620–0.981)
14	Stacking; RUS_0.5; balanced; NONE; β=0.500; τ=0.120	1.000 (1.000–1.000; 1.000–1.000)	0.144 (0.082–0.236; 0.118–0.153)	0.249 (0.151–0.382; 0.210–0.265)	0.473 (0.096–0.740; 0.398–0.555)	0.977 (0.964–0.987; 0.951–0.996)	0.751 (0.614–0.877; 0.555–0.969)
15	Stacking; RUS_0.7; balanced; NONE; β=1; τ=0.113	1.000 (1.000–1.000; 1.000–1.000)	0.134 (0.082–0.187; 0.122–0.162)	0.234 (0.152–0.316; 0.218–0.279)	0.434 (0.102–0.652; 0.423–0.584)	0.976 (0.964–0.987; 0.946–0.995)	0.726 (0.622–0.826; 0.527–0.961)
16	Stacking; RUS_1.0; none; NONE; β=0.5; τ=0.109	0.997 (0.975–1.000; 1.000–1.000)	0.133 (0.082–0.193; 0.114–0.147)	0.233 (0.151–0.324; 0.205–0.256)	0.442 (0.096–0.664; 0.376–0.533)	0.971 (0.933–0.986; 0.946–0.996)	0.708 (0.510–0.854; 0.542–0.962)
17	Stacking; SMOTE_TOMEK; balanced; PowerYJ;	1.000 (1.000–1.000;	0.230 (0.094–0.401;	0.361 (0.172–0.573;	0.631 (0.224–0.880;	0.979 (0.973–0.986;	0.799 (0.760–0.863;

	β=0.5; τ=0.117	1.000–1.000)	0.172–0.259)	0.293–0.411)	0.613–0.770)	0.952–0.996)	0.609–0.956)
18	Stacking; SMOTE_TOMEK; none; PowerYJ; β=0.5; τ=0.116	1.000 (1.000–1.000; 1.000–1.000)	0.229 (0.094–0.401; 0.173–0.259)	0.360 (0.172–0.573; 0.295–0.411)	0.628 (0.230–0.880; 0.617–0.770)	0.979 (0.974–0.986; 0.953–0.996)	0.799 (0.763–0.859; 0.607–0.955)
19	Stacking; SMOTE; balanced; PowerYJ; β=0.5; τ=0.117	1.000 (1.000–1.000; 1.000–1.000)	0.230 (0.094–0.401; 0.172–0.259)	0.361 (0.172–0.573; 0.293–0.411)	0.630 (0.224–0.880; 0.613–0.770)	0.979 (0.973–0.986; 0.952–0.996)	0.799 (0.760–0.863; 0.605–0.955)
20	Stacking; SMOTE; none; PowerYJ; β=0.5; τ=0.116	1.000 (1.000–1.000; 1.000–1.000)	0.229 (0.094–0.401; 0.175–0.256)	0.359 (0.172–0.573; 0.297–0.407)	0.627 (0.230–0.880; 0.620–0.766)	0.979 (0.974–0.986; 0.952–0.996)	0.799 (0.763–0.859; 0.609–0.955)

a Model configurations are reported in the following order: algorithm; resampling strategy; class-weighting scheme; feature scaling method; and, where applicable, the Fβ parameter (β) and operating decision threshold (τ).

b Performance metrics are reported as mean values with 95% seed CIs and bootstrap interval summaries. Seed CIs represent the 2.5th–97.5th percentile range across 30 independent repetitions. Bootstrap intervals summarize the median lower and upper bounds of seed-specific 95% CIs obtained from 5,000 class-stratified resamples per repetition.

Table S12. External Hold-Out Check for Confirmed Substances Absent from the Training-Positive Subset.

Anonymized sample ID	All confirmed detected substances	Training-uncovered substance(s)	Co-occurring training-represented substance(s)	Predicted positive-class probability	Model classification
S622	19-NA; 19-NE	19-NE	19-NA	0.990	Flagged positive
S623	19-NA; 19-NE	19-NE	19-NA	0.988	Flagged positive
S637	Zeranol-M; Zeranol	Zeranol-M; Zeranol	—	0.959	Flagged positive
S655	19-NE; 19-NA	19-NE	19-NA	0.897	Flagged positive
S658	Mibolerone	Mibolerone	—	0.810	Flagged positive

Note. Analyte names and abbreviations follow the nomenclature used in Table S1. Training-uncovered substances indicate confirmed substances present in the external-positive subset but absent from the training-positive subset. Predicted probabilities were generated by the fixed Rank 1 model; model classifications were assigned using the predefined operating threshold.

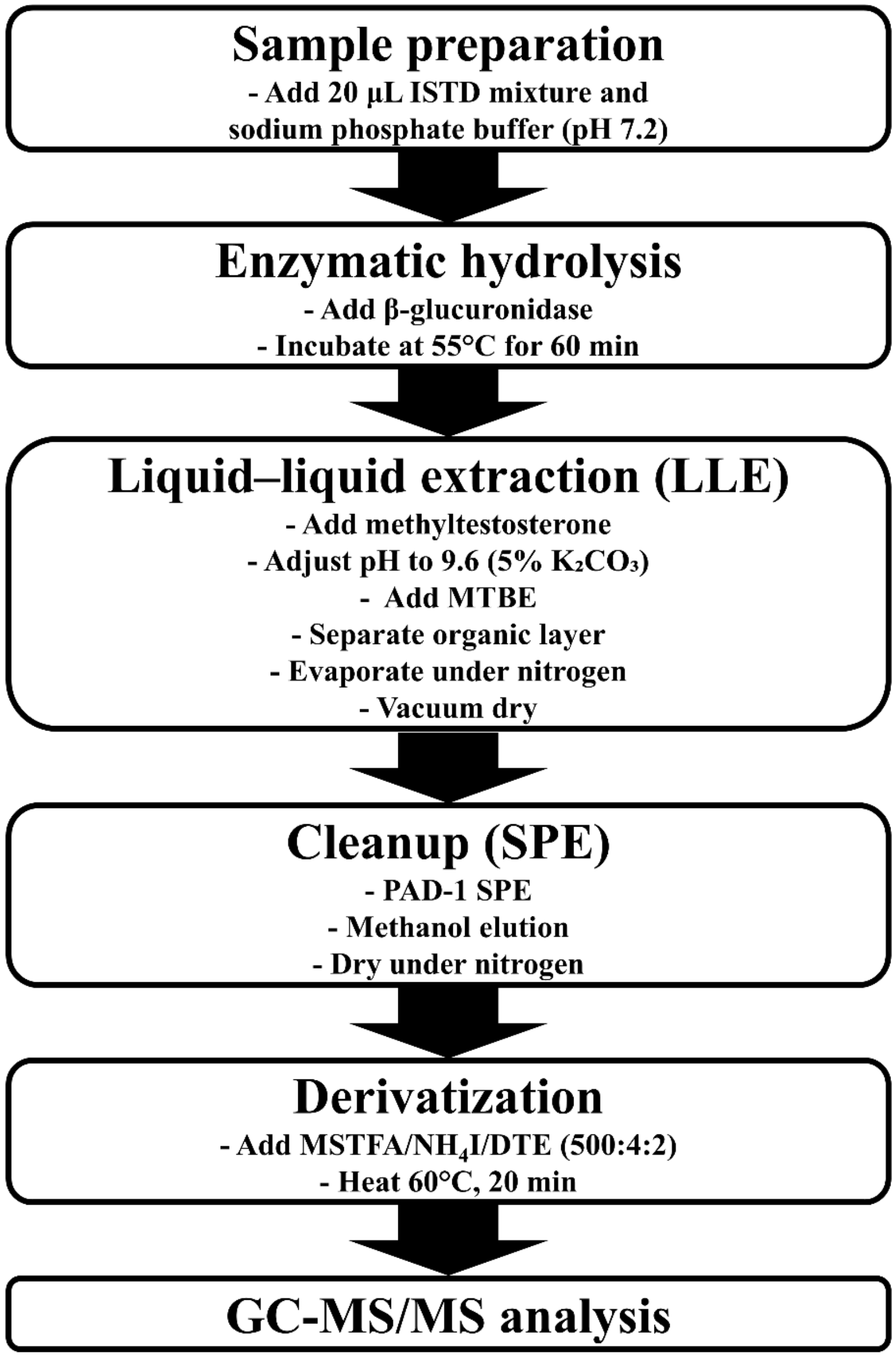

Figure S1. Schematic workflow for urine sample preparation and GC–MS/MS analysis of prohibited substances. The scheme summarizes the major analytical steps used in this study, including internal-standard addition, enzymatic hydrolysis, liquid–liquid extraction, cleanup, derivatization, and final GC–MS/MS analysis.

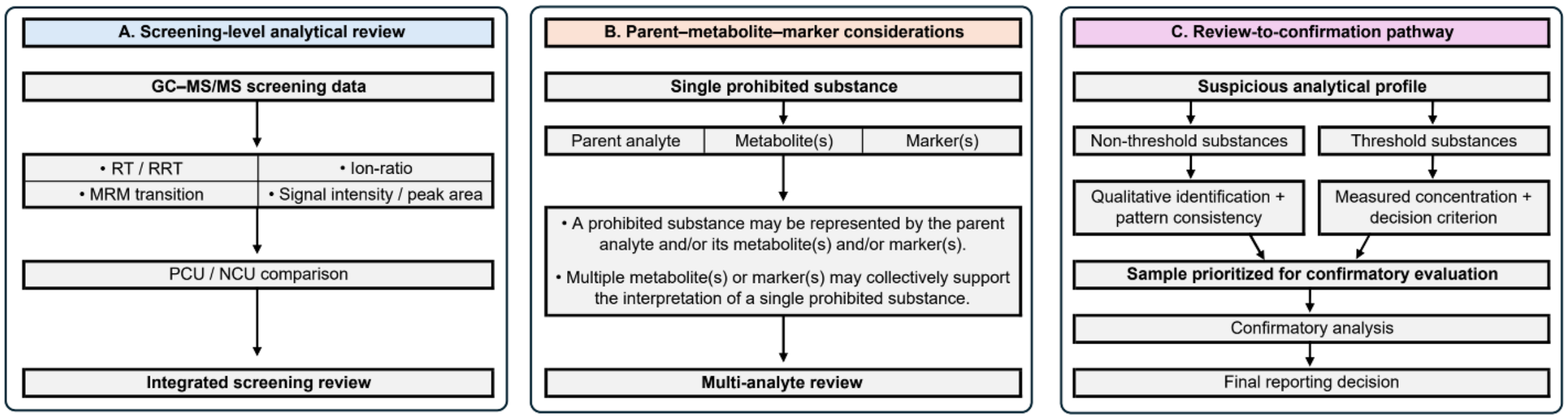

Figure S2. General anti-doping analytical review workflow. The schematic emphasizes that the positive/negative labels used in this study were not assigned from a single analytical peak or threshold-based rule alone, but were derived through a structured multi-step anti-doping review process integrating screening-level analytical features, parent–metabolite–marker considerations, and confirmatory evaluation as applicable. This workflow highlights that the training labels used for model development reflected operationally adjudicated sample-level outcomes and therefore provide an appropriate basis for evaluating machine-learning support at the screening stage.

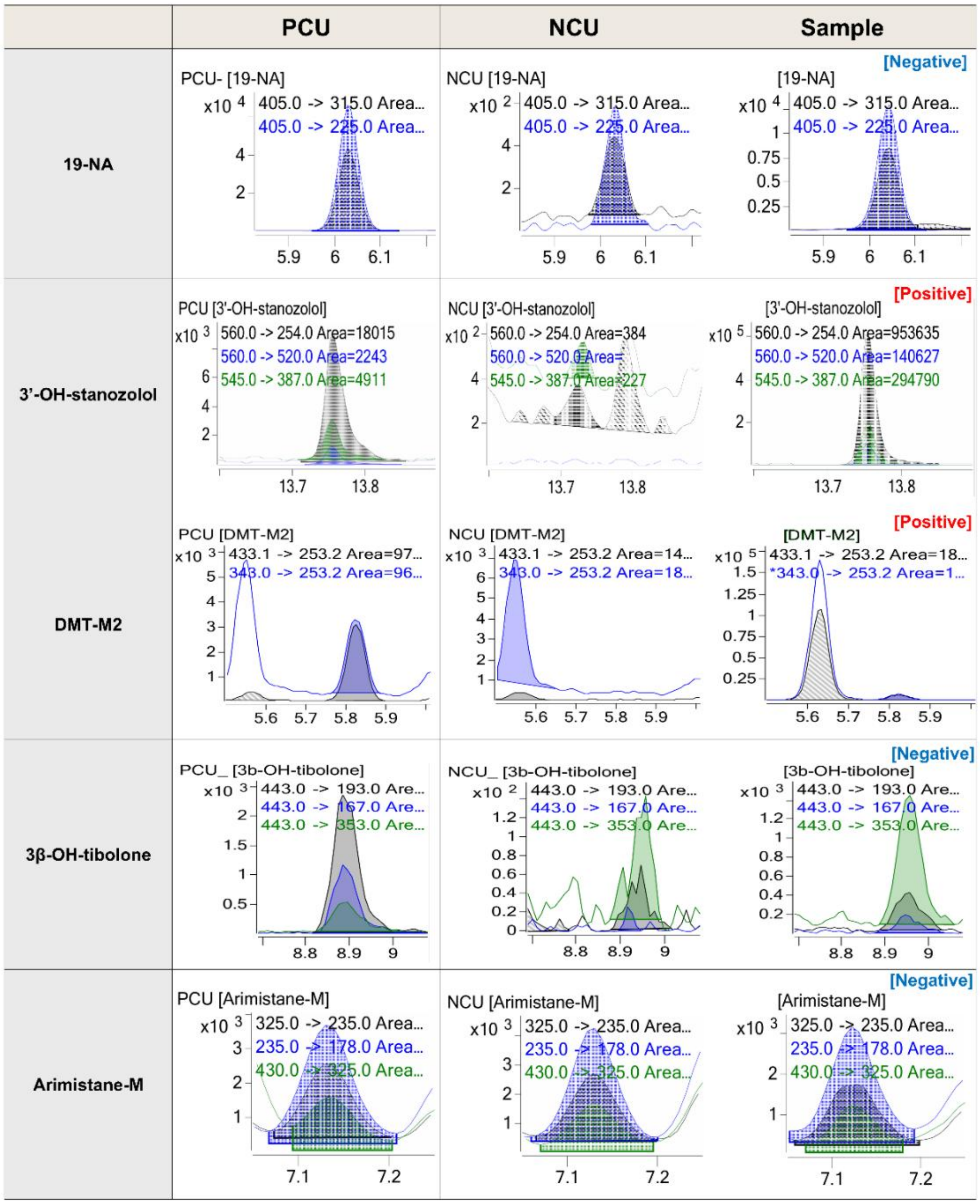

Figure S3. Representative raw chromatograms illustrating diverse interpretive scenarios encountered during routine manual review in anti-doping analysis. Selected analytes, including 19-NA, 3′-OH-stanozolol, DMT-M2, 3β-OH-tibolone, and Arimistane-M, are shown as three-way comparisons of positive control urine (PCU), negative control urine (NCU), and representative test samples. The examples show that chromatographic patterns differ across analytes: in some cases, peak-like or background signals are also observed in negative controls or negative samples, whereas in others positive samples show clearer and more concordant signal patterns. Accordingly, classification was not determined solely by the presence of an apparent peak or by signal intensity alone, but required integrated evaluation of RT consistency, ion-ratio agreement among monitored MRM transitions, comparison with control-derived chromatographic patterns, and consideration of background or interfering signals. These scenarios illustrate that routine anti-doping review cannot always be reduced to a single prominent peak or a uniform threshold-based rule.

Figure S4. Normalized intensity distributions for all analyte-specific input features. Peak intensities were transformed as log10(area + 1) and min–max scaled to 0–1 within each analyte. Blue points indicate negative-labeled samples, orange points indicate the full positive-labeled sample class, and green points, when present, indicate the target-analyte-positive subset within the positive class. Gray boxplots summarize the negative and full positive classes. Because class labels were assigned at the sample level, orange points in an individual analyte panel do not necessarily indicate that the displayed analyte was the confirmed positive analyte for every positive-labeled sample. These distributions are intended to illustrate exploratory within-analyte response patterns, including class separation and overlap, and should not be interpreted as absolute signal magnitudes across analytes or as final compound-specific reportable findings in negative-labeled samples.

The following eight pages comprise Figure S4.

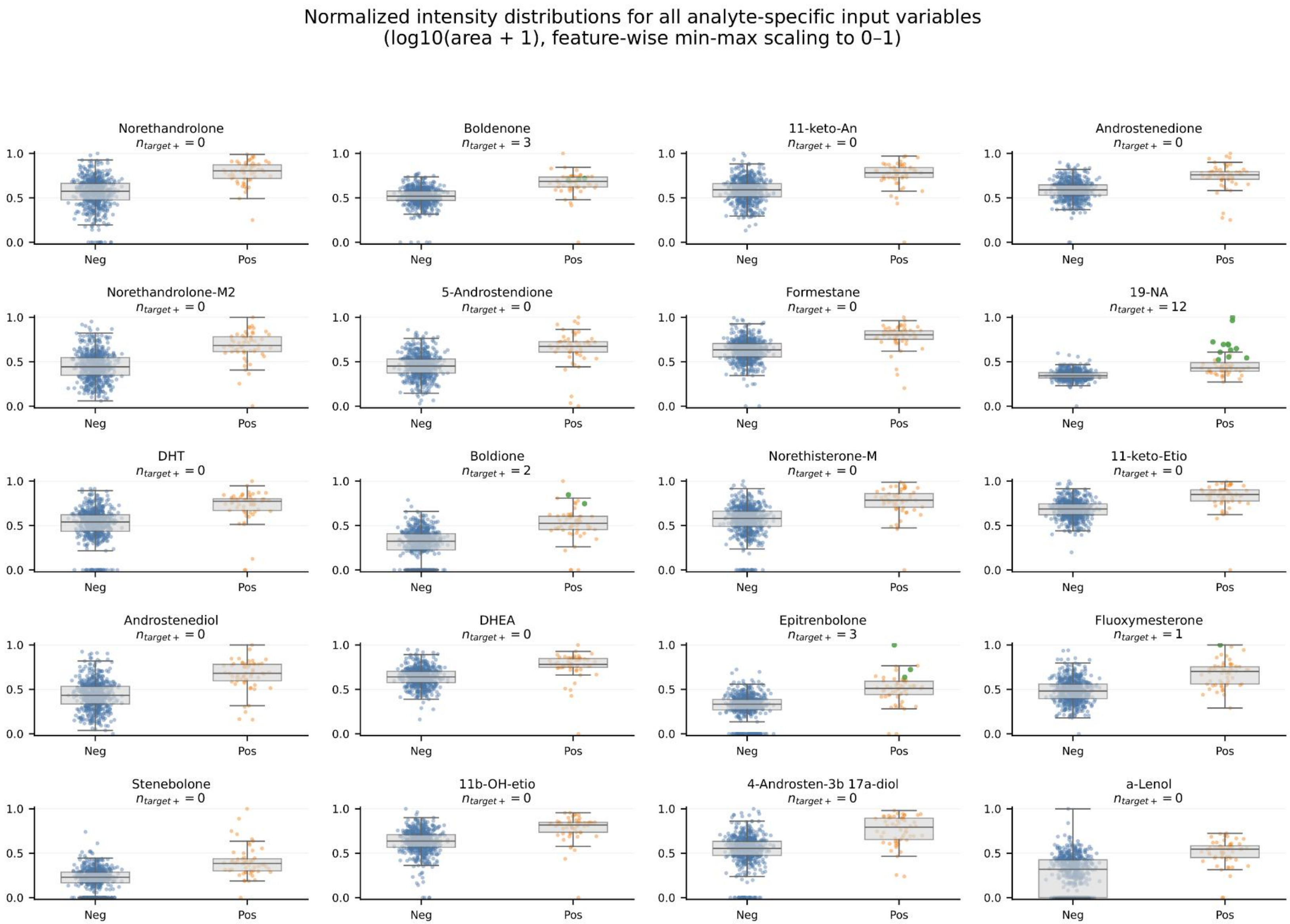

Figure S4 (page 1 of 8)

Normalized intensity distributions for all analyte-specific input variables
(log10(area + 1), feature-wise min-max scaling to 0–1)

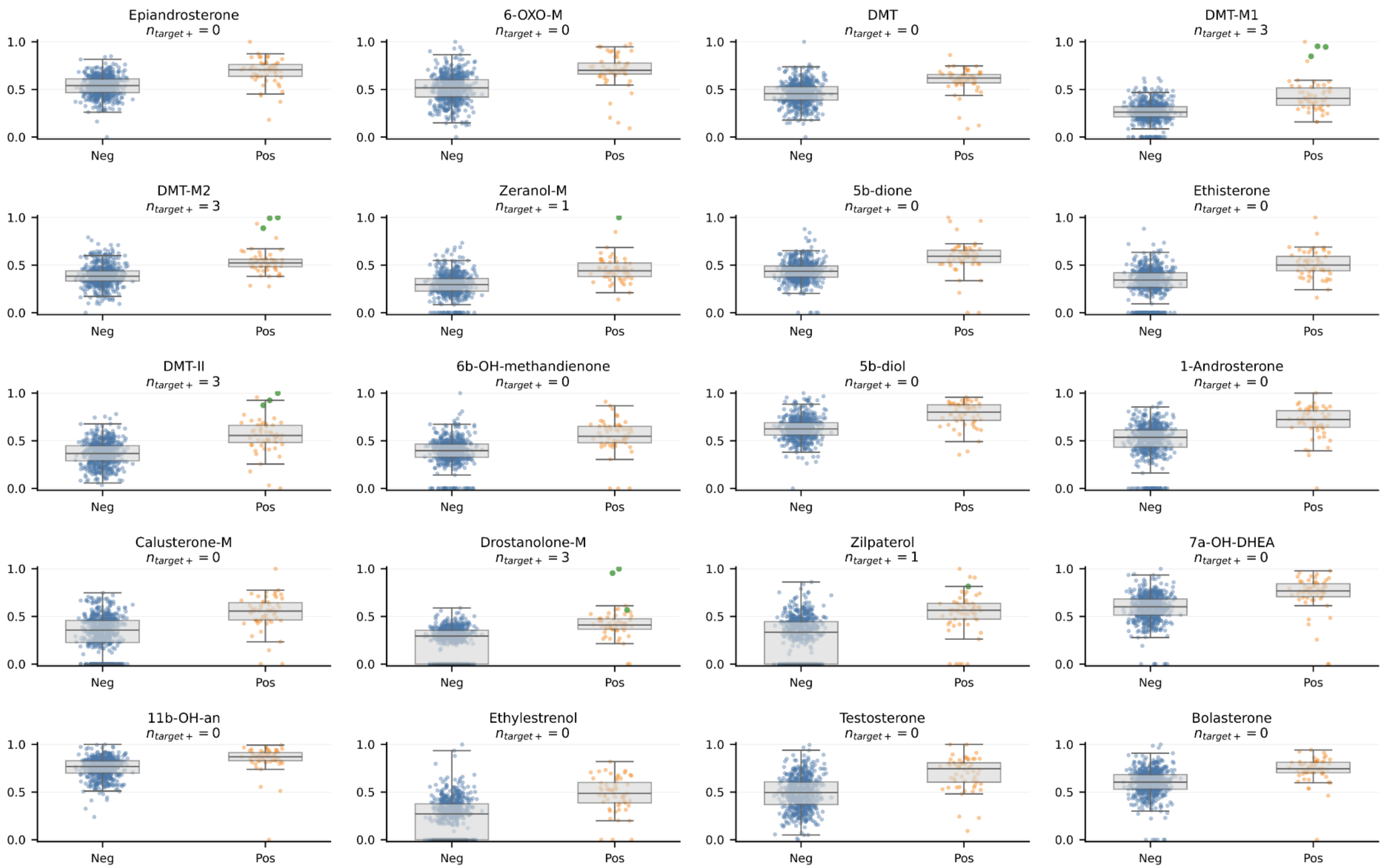

Figure S4 (page 2 of 8)

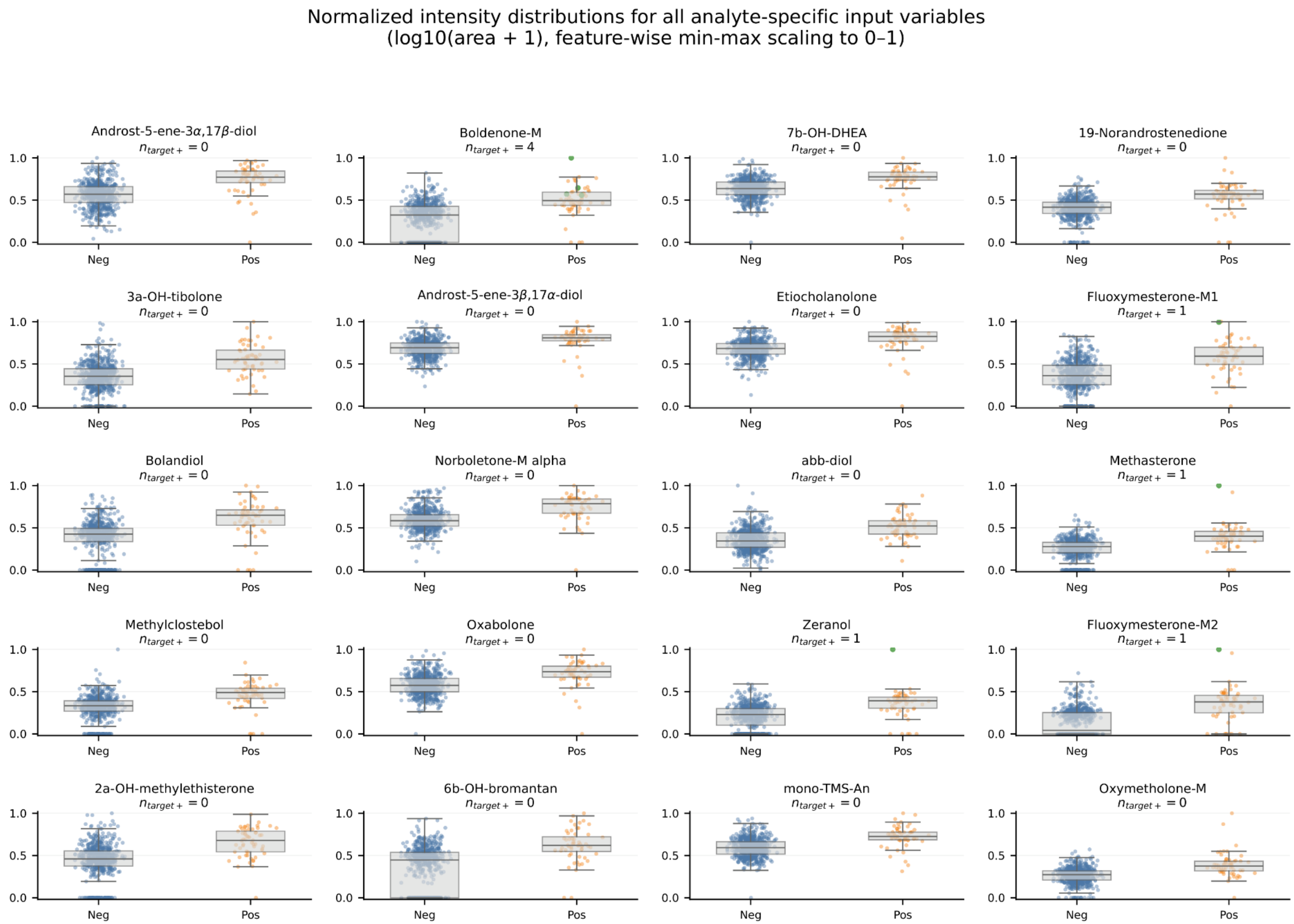

Figure S4 (page 3 of 8)

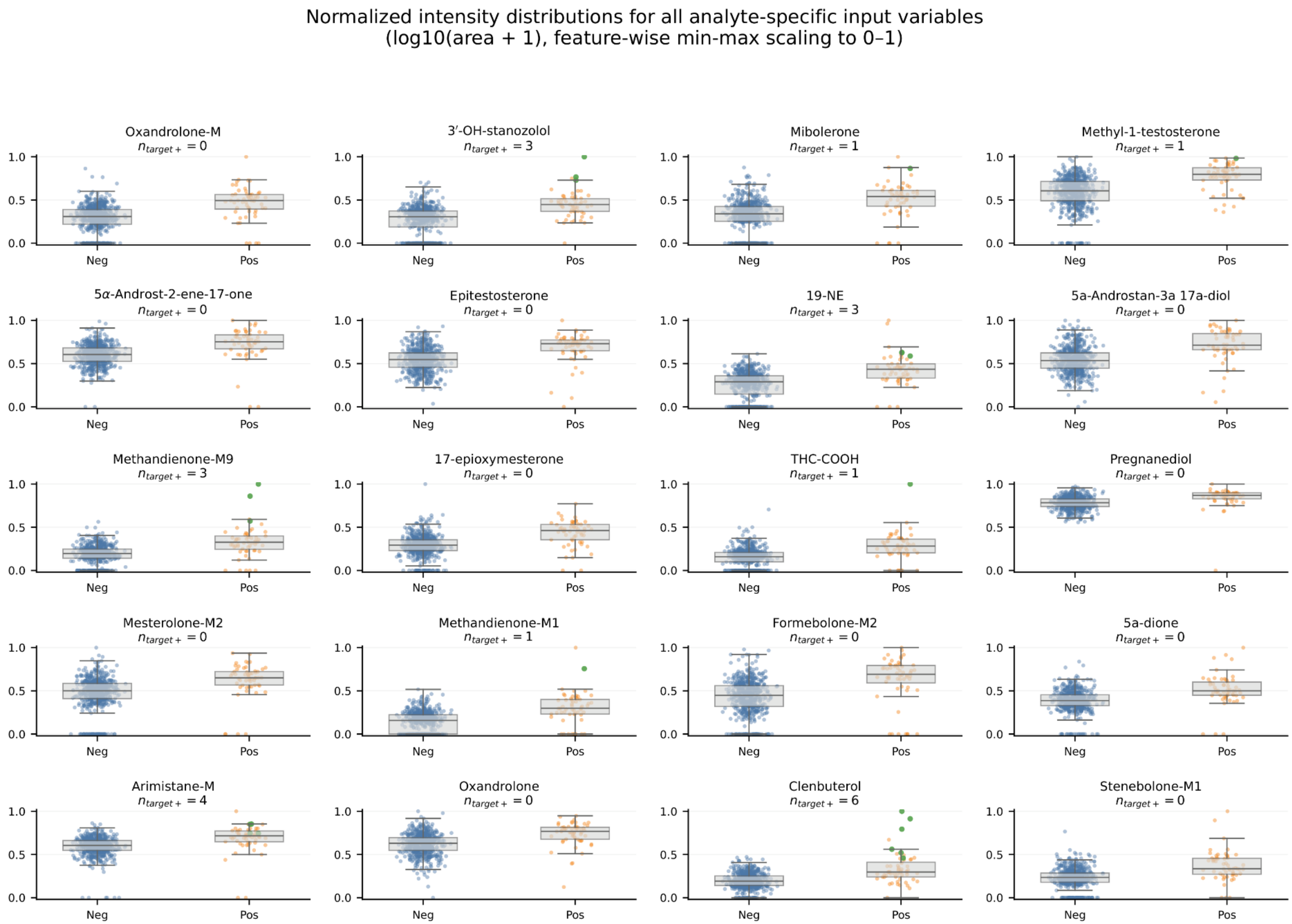

Figure S4 (page 4 of 8)

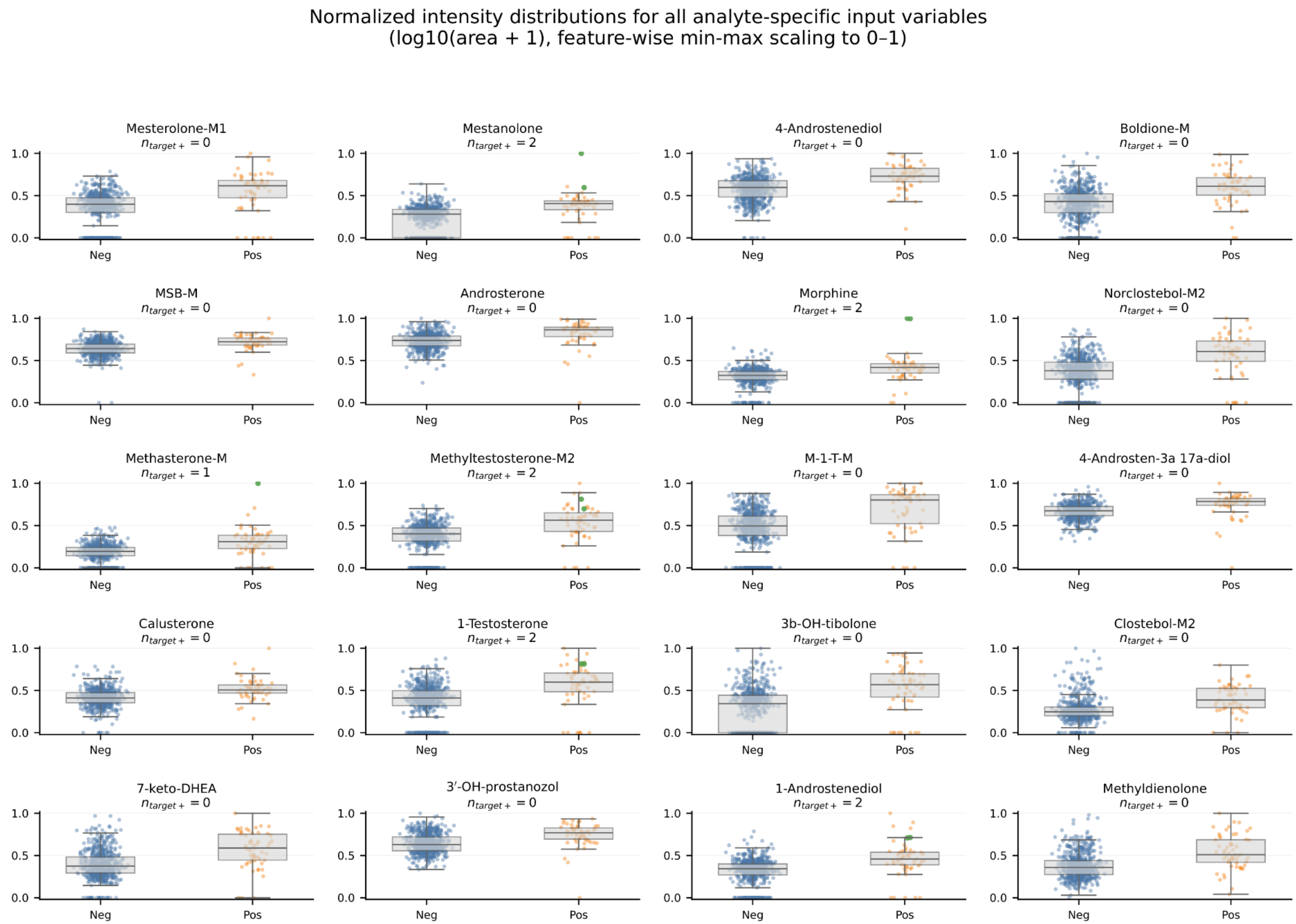

Figure S4 (page 5 of 8)

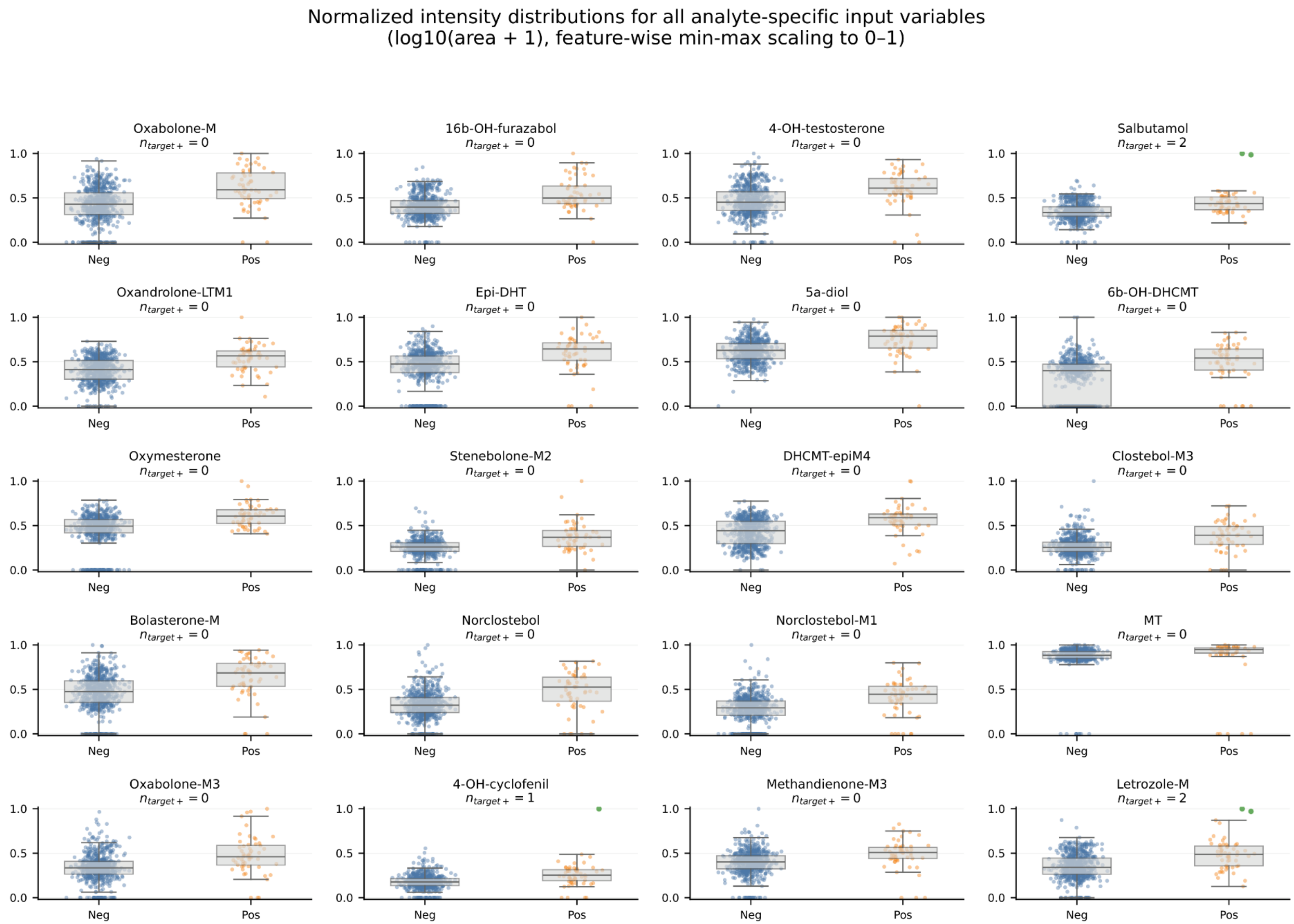

Figure S4 (page 6 of 8)

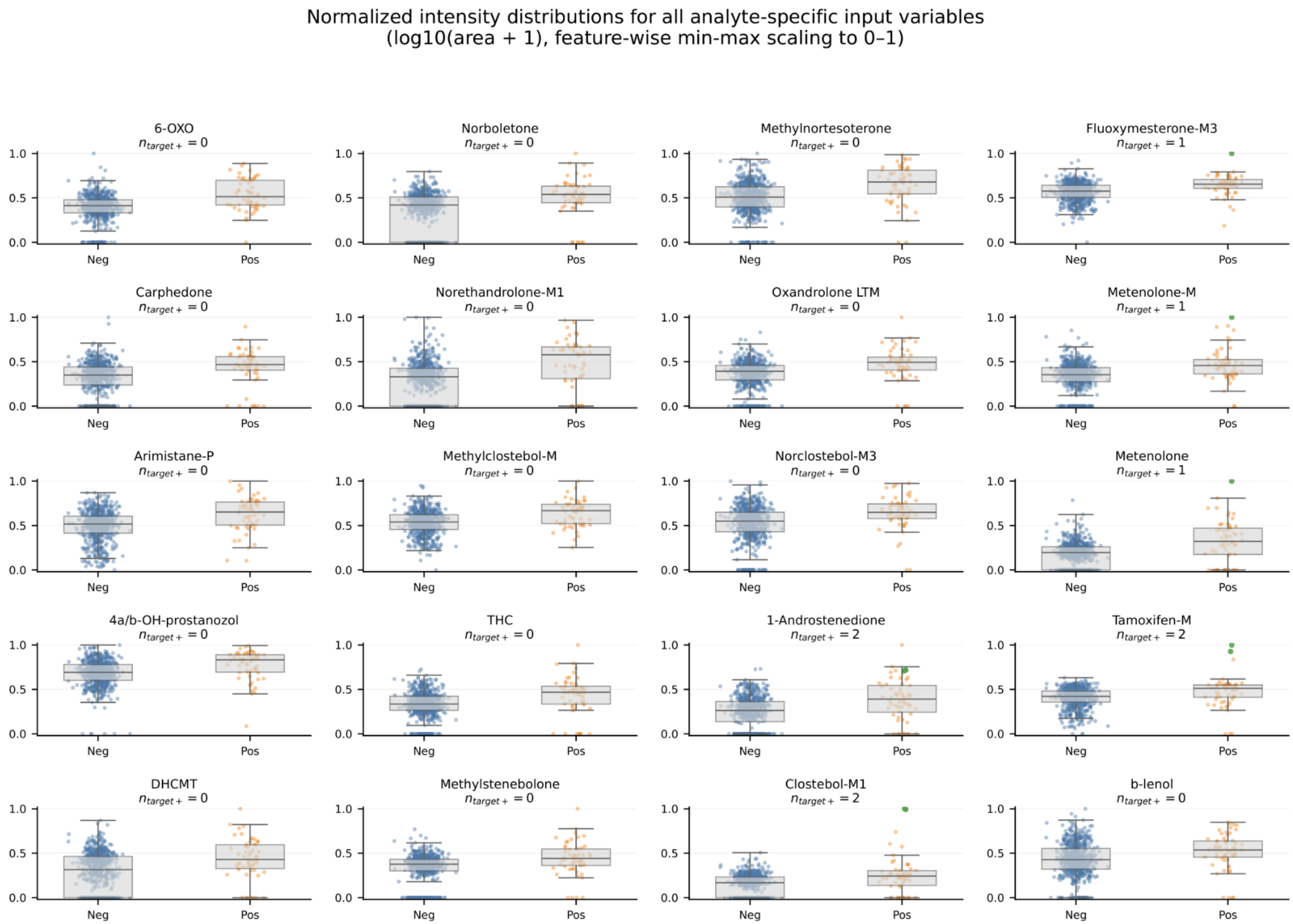

Figure S4 (page 7 of 8)

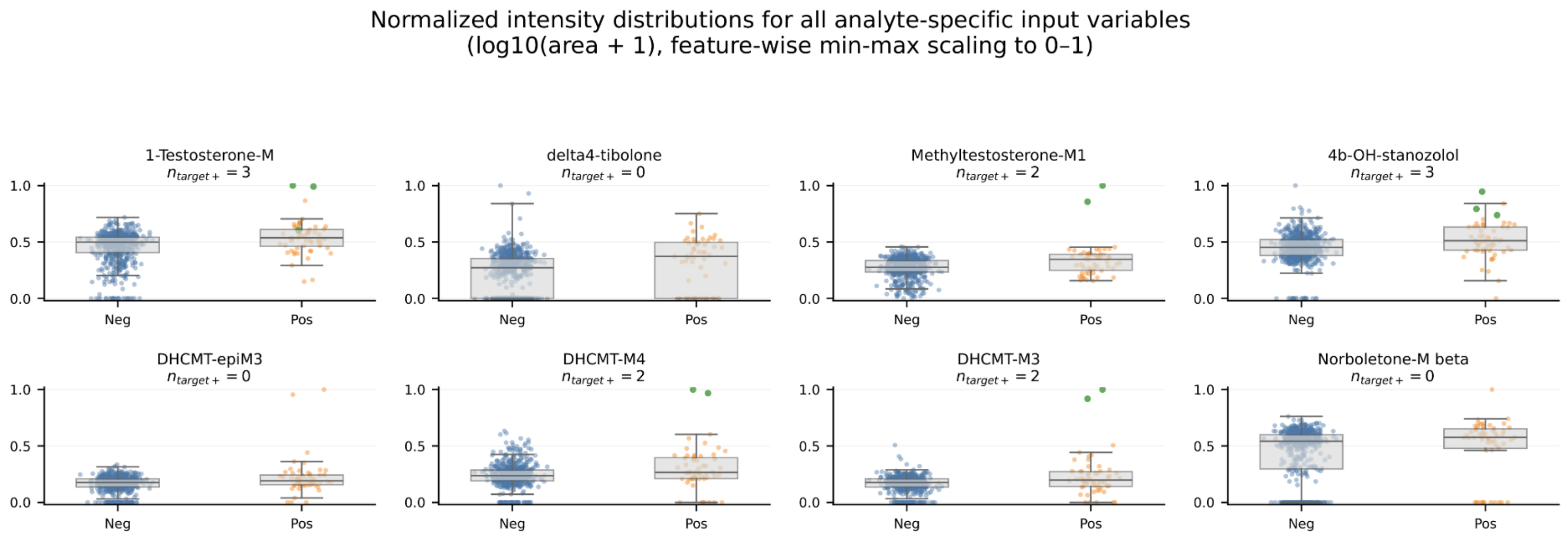

Figure S4 (page 8 of 8)

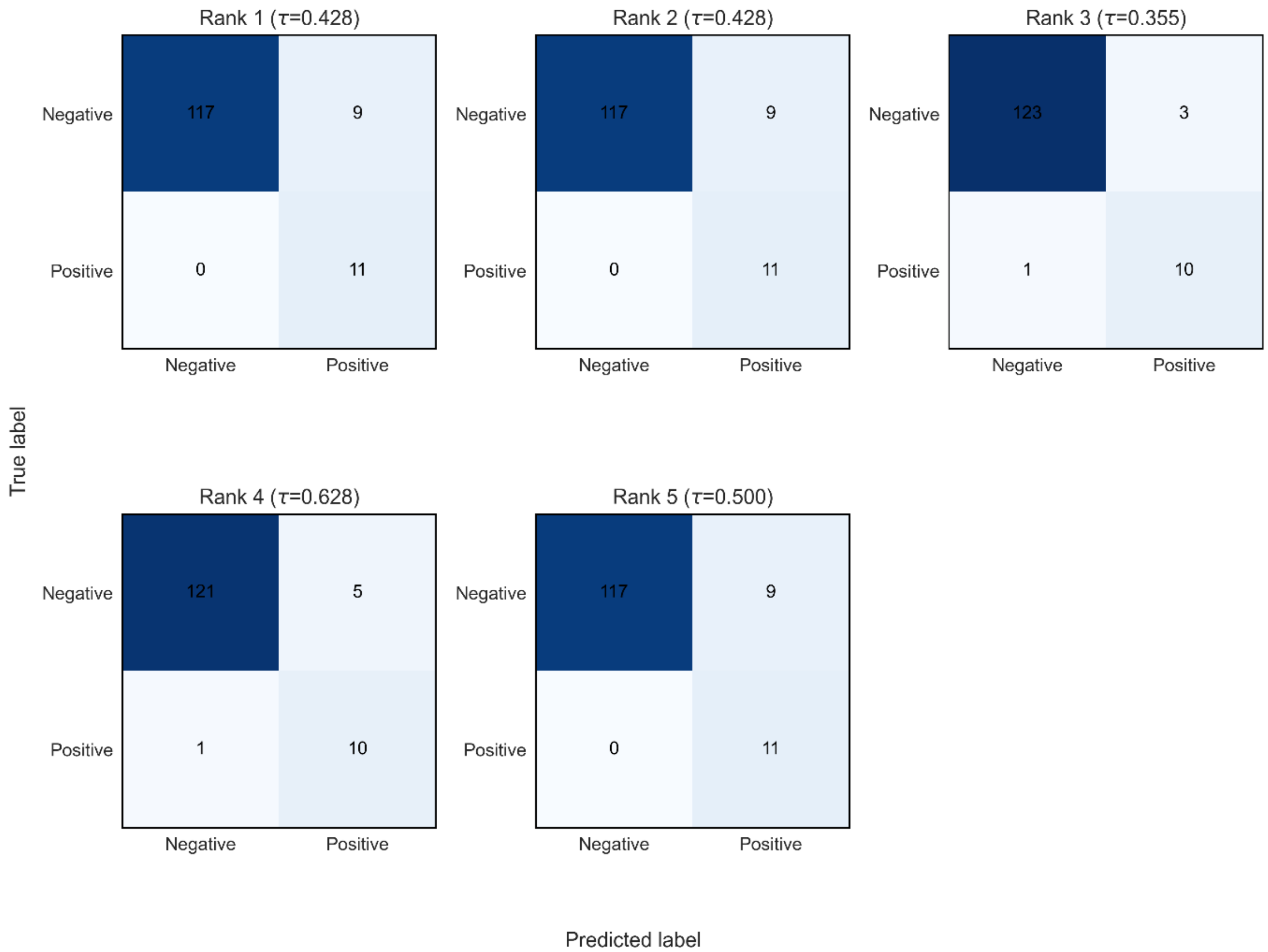

Figure S5. **Confusion matrices of the top five ranked models evaluated on the external validation set.** Each panel shows the confusion matrix from one representative external evaluation (seed = 42) among the repeated seed-specific model runs, illustrating the single-run classification outcomes of the Rank 1 to Rank 5 models on the fixed external-validation hold-out set. The cell values correspond to true negatives (top left), false positives (top right), false negatives (bottom left), and true positives (bottom right). The corresponding decision thresholds (τ) applied to each model are reported in the panel titles. These results represent one representative realization and are complementary to the aggregated performance metrics and robustness analyses presented elsewhere.

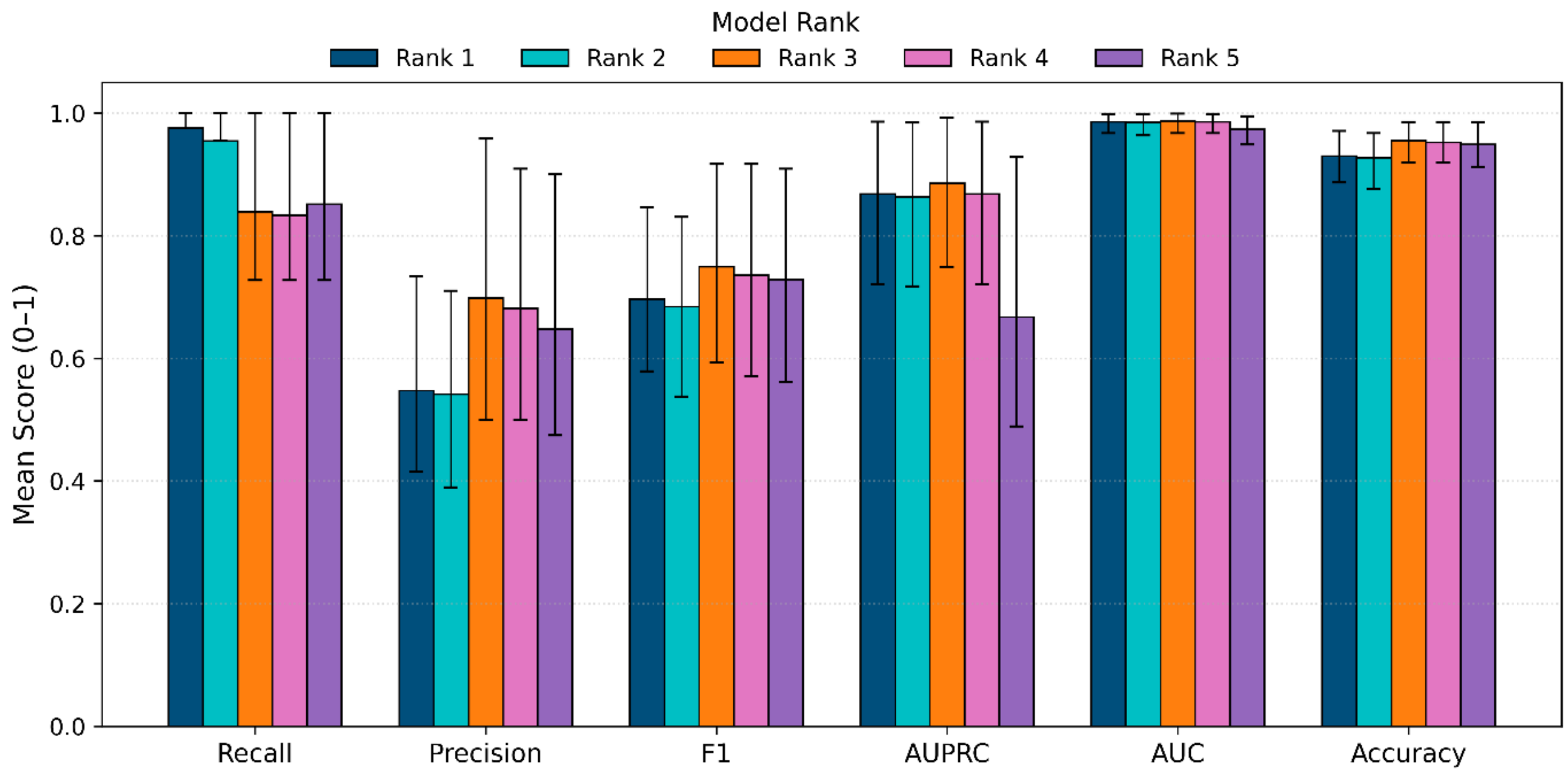

Figure S6. Mean-only performance comparison of the five selected pipelines. Bar plots summarize the mean values of recall, classification precision, F1-score, AUPRC, AUC, and accuracy for the five top-ranked models evaluated on the fixed external-validation hold-out set. Error bars represent the variability across random seeds. Although the average performance metrics appear broadly similar across the ensembles, this visualization does not capture the pronounced differences in statistical stability that are revealed by the seed-level distributions and bootstrap-derived confidence intervals shown in Figure 2. Accordingly, this figure serves as a complementary illustration highlighting the limitations of mean-only performance comparisons when assessing the robustness of chemometric pipelines.

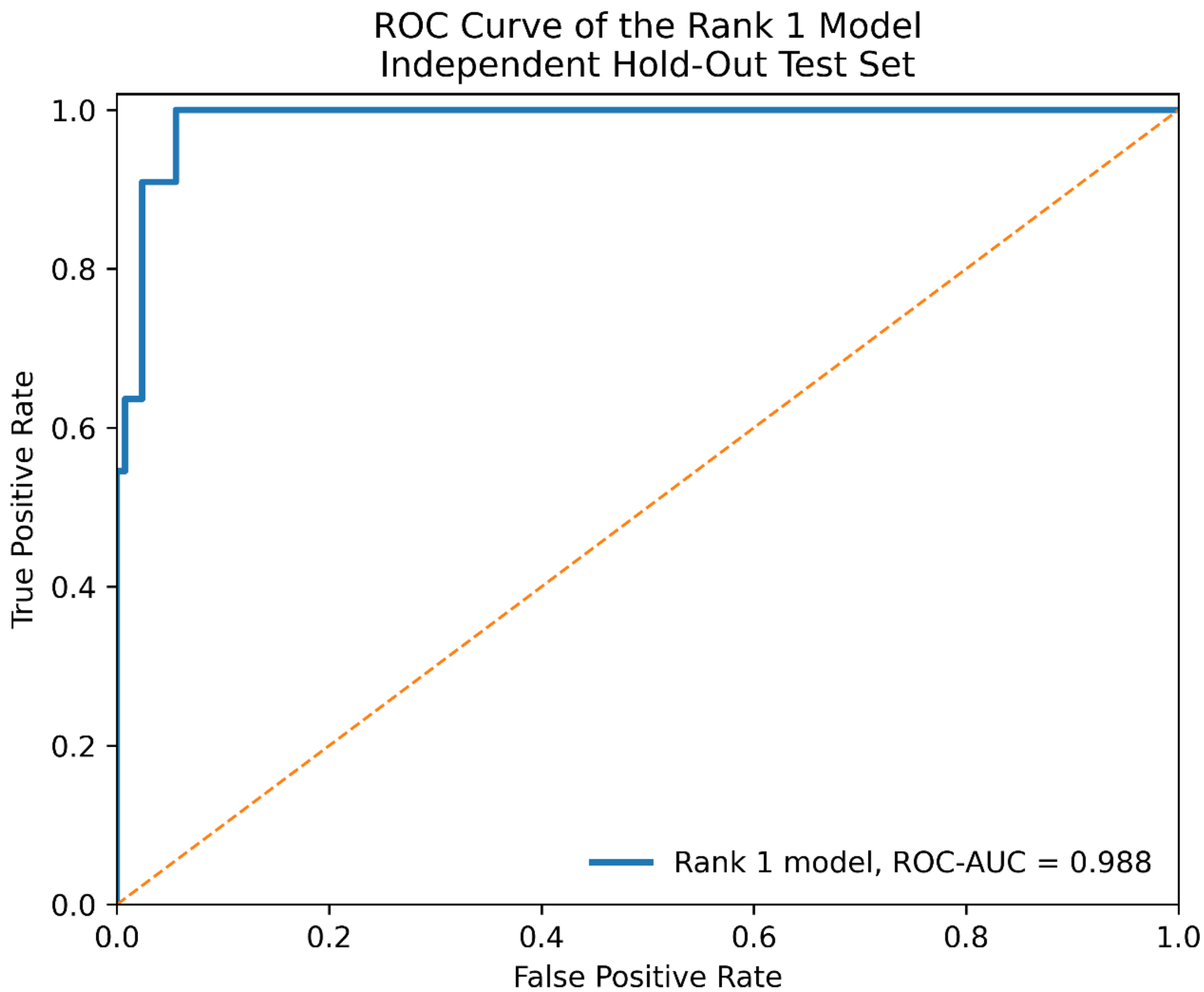

Figure S7. Receiver operating characteristic curve of the fixed Rank 1 model on the fixed external-validation hold-out set. The curve was generated from predicted positive-class probabilities, and the dashed diagonal line indicates the no-discrimination reference. ROC–AUC = 0.988, indicating high threshold-independent discrimination in the fixed external-validation hold-out set.

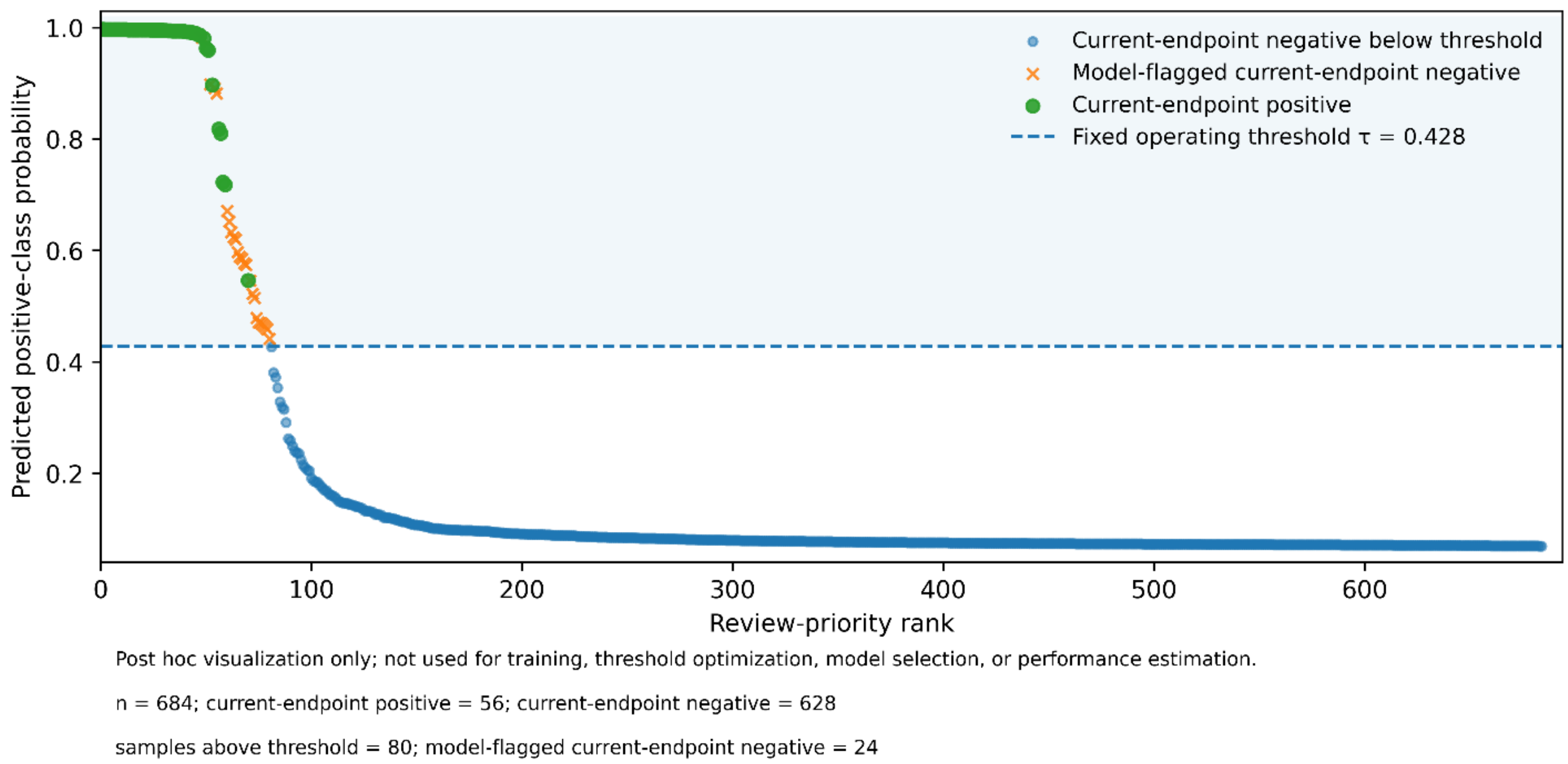

Figure S8. Post hoc review-priority ranking of all 684 samples using the fixed Rank 1 model. Samples were ranked in descending order according to the predicted positive-class probability generated by the fixed Rank 1 model (seed = 42). The dashed blue horizontal line indicates the corresponding fixed operating threshold (τ = 0.428), and samples above this threshold were flagged by the model for review prioritization. Marker colors and symbols indicate the current GC–MS/MS endpoint reference labels and model-flag status: current-endpoint negative samples below the threshold (blue circles), model-flagged current-endpoint negative samples (orange x-shaped markers), and current-endpoint positive samples (green circles). Among all 684 samples, 56 were current-endpoint positive and 628 were current-endpoint negative; 80 samples exceeded the fixed threshold, including 24 model-flagged current-endpoint negative samples. This post hoc visualization was used only to inspect model-prioritization behavior and was not used for model training, threshold optimization, model selection, or independent performance estimation.

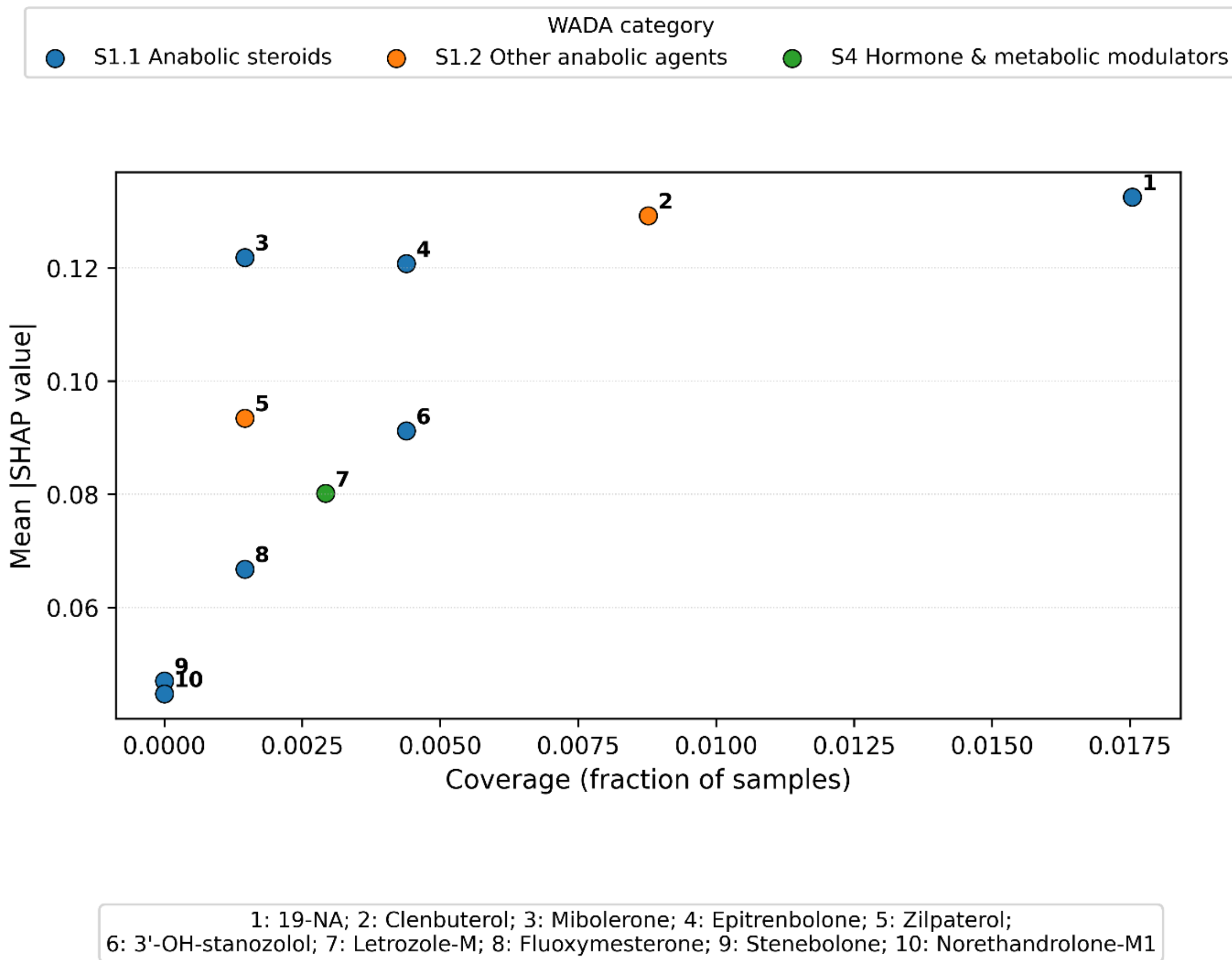

Figure S9. Relationship between feature importance and coverage of key doping-related analytes. Scatter plot illustrating the relationship between mean absolute SHAP values and coverage for the top ten most influential analytes in the Rank 1 model. The x-axis represents coverage, defined as the fraction of samples with non-zero analyte intensity for each feature, while the y-axis denotes the mean absolute SHAP value, reflecting the global contribution of each analyte to the model output. Points are color-coded according to the corresponding WADA prohibited substance categories. The absence of a monotonic relationship indicates that model importance is not solely driven by coverage, highlighting that both relatively higher- and lower-coverage analytes can exert substantial discriminative influence.
